# Is there a relationship between psoriasis and hepatitis C? A meta-analysis and bioinformatics investigation

**DOI:** 10.1186/s12985-021-01606-z

**Published:** 2021-07-02

**Authors:** Yong Liu, Sheng Nan Cui, Meng Yao Duan, Zhi Li Dou, Yi Zhen Li, Yi Xing Liu, Ye Xia, Jia Wei Zhang, Xiao Ning Yan, Dong Ran Han

**Affiliations:** 1 School of Life Science, Beijing University of Chinese Medicine, Beijing, China; 2 Department of Dermatology, China Academy of Chinese Medical Science, Xiyuan Hospital, Beijing, China; 3 Department of Dermatology, Shaanxi Hospital of Chinese Medicine, Xi’an, China

**Keywords:** Psoriasis, Hepatitis C, Inflammatory response, Toll-like receptor signaling pathway

## Abstract

**Background:**

The relationship between psoriasis and hepatitis C was previously controversial, so our purpose is to investigate this connection.

**Methods:**

We conducted a systematic review of the case–control, cross-sectional and cohort studies examining the association between psoriasis and hepatitis C in PubMed, EMBASE and Cochrane library databases and investigated the overlapping genes between psoriasis targets and hepatitis C targets using bioinformatics analysis. Based on overlapping genes and hub nodes, we also constructed the protein–protein interaction (PPI) network and module respectively, followed by the pathway enrichment analysis.

**Results:**

We included 11 publications that reported a total of 11 studies (8 cross-sectional and 3 case–control). The case–control and cross-sectional studies included 25,047 psoriasis patients and 4,091,631 controls in total. Psoriasis was associated with a significant increase of prevalent hepatitis C (OR 1.72; 95% confidence interval [CI] (1.17–2.52)). A total of 389 significant genes were common to both hepatitis C and psoriasis, which mainly involved IL6, TNF, IL10, ALB, STAT3 and CXCL8. The module and pathway enrichment analyses showed that the common genes had the potential to influence varieties of biological pathways, including the inflammatory response, cytokine activity, cytokine–cytokine receptor interaction, Toll-like receptor signaling pathway, which play an important role in the pathogenesis of hepatitis C and psoriasis.

**Conclusion:**

Patients with psoriasis display increased prevalence of hepatitis C and the basic related mechanisms between hepatitis C and psoriasis had been preliminarily clarified.

**Supplementary Information:**

The online version contains supplementary material available at 10.1186/s12985-021-01606-z.

## Introduction

Psoriasis is a chronic systemic inflammatory disease that affects approximately 0.5% to 11% of adults worldwide and is considered to be a T cell mediated disease [1, 2]. While psoriasis can be triggered by infection, trauma, stress, drugs, genetics and environmental factors, the pathogenesis of psoriasis remains unknown [3]. Clinical manifestations include skin scales and erythema, pruritus, arthritis, nail deformity and liver abnormalities [4–6].

Hepatitis C virus (HCV) infection is a global disease and the 2015 global prevalence estimate of 1.0% or 71.1 million infections [7]. After HCV infection, the liver parenchymal cells will produce and release a large amount of thymic interstitial lymphocytes, thus stimulating the proliferation and differentiation of T helper cell 17(Th17) cells. Th17 cells can secrete interleukin (IL) -17 and inhibit the apoptosis of liver cells, promoting the survival of HCV-infected cells, thus causing the chronic development of HCV infection [8–10].

Previous studies have shown that HCV may cause psoriasis through upregulation of antimicrobial peptides Toll-like receptor 9 (TLR9) and interferon, which are involved in the development of psoriasis [11]. However, the relationship between HCV infection and psoriasis remains unclear. The purpose of this study is to systematically investigate the relationship between hepatitis C and psoriasis, as well as the molecular mechanisms.

## Methods

### Search strategy and selection criteria

We conducted a systematic review and meta-analysis of observational studies on the association between psoriasis and hepatitis C, which was studied in accordance with the Preferred Reporting Items for Systematic Reviews and Meta-Analyses (PRISMA) [12] and Meta-analysis of Observational Studies in Epidemiology (MOOSE) guidelines [13].

For this meta-analysis, we searched PubMed, EMBASE and Cochrane library on December 31, 2020 without language or publication date restrictions, all relevant clinical studies were included in our study. Using the following keyword combinations: ("psoriasis" OR "psoriases" OR "psoriasi") AND ("hepatitis C" OR "hepacivirus"). We also searched the reference lists, relevant reviews and meta-analysis of included studies to find other potential studies. After searching the electronic database, studies were selected based on pre-established inclusion criteria. (1) Observational studies examining the correlation between psoriasis and hepatitis C, including cross-sectional studies, case–control studies or cohort studies; (2) The study participants are humans; (3) The case group is composed of patients with psoriasis, and the control group is composed of individual composition without psoriasis; (4) Studies must have provided odds ratios (ORs) or sufficient raw data to calculate ORs. Research screening was conducted by two independent investigators based on the above criteria, and a third investigator arbitrated whether the study was suitable for inclusion. First, the investigator independently screened all titles and abstracts to exclude duplicate and obviously irrelevant articles. Second, we checked the full texts of all remaining papers and included studies that met the inclusion criteria.

### Data extraction and risk of bias assessment

The following data were extracted from the included studies: first author, year of publication, country, study design, and quantitative estimates including odds ratio (OR) with 95%CIs on the association of hepatitis C with psoriasis. We used the Newcastle–Ottawa Scale to assess the risk of bias of included studies [14]. A study could be rated as low risk, uncertain risk, or high risk based on its effects, a study with one or more red lights indicated a lower-quality study, while a study with no red lights suggested a higher-quality study (Additional file 1). All included studies were independently evaluated by two investigators, and differences were resolved through discussions with the third investigator.

### Statistical analysis

All analyses were conducted by using the Review Manager 5.3 [15]. Odds ratios (OR) were calculated for case control/cross-sectional studies. The I^2^ statistic was used to assess the statistical heterogeneity of the included studies. I^2^ value greater than 50% was considered to performed a random-effects model meta-analysis and using fixed effects model meta-analysis with I^2^ less than 50%. All statistical tests were two-sided, and a p value < 0.05 was considered statistically significant.

### Retrieval of potential disease targets

Using keywords “hepatitis C” and “psoriasis” to search for protein targets of hepatitis C and psoriasis-related diseases in the DisGeNET and GeneCards databases. GeneCards (https://www.genecards.org/) knowledge base automatically integrates gene-centric data from 150 network resources including genome, transcriptome, proteome, genetic, clinical, and functional information. It is an integrable, searchable database and provides comprehensive, user-friendly information about all annotated and predicted human genes. DisGeNET (http://www.disgenet.org/) is a discovery platform containing one of the largest publicly available collections of genes that can be used for different research purposes, including the investigation of the molecular underpinnings of human diseases and their comorbidities, the analysis of the properties of disease genes etc. [16]. Furthermore, the intersection of hepatitis C target and psoriasis target was determined with the online tool Venny 2.1 (http: // bioinfogp.cnb.csic.es/tools/venny/index.html).

### Construction and analysis of the protein–protein interaction (PPI) network

The STRING database (https://string-db.org/) covers almost all functional interactions between expressed proteins [17], and the species can be set to homo sapiens to construct a protein–protein interaction (PPI) network. Importing the target interaction information results obtained from the analysis into Cytoscape (version 3.6.1; (https://www.cytoscape.org/) where the interactive network is drawn and analyzed [18]. The database defines PPI with confidence ranges for data scores (low confidence: scores > 0.15; medium > 0.4; and high: > 0.7). In the present study, PPIs with combined scores higher than 0.4 were selected for further research. At the same time, we can use the Cytoscape plugin cytohubba plug-in according to our previous research to select a degree greater than twice the median degree of all nodes as the hub to identify the central element of the biological network [19].

### Construction of gene enrichment analysis

In order to better understand the co-expression process related to hepatitis C and psoriasis, we performed Gene Ontology (GO) item and Kyoto Encyclopedia of Genes and Genomes (KEGG) pathway enrichment analysis of co-expressed genes. GO enrichment analysis provides a structured network of three aspects: biological process (BP), molecular function (MF) and cell composition (CC) to describe gene attributes. KEGG (http://www.genome.jp/kegg/) is a database for large-scale systematic analysis of gene or protein molecular interaction networks [20]. DAVID bioinformatics resources include a comprehensive biological knowledge base and analysis tools designed to systematically extract biological meaning from large gene or protein lists [21]. We use the web-based search engine DAVID to determine over-represented GO terms and KEGG pathways with enrichment scores > 2, counts > 5, and P < 0.05 threshold. Using the free online data analysis platform imageGP (http: //www. ehbio. com/ ImageGP/ index. php/ Home/Index/index.html) tool to draw the bubble chart of the GO and KEGG analysis results. The important path items of KEGG have been mapped to the bubble chart. Larger and redder bubbles represent those highly abundant path items.

## Results

### Characteristics of included studies

**Fig. 1:**
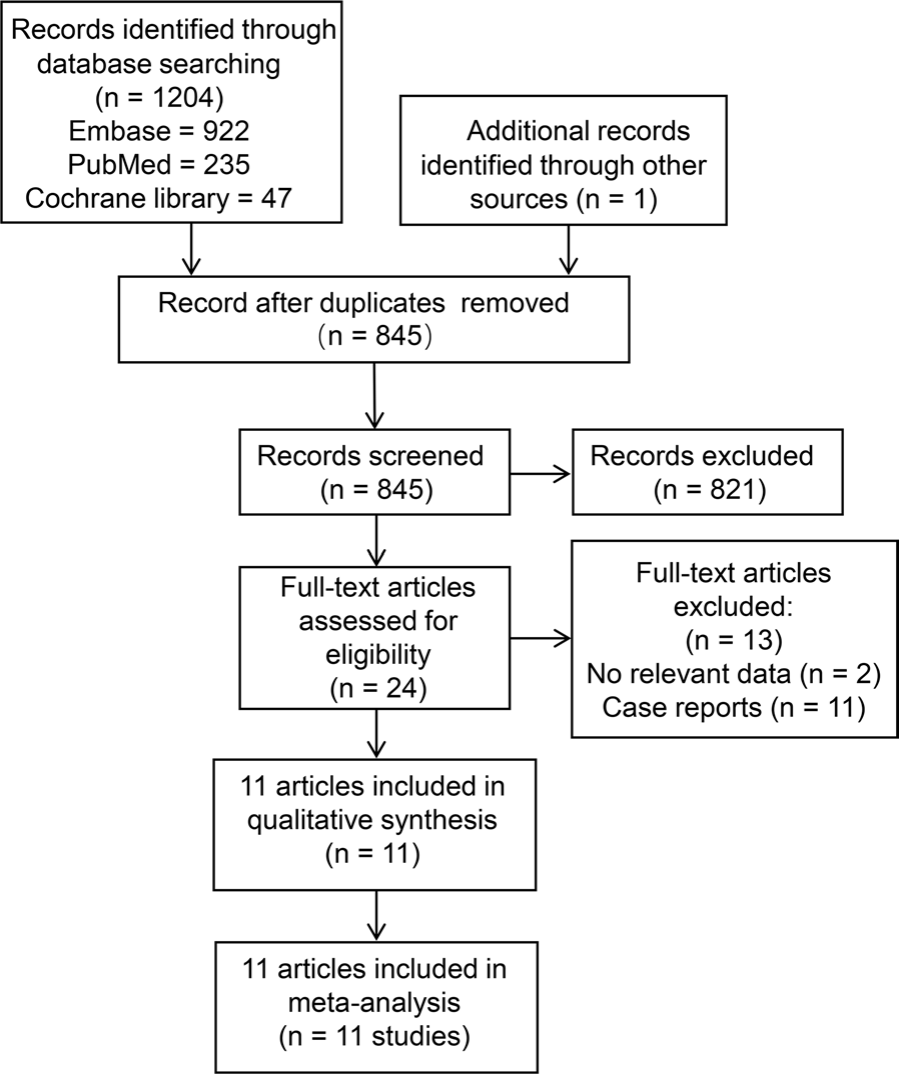
PRISMA study flowchart

**Fig. 2:**
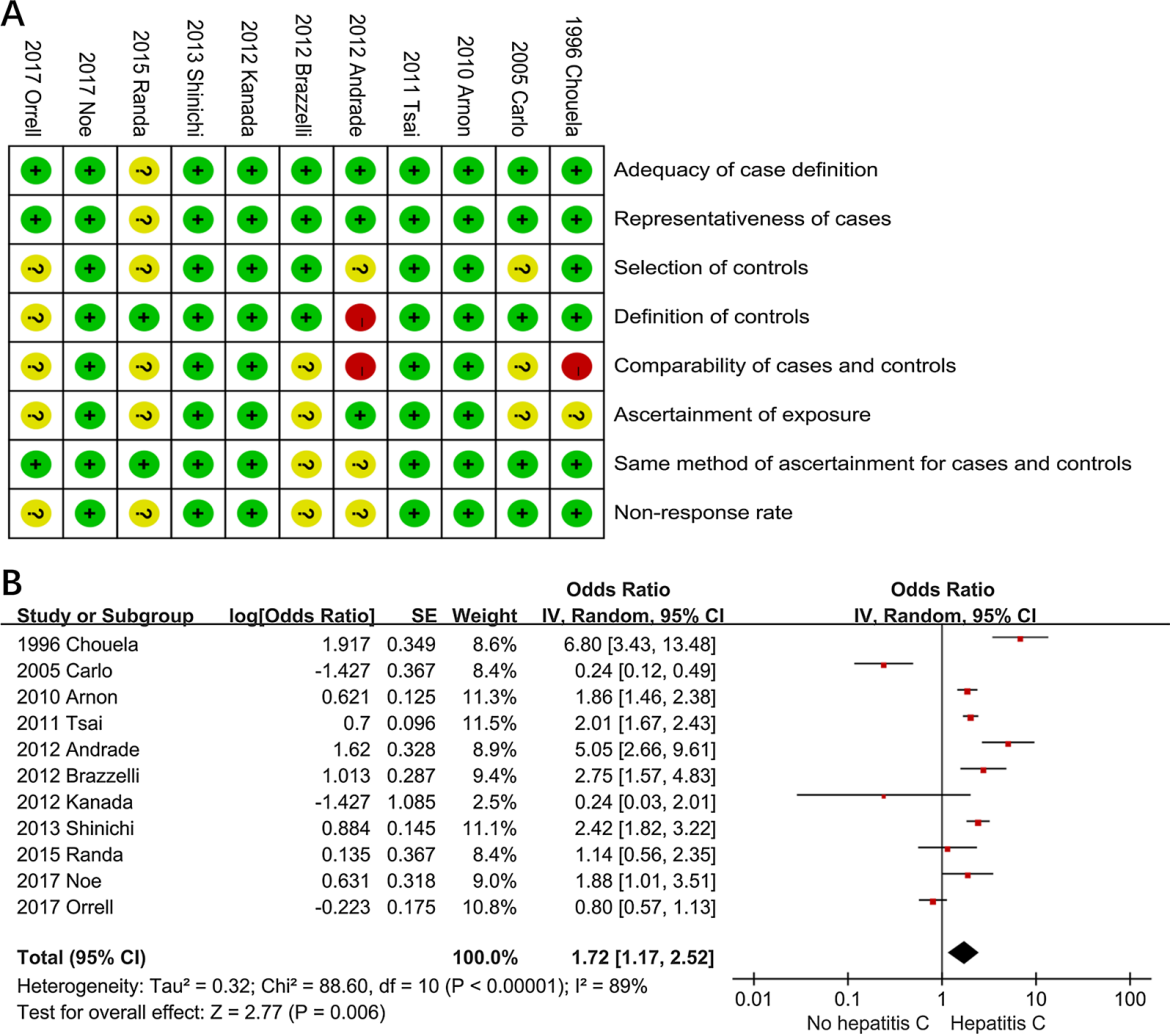
**A** Risk of bias assessment of included observational studies based on customized Newcastle–Ottawa Scale. **B** Forest plots showing significant prevalence of hepatitis C in psoriasis patients (odds ratio = 1.72, 95%, confidence interval 1.17 to 2.52)

After screening 1205 records from preliminary search, a total of 11 studies were finally included in the study [22–32]. The study selection process is summarized using the PRISMA flow chart in Fig. 1. 11 studies (8 cross-sectional and 3 case–control) examined the association of hepatitis C in psoriasis patients, as shown in Table 1. Risk of bias assessment revealed nine studies of higher quality [23–25, 27–32] and two studies of lower quality, as shown in Fig. 2A [22, 26].

**Table 1:** Characteristics of included studies

Source	Study location	Study Design	Case Group		Control Group		OR (95% CI)
			Psoriasis cases	Hepatitis C cases (%)	Control cases	Hepatitis C cases (%)	
Chouela et al. [22]	Argentina	Cross-sectional	118 (75 men, 43 women) patients with psoriasis	9/118 (7.6%)	60,000 blood donors	720/60000 (1.2%)	6.79 (3.43–13.46)
Carlo et al. [23]	Italy	Case–control	100 patients with psoriatic arthritis	1/100 (1%)	100 patients with from peripheral osteoarthritis (OA) or sciatica due to L4 L5 or L5 S1 herniated disc (two HCV unrelated disorders	4/100 (4%)	0.24 (0.117–0.492)
Arnon et al. [24]	Israel	Case–control	12,502 psoriasis patients	129/12502 (1.03%)	24,287 age- and sex- matched controls	135/24287 (0.56%)	1.86 (1.46–2.38)
Tsai et al. [25]	Taiwan	Case–control	51,800 Patients with psoriasis (31 923 males and 19,877 females)	184/51800 (0.36%)	207,200 Controls matched for age, sex, and urbanization level of residential area	404/207200 (0.19%)	2.02 (1.67–2.43)
Andrade et al. [26]	Brasil	Cross-sectional	140 patients with psoriasis	10/140 (7.1%)	2,675,000 people in the city of Salvador	40,125/2675000 (1.5%)	5.05 (2.655–9.611)
Kanada et al. [27]	US	Cross-sectional	148 patients with psoriasis	1/148 (0.6%)	5717 Non-psoriasis	125/5717 (2.2%)	0.24 (0.03–2.01)
Brazzelli et al. [28]	Italy	Cross-sectional	168 patients with psoriasis	14/168 (7.7%)	6917 people from northern Italian towns	221/6917 (3.2%)	2.75 (1.57–4.83)
Shinichi et al. [29]	Japan	Cross-sectional	717 (482 men, 235 women) patients with psoriasis	54/717 (7.5%)	38,057 (17,926 men and 20,131 women) patients with other dermatological diseases	1239/38057 (3.3%)	2.42 (1.82–3.21)
Randa et al. [30]	Egypt	Cross-sectional	100 Patients with psoriasis	19/100 (19%)	200 healthy volunteers	17/200 (8.5%)	1.14 (0.56–2.35)
Orrell et al. [31]	US	Cross-sectional	2590 people with psoriasis	34/2590 (1.3%)	112,265 people without psoriasis	1830/112265 (1.6%)	0.8 (0.57–1.13)
Noe et al. [32]	UK	Cross-sectional	188,664 Patients with mild psoriasis	320/188664 (0.17%)	961,888 matched controls	1154/961888 (0.12%)	1.88 (1.10–3.51)

### Association of psoriasis with hepatitis C in studies

A total of 11 studies, including 3 case control and 8 cross-sectional studies with 25,047 psoriasis patients and 4,091,631 controls provided data for this outcome [22–32]. We identified substantial statistical heterogeneity across these 11 studies (I^2^ = 89%). Psoriasis was associated with a significant increase of prevalent hepatitis C (OR 1.72; 95% confidence interval [CI] (1.17–2.52)) (Fig. 2B).

### Candidate genes associated with hepatitis C and psoriasis

Target genes in hepatitis C and psoriasis were obtained from the GeneCard and DisGeNET databases (Additional file 2: Table 1–4). A total of 389 significant genes were common to both hepatitis C and psoriasis, which were associated with two diseases each other (Additional file 2: Table 5).

### PPI network analysis

**Fig. 3:**
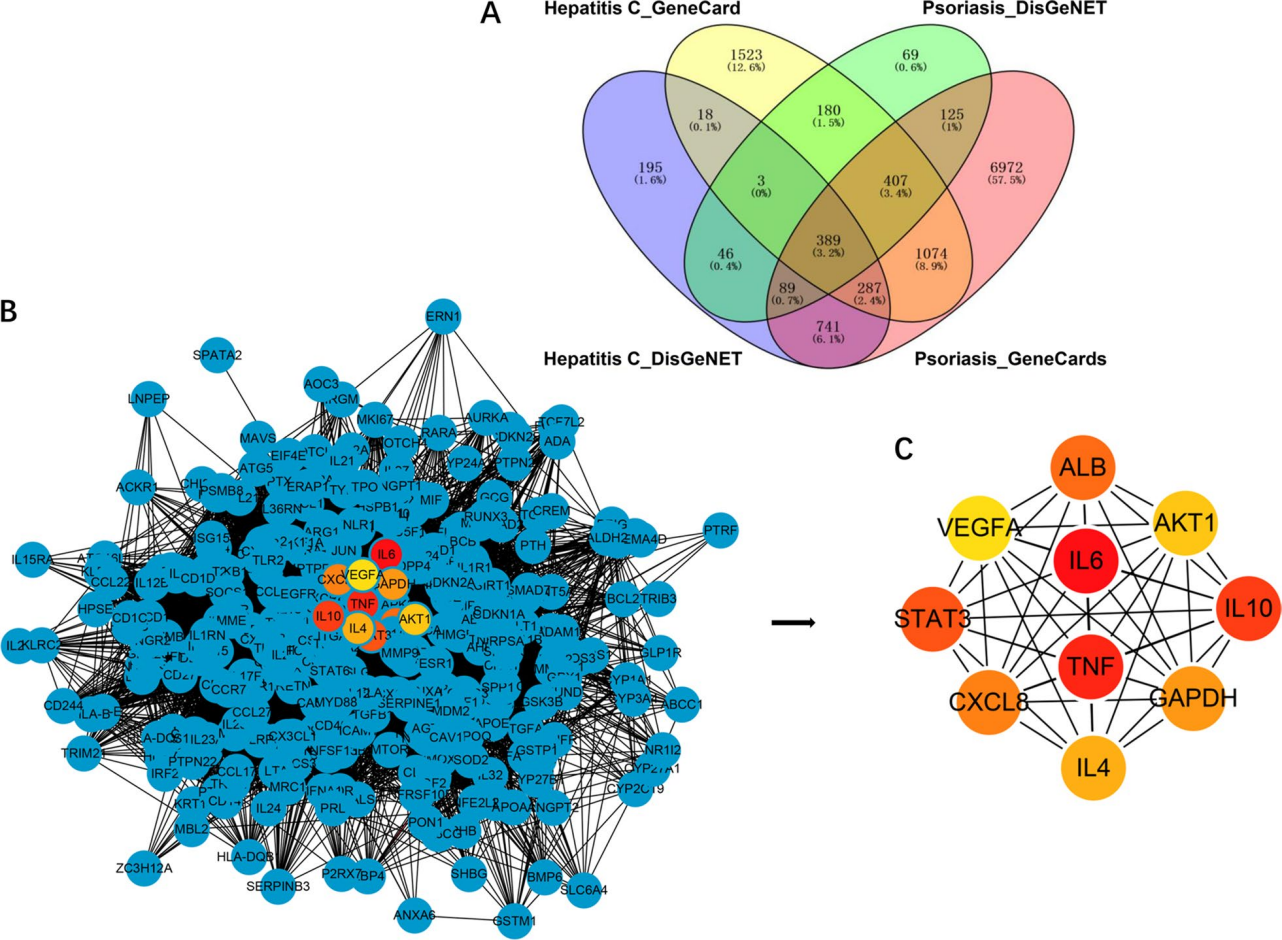
**A** The 389 overlapping genes between hepatitis C and psoriasis. **B** The results of hub nodes in current protein–protein interaction (PPI) network, the red square represented the top 10 targets, the line between two nodes represented the interaction. **C** The ranking of top 10 hub nodes based on degree. From red to yellow represented a decline in importance

A total of 389 common potential genes associated with hepatitis C and psoriasis were uploaded to the STRING database for analysis. The network of PPI was established through the STRING database and the results were used for further analysis through Cytoscape software (Fig. 3B). From the analysis results, a total of 389 nodes and 1,375 edges were acquired, and the average node degree is 66.94. The edges represent the association between a pair of action targets, the nodes represent the action target, and the degree value represents its action intensity. The top ten targets IL-6, Tumor necrosis factor (TNF), IL10, Signal transducer and activator of transcription 3 (STAT3), Albumin(ALB), C-X-C Motif Chemokine Ligand (CXCL)8, Glyceraldehyde-3-Phosphate Dehydrogenase (GAPDH), IL4, AKT Serine/Threonine Kinase 1 (AKT1) and Vascular Endothelial Growth Factor A (VEGFA) have higher degree in this process, which explained their significance in the network (Fig. 3C).

### Gene ontology enrichment analysis

**Fig. 4:**
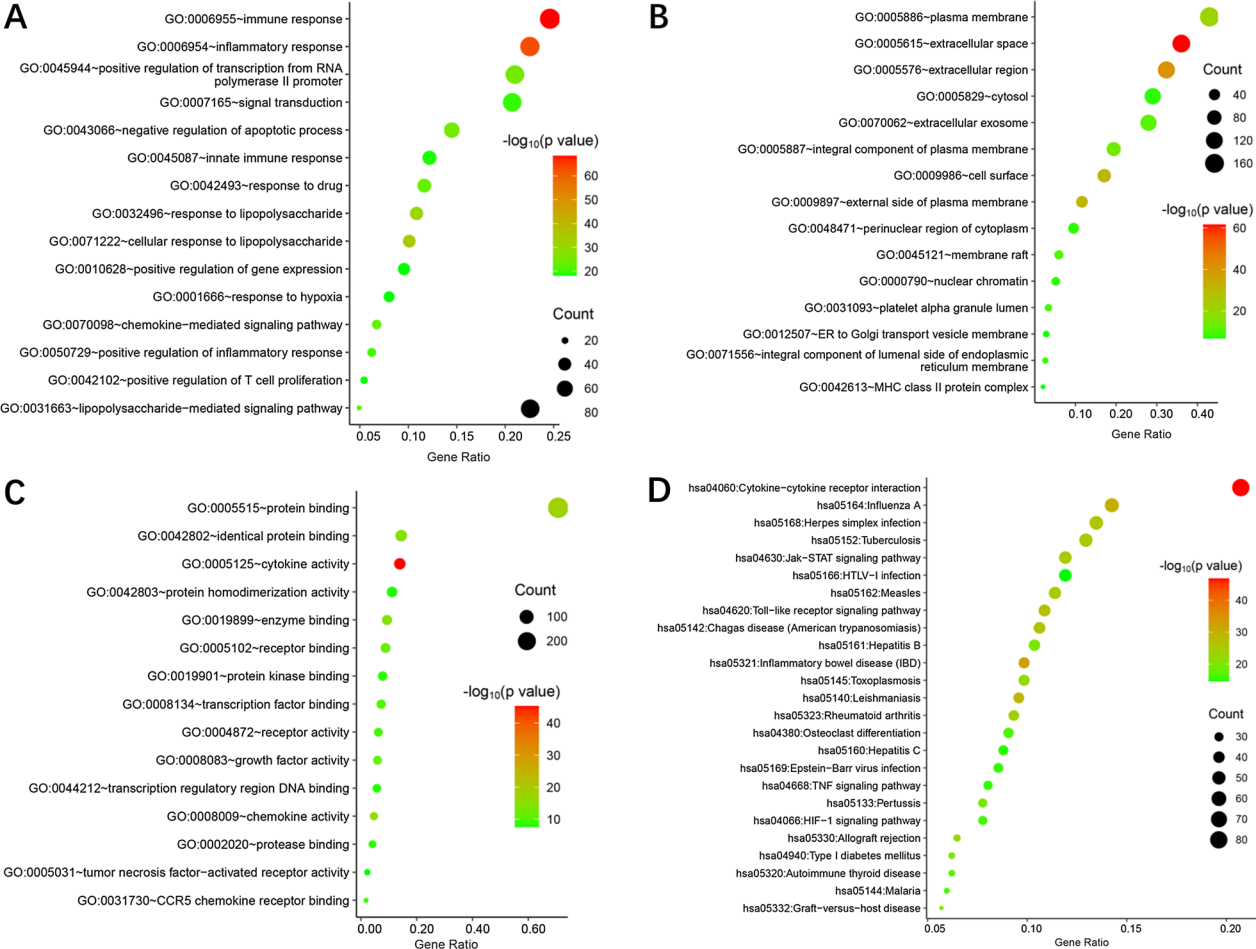
Gene ontology (GO) term enrichment including **A** biological process (BP), **B** cellular component (CC) and **C** molecular function (MF) enriched by hub genes, respectively. **D** Kyoto Encyclopedia of Genes and Genomes (KEGG) for hub genes

To verify whether the 389 genes are related to hepatitis C and psoriasis, we conducted GO enrichment analysis to clarify the relevant biological processes (Additional file 3: Table 1). The redder the color, the bigger the value of -log10 p-value which also means greater credibility and more importance. The results of the GO analysis indicated that inflammatory response, immune response, and cellular response to lipopolysaccharide were the most significant terms related to hepatitis C and psoriasis in the BP category. Furthermore, the significant terms related to hepatitis C and psoriasis were cytokine activity, protein binding, and chemokine activity. The results of GO enrichment showed in Fig. 4A–C.

### The KEGG pathway enrichment analysis

The KEGG pathway enrichment analysis was shown in Fig. 4D. Larger and redder bubbles represent the highly, significantly enriched pathway terms. In this study, the 389 overlapping gene symbols were mapped to 59 pathways following KEGG pathway enrichment. We sorted the first 25 pathways from small to large according to the p-value for a brief demonstration. The details of the KEGG pathway enrichment analysis are described in Additional file 3: Table 2. According to the results, an integrated overlapping signal pathways between psoriasis and hepatitis C has been constructed by the KEGG enrichment analysis, Of these pathways include: Immune system (e.g., Cytokine–cytokine receptor interaction, Toll-like receptor signaling pathway) (Additional file 3: Fig. 1), Signal transduction (e.g., Jak-STAT signaling pathway), Inflammatory disease (e.g., Inflammatory bowel disease (IBD)), Human disease-bacterial infectious disease pathways (e.g., Tuberculosis), human disease-viral infection (e.g., Influenza A, Herpes simplex infection, Measles), human disease-parasitic infectious disease pathway (e.g., Leishmaniasis, Chagas disease (American trypanosomiasis)). These signal pathways can affect the immune response, inflammatory response.

## Discussion

To our knowledge, this study is the first meta-analysis to examine the association of psoriasis with hepatitis C. The high risk of bias in the study is mainly related to the failure to consider many confounding factors that affect the results of the trial, but these confounding factors will not affect the positive correlation between the two diseases. We found that patients with psoriasis were prone to have comorbid hepatitis C. The evidence from case control/ cross-sectional studies indicates that patients with psoriasis had 1.72-fold increased odds of developing hepatitis C when compared with controls. However, the exact pathway leading to concurrence of both psoriasis and hepatitis C remains unclear, in the present study, we use an integrative system bioinformatics approach to clarify the relationship between psoriasis and hepatitis C, and elucidate a more exact mechanism that links these two conditions.

The results showed that 398 overlapping gene targets were obtained through PPI analysis, such as IL6, TNF, IL10 and other immune-related inflammatory factors. Many previous studies have shown that psoriasis is an immune-related disease based on T lymphocytes, which is mainly manifested in the imbalance of the balance between different cytokines and the imbalance of complex interactions in tissue disease [33]. It is well known that the inflammatory state associated with hepatitis C virus infection not only affects the liver, but also affects the entire complicated body system [34].

There are many typical pathways and molecular expressions upregulated in the skin of psoriatic lesions, including TNF/IL23/IL17A pathway, TNF, IL1A, IL1B, IL6, IL12B, IL17C, IL23A, IL36A, IL36B, IL36G, CXCL1, CXCL2, CXCL8, CXCL9, CXCL10, Cathelicidin (CAMP) and Interferon-β (IFNB) [35–45]. The occurrence, development and chronic recurrence of psoriasis are mainly due to the fact that these cells/chemokines and antimicrobial peptides promote the proliferation of keratinocytes and recruit pathogenic T cells and neutrophils [35–45].

Cytokines play a key role in the immune response to mediate disease progression and viral infection [46]. Infection with hepatitis C virus can lead to a systemic increase in the production of a variety of inflammatory cytokines, especially interleukin-6 (IL6) and tumor necrosis factor-a (TNF-α). Tumor necrosis factor is a key member of the tumor necrosis factor receptor superfamily (TNFSF), which is produced by the activation of macrophages caused by tissue damage [47, 48].

As a new type of therapy for psoriasis, biological therapy is more effective than traditional drugs, and it is safer and can significantly improve the quality of life of patients with psoriasis [49]. But the use of TNF-α inhibitors and other biological agents in patients with hepatitis C virus infection may activate viral replication and aggravate the condition of hepatitis C patients. However, some cases report and small retrospective cohort studies have found TNF-α inhibitors, especially etanercept. Generally speaking, there is no or very little risk of HCV activation. However, some guidelines suggest that dermatologists should cooperate with hepatologists to comprehensively manage patients with psoriasis associated with hepatitis C virus infection, and when using TNF-α inhibitors, it is necessary to use HCV-RNA and alanine aminotransferase (ALT) detection methods to monitor the hepatitis C status [49–51].

Toll-like receptors (TLR) are a group of evolutionary conserved pattern recognition receptors, which are found on innate immune cells in a variety of organisms [52]. TLR is the initiator of inflammation and can promote host defense, trigger signal transduction pathways, activate transcription, and synthesize pro-inflammatory cytokines and other proteins. There are 11 types of TLRs that have been discovered so far [53–55]. These cytokines recruit neutrophils and macrophages to the injury site, which activates the inflammatory cascade in turn [56, 57]. Hepatitis C virus can evade innate immune response by blocking the Toll-like receptor 3 (TLR3)-mediated interferon signaling via NS4B-induced TRIF degradation [58]. Toll-like receptor 4 (TLR4) can induce innate immune response in chronic inflammation in patients chronically infected with HCV [59]. Sobia et al. provided the foundation and proposed the use of TLR4 inhibitors in patients, who were previously resistant to INF-α ribavirin combinational therapy and treatment-naive patients, along with direct acting antivirals (DAAs), to reduce liver tissue damage [59].

The expression of Toll-like receptor 2(TLR2) and TLR4 in the outer keratinocytes and peripheral blood mononuclear cells of patients with psoriasis is increased [60, 61]. There is also an association between chronic plaque psoriasis and psoriatic arthritis with polymorphisms in TLR4 [62]. A recent study found that epidermal infiltration of neutrophils drives the inflammatory response in the skin by activating epidermal TLR4-IL36R crosstalk in a psoriasis-like mouse model induced by imiquimod [63].

Glycyrrhizic acid (GA), a triterpene isolated from the roots and rhizomes of licorice, named GA, is the principal bioactive ingredient of anti-viral, anti-inflammatory and hepatoprotective effects, and can also strongly attenuate the inflammatory response induced by TLR3 and TLR4 ligands [64]. Compound glycyrrhizin can be used to treat psoriasis [65]. Ashfaq et al. [66] illuminated that GA can inhibits HCV core gene expression or function and has a cooperative effect with INF. GA can reduce the level of transaminase in patients with hepatitis C, and the cancer rate of chronic hepatitis C patients with long-term formulation treatment is significantly reduced [67].

Since there is no cohort study, it is difficult to explore the causal relationship between hepatitis C and psoriasis. Therefore, the quality of evidence may be limited. In addition, as mentioned earlier, although hepatitis C and psoriasis may have a similar pathogenesis, it is difficult to determine whether there is a direct relationship between the two. In addition, we did not include subtypes of psoriasis because our goal is to reduce the heterogeneity of the data. In addition, these studies did not provide information to describe the risks of different ages, genders, or disease severity.

Our research suggests a relationship between hepatitis C and psoriasis. In order to further study this finding and establish stronger results, more large-scale prospective studies are needed to provide more detailed information about the link between hepatitis C and psoriasis. The prevalence of hepatitis C is increased in patients with psoriasis, and clinicians should be aware of this possibility. Targeted TLRs therapy may be helpful for the clinical treatment of psoriasis complicated by hepatitis C.

## Conclusion

Our study confirms that psoriasis is related to hepatitis C for the first time, but the direction of this association needs more large-scale prospective cohort studies to clarify. We have also determined that this connection may be related to targets such as IL-6, TNF, interleukin-10 (IL10), signal transducer and activator of transcription 3 (STAT3), albumin(ALB), etc. and various biological pathways such as Cytokine–cytokine receptor interaction, Toll-like receptor signaling pathway, etc. These research results provide data and basis for further discussion of its mechanism of action.

## Supplementary Information

**Additional file 1**. Customized Newcastle-Ottawa Scale for studies.

**Additional file 2**. Analysis results of gene distributions of psoriasis and hepatitis C.

**Additional file 3**. Functional annotation of overlapping genes between the psoriasis and hepatitis C.

## Data Availability

The datasets analyzed during the current study are available from the corresponding author on reasonable request.

## Abbreviations

Th17: T helper cell 17
PPI: Protein–protein interaction
HCV: Hepatitis C virus
PRISMA: Preferred reporting items for systematic reviews and meta-analyses
MOOSE: Meta-analysis of observational studies in epidemiology
OR: Odds ratio
ORs: Odds ratios
BP: Biological process
MF: Molecular function
CC: Cell composition
IBD: Inflammatory bowel disease
CAMP: Cathelicidin
IFNB: Interferon-β
IL: Interleukin
TNF-α: Tumor necrosis factor-a
TNFSF: Tumor necrosis factor receptor superfamily
TLR: Toll-like receptors
TLR3: Toll-like receptor 3
TLR4: Toll-like receptor 4
TLR9: Toll-like receptor 9
DAAs: Direct acting antivirals
GO: Gene ontology
ALT: Alanine aminotransferase
GA: Glycyrrhizic acid
STAT3: Signal transducer and activator of transcription 3
ALB: Albumin
CXCL: C-X-C Motif Chemokine Ligand

## References

[1] Michalek IM, Loring B, John SM (2017). A systematic review of worldwide epidemiology of psoriasis. J Eur Acad Dermatol Venereol.

[2] Elder JT, Bruce AT, Gudjonsson JE (2010). Molecular dissection of psoriasis: integrating genetics and biology. J Investig Dermatol.

[3] Melero JL, Andrades S, Arola L, Romeu A (2018). Deciphering psoriasis. A bioinformatics approach. J Dermatol Sci.

[4] Della Valle V, Maggioni M, Carrera C, Cattaneo A, Marzano AV, Damiani G (2018). A mysterious abdominal pain during active psoriasis. Intern Emerg Med.

[5] Damiani G, Franchi C, Pigatto P, Altomare A, Pacifico A, Petrou S, Leone S, Pace MC, Fiore M (2018). Outcomes assessment of hepatitis C virus-positive psoriatic patients treated using pegylated interferon in combination with ribavirin compared to new Direct-Acting Antiviral agents. World J Hepatol.

[6] Fiore M, Leone S, Maraolo AE, Berti E, Damiani G (2018). Liver illness and psoriatic patients. Biomed Res Int.

[7] Polaris Observatory HCV Collaborators (2017). Global prevalence and genotype distribution of hepatitis C virus infection in 2015: a modelling study. Lancet Gastroenterol Hepatol.

[8] Xiang M, Guo L, Ma Y, Li Y. Expression of Th17 and CD4+ CD25+ T regulatory cells in peripheral blood of acute leukemia patients and their prognostic significance. Pak J Pharm Sci. 2017;30(2(Suppl.)):619–624. 28650331

[9] Wang JM, Ma CJ, Li GY, Wu XY, Thayer P, Greer P, Smith AM, High KP, Moorman JP, Yao ZQ (2013). Tim-3 alters the balance of IL-12/IL-23 and drives TH17 cells: role in hepatitis B vaccine failure during hepatitis C infection. Vaccine.

[10] Bruno R, Sacchi P, Puoti M, Maiocchi L, Patruno SF, Cima S, Filice G (2008). Pathogenesis of liver damage in HCV-HIV patients. AIDS Rev.

[11] Chun K, Afshar M, Audish D, Kabigting F, Paik A, Gallo R, Hata T (2017). Hepatitis C may enhance key amplifiers of psoriasis. J Eur Acad Dermatol Venereol.

[12] Moher D, Liberati A, Tetzlaff J, Altman DG; PRISMA Group. Preferred reporting items for systematic reviews and meta-analyses: the PRISMA statement. PLoS Med. 2009;6(7):e1000097. 10.1371/journal.pmed.1000097 PMC2707599 19621072

[13] Stroup DF, Berlin JA, Morton SC, et al. Meta-analysis of observational studies in epidemiology: a proposal for reporting. Meta-analysis of Observational Studies in Epidemiology (MOOSE) group. JAMA. 2000;283 (15):2008–2012. 10.1001/jama.283.15.2008 10789670

[14] Wells G SB, O’Connell J, Robertson J, et al. The Newcastle- Ottawa Scale (NOS) for assessing the quality of nonrandomized studies in meta-analysis. 2011. Available from: http://www.ohri.ca/programs/clinical_epidemiology/oxford.asp.

[15] Review Manager (RevMan) Computer program.. Version 5.3. Copenhagen: the nordic cochrane centre, The Cochrane Collaboration. 2014.

[16] Piñero J, Ramírez-Anguita JM, Saüch-Pitarch J, Ronzano F, Centeno E, Sanz F, Furlong LI (2020). The DisGeNET knowledge platform for disease genomics: 2019 update. Nucleic Acids Res.

[17] Szklarczyk D, Franceschini A, Wyder S, Forslund K, Heller D, Huerta-Cepas J, Simonovic M, Roth A, Santos A, Tsafou KP, Kuhn M, Bork P, Jensen LJ, von Mering C. STRING v10: protein-protein interaction networks, integrated over the tree of life. Nucleic Acids Res. 2015;43(Database issue):D447–52. 10.1093/nar/gku1003 PMC4383874 25352553

[18] Shannon P, Markiel A, Ozier O, Baliga NS, Wang JT, Ramage D (2003). Cytoscape: a software environment for integrated models of biomolecular interaction networks. Genome Res.

[19] Chin CH, Chen SH, Wu HH, Ho CW, Ko MT, Lin CY. cytoHubba: identifying hub objects and sub-networks from complex interactome. BMC Syst Biol. 2014;8 Suppl 4(Suppl 4):S11. 10.1186/1752-0509-8-S4-S11 PMC4290687 25521941

[20] Ogata H, Goto S, Sato K, Fujibuchi W, Bono H, Kanehisa M (1999). KEGG: kyoto encyclopedia of genes and genomes. Nucleic Acids Res.

[21] da Huang W, Sherman BT, Lempicki RA (2009). Systematic and integrative analysis of large gene lists using DAVID bioinformatics resources. Nat Protoc.

[22] Chouela E, Abeldaño A, Panetta J, Ducard M, Neglia V, Sookoian S, Kina M, Castaño G, Vereytou F, Frider B (1996). Hepatitis C virus antibody (anti-HCV): prevalence in psoriasis. Int J Dermatol.

[23] Palazzi C, Olivieri I, D'Amico E, D'Agostino L, Nicolucci A, Pennese E, Petricca A (2005). Hepatitis C virus infection in psoriatic arthritis. Arthritis Rheum.

[24] Cohen AD, Weitzman D, Birkenfeld S, Dreiher J (2010). Psoriasis associated with hepatitis C but not with hepatitis B. Dermatology.

[25] Tsai TF, Wang TS, Hung ST, Tsai PI, Schenkel B, Zhang M, Tang CH (2011). Epidemiology and comorbidities of psoriasis patients in a national database in Taiwan. J Dermatol Sci.

[26] Andrade DL, de Oliveira Mde F, de Souza TF, Lima RA, Bomfim EA, Rêgo VR, Paraná R, Schinoni MI. Estudio sobre la infección por el virus de la hepatitis C en pacientes con psoriasis de un centro de referencia de Brasil A study about hepatitis C virus infection in patients with psoriasis in a Brazilian reference center.. Acta Gastroenterol Latinoam. 2012;42(4):285–90. Spanish. 23383522

[27] Kanada KN, Schupp CW, Armstrong AW (2013). Association between psoriasis and viral infections in the United States: focusing on hepatitis B, hepatitis C and human immunodeficiency virus. J Eur Acad Dermatol Venereol.

[28] Brazzelli V, Carugno A, Alborghetti A, Cananzi R, Sangiovanni L, Barbarini G, De Silvestri A, Borroni RG (2012). Hepatitis C infection in Italian psoriatic patients: prevalence and correlation with patient age and psoriasis severity. J Eur Acad Dermatol Venereol.

[29] Imafuku S, Naito R, Nakayama J (2013). Possible association of hepatitis C virus infection with late-onset psoriasis: a hospital-based observational study. J Dermatol.

[30] Youssef R, Abu-Zeid O, Sayed K, Osman S, Omran D, El Shafei A, Ghaith D (2015). Hepatitis C infection in Egyptian psoriatic patients: prevalence and correlation with severity of disease. Iran J Public Health.

[31] Orrell KA, Vakharia PP, Hagstrom EL, Brieva J, West DP, Nardone B (2017). Prevalence of chronic hepatitis B and C in psoriasis patients: a cross-sectional study in a large US population. J Am Acad Dermatol.

[32] Noe MH, Grewal SK, Shin DB, Ogdie A, Takeshita J, Gelfand JM (2017). Increased prevalence of HCV and hepatic decompensation in adults with psoriasis: a population-based study in the United Kingdom. J Eur Acad Dermatol Venereol.

[33] Martins AM, Ascenso A, Ribeiro HM, Marto J (2020). The Brain–skin connection and the pathogenesis of psoriasis: a review with a focus on the serotonergic system. Cells.

[34] Sevastianos VA, Voulgaris TA, Dourakis SP (2020). Hepatitis C, systemic inflammation and oxidative stress: correlations with metabolic diseases. Expert Rev Gastroenterol Hepatol.

[35] Hawkes JE, Chan TC, Krueger JG (2017). Psoriasis pathogenesis and the development of novel targeted immune therapies. J Allergy Clin Immunol.

[36] Lowes MA, Russell CB, Martin DA, Towne JE, Krueger JG (2013). The IL-23/T17 pathogenic axis in psoriasis is amplified by keratinocyte responses. Trends Immunol.

[37] Dorschner RA, Pestonjamasp VK, Tamakuwala S, Ohtake T, Rudisill J, Nizet V, Agerberth B, Gudmundsson GH, Gallo RL (2001). Cutaneous injury induces the release of cathelicidin anti-microbial peptides active against group A Streptococcus. J Investig Dermatol.

[38] Ferrari SM, Ruffilli I, Colaci M, Antonelli A, Ferri C, Fallahi P (2015). CXCL10 in psoriasis. Adv Med Sci.

[39] Goebeler M, Toksoy A, Spandau U, Engelhardt E, Bröcker EB, Gillitzer R (1998). The C-X-C chemokine Mig is highly expressed in the papillae of psoriatic lesions. J Pathol.

[40] Guttman-Yassky E, Krueger JG (2018). IL-17C: a unique epithelial cytokine with potential for targeting across the spectrum of atopic dermatitis and psoriasis. J Investig Dermatol.

[41] Johnston A, Xing X, Guzman AM, Riblett M, Loyd CM, Ward NL, Wohn C, Prens EP, Wang F, Maier LE, Kang S, Voorhees JJ, Elder JT, Gudjonsson JE (2011). IL-1F5, -F6, -F8, and -F9: a novel IL-1 family signaling system that is active in psoriasis and promotes keratinocyte antimicrobial peptide expression. J Immunol.

[42] Johnston A, Xing X, Wolterink L, Barnes DH, Yin Z, Reingold L, Kahlenberg JM, Harms PW, Gudjonsson JE (2017). IL-1 and IL-36 are dominant cytokines in generalized pustular psoriasis. J Allergy Clin Immunol.

[43] Li H, Li H, Huo R, Wu P, Shen Z, Xu H, Shen B, Li N (2017). Cyr61/CCN1 induces CCL20 production by keratinocyte via activating p38 and JNK/AP-1 pathway in psoriasis. J Dermatol Sci.

[44] Saggini A, Chimenti S, Chiricozzi A. IL-6 as a druggable target in psoriasis: focus on pustular variants. J Immunol Res. 2014;2014:964069. 10.1155/2014/964069 PMC4122019 25126586

[45] Zhang LJ, Sen GL, Ward NL, Johnston A, Chun K, Chen Y, Adase C, Sanford JA, Gao N, Chensee M, Sato E, Fritz Y, Baliwag J, Williams MR, Hata T, Gallo RL (2016). Antimicrobial peptide LL37 and MAVS signaling drive interferon-β production by epidermal keratinocytes during skin injury. Immunity.

[46] Bader El Din NG, Farouk S, El-Shenawy R, Ibrahim MK, Dawood RM, Elhady MM, Salem AM, Zayed N, Khairy A, El Awady MK. Tumor necrosis factor-α -G308A polymorphism is associated with liver pathological changes in hepatitis C virus patients. World J Gastroenterol. 2016;22(34):7767–77. 10.3748/wjg.v22.i34.7767 PMC5016377 27678360

[47] Dostert C, Grusdat M, Letellier E, Brenner D (2019). The TNF family of ligands and receptors: communication modules in the immune system and beyond. Physiol Rev.

[48] Dawood RM, Salum GM, Abd El-Meguid M, Shemis M, Abdel Aziz AO, El Awady MK. Recipient interleukin 6 gene polymorphism and expression predict HCV recurrence post liver transplantation. Gene. 2020;754:144887. 10.1016/j.gene.2020.144887 32534059

[49] Piaserico S, Messina F, Russo FP (2019). Managing psoriasis in patients with HBV or HCV infection: practical considerations. Am J Clin Dermatol.

[50] Furst DE, Keystone EC, Kirkham B, et al. Updated consensus statement on biological agents for the treatment of rheumatic diseases, 2008. Ann Rheum Dis. 2008;67(Suppl. 3):iii2–25. 10.1136/ard.2008.100834 19022808

[51] Ding T, Ledingham J, Luqmani R, et al. BSR and BHPR rheumatoid arthritis guidelines on safety of anti-TNF therapies. Rheumatology(Oxford). 2010;49:2217–9. 10.1093/rheumatology/keq249a 20837498

[52] Rock FL, Hardiman G, Timans JC (1998). A family of human receptors structurally related to Drosophila Toll. Proc Natl Acad Sci USA.

[53] Bureau C, Bernad J, Chaouche N (2001). Nonstructural 3 protein of hepatitis C virus triggers an oxidative burst in human monocytes via activation of NADPH oxidase. J Biol Chem.

[54] Kawai T, Akira S (2006). TLR signalling. Cell Death Differ.

[55] Kawai T, Akira S (2005). Toll-like receptor downstream signalling. Arthritis Res Ther.

[56] Dolganiuc A, Kodys K, Marshall C, et al. Type III interferons, IL-28 and IL-29, are increased in chronic HCV infection and induce myeloid dendritic cell-mediated FoxP3+ regulatory T cells. PLoS One 2012;7:e44915. 10.1371/journal.pone.0044915 PMC3468613 23071503

[57] Wang JP, Zhang Y, Wei X (2010). Circulating Toll-like receptor (TLR) 2, TLR4, and regulatory T cells in patients with chronic hepatitis C. APMIS.

[58] Liang Y, Cao X, Ding Q, Zhao Y, He Z, Zhong J. Hepatitis C virus NS4B induces the degradation of TRIF to inhibit TLR3-mediated interferon signaling pathway. PLoS Pathog. 2018;14(5):e1007075. 10.1371/journal.ppat.1007075 PMC5983870 29782532

[59] Manzoor S, Khalil S, Malik MA, Shafique K, Gul S, Javed F (2020). Induction of profibrotic microenvironment via TLR4 MyD88-dependent and -independent inflammatory signaling in chronic hepatitis C virus infection. Viral Immunol.

[60] Garcia-Rodriguez S, Arias-Santiago S, Perandrés-López R, Castellote L, Zumaquero E, Navarro P, Buendía-Eisman A, Ruiz JC, Orgaz-Molina J, Sancho J, M Zubiaur. Increased gene expression of Toll-like receptor 4 on peripheral blood mononuclear cells in patients with psoriasis. J Eur Acad Dermatol Venereol. 2013;27(2):242–50. 10.1111/j.1468-3083.2011.04372.x 23457721

[61] Carrasco S, Neves FS, Fonseca MH, Gonçalves CR, Saad CG, Sampaio-Barros PD, Goldenstein-Schainberg C (2011). Toll-like receptor (TLR) 2 is upregulated on peripheral blood monocytes of patients with psoriatic arthritis: a role for a gram-positive inflammatory trigger?. Clin Exp Rheumatol.

[62] Smith RL, Hébert HL, Massey J (2016). Association of toll-like receptor 4 (TLR4) with chronic plaque type psoriasis and psoriatic arthritis. Arch Dermatol Res.

[63] Shao S, Fang H, Dang E, Xue K, Zhang J, Li B, Qiao H, Cao T, Zhuang Y, Shen S, Zhang T, Qiao P, Li C, Gudjonsson JE, Wang G (2019). Neutrophil extracellular traps promote inflammatory responses in psoriasis via activating epidermal TLR4/IL-36R crosstalk. Front Immunol.

[64] Schröfelbauer B, Raffetseder J, Hauner M, Wolkerstorfer A, Ernst W, Szolar OH. Glycyrrhizin, the main active compound in liquorice, attenuates pro-inflammatory responses by interfering with membrane-dependent receptor signalling published correction appears in Biochem J. 2009;422(3):571. Biochem J. 2009;421(3):473–482. 10.1042/BJ20082416 19442240

[65] Yu JJ, Zhang CS, Coyle ME (2017). Compound glycyrrhizin plus conventional therapy for psoriasis vulgaris: a systematic review and meta-analysis of randomized controlled trials. Curr Med Res Opin.

[66] Ashfaq UA, Masoud MS, Nawaz Z, Riazuddin S (2011). Glycyrrhizin as antiviral agent against Hepatitis C Virus. J Transl Med.

[67] GA Sun ZG, Zhao TT, Lu N, Yang YA, Zhu HL. Research Progress of Glycyrrhizic Acid on Antiviral Activity. Mini Rev Med Chem. 2019;19(10):826–832. 10.2174/1389557519666190119111125 30659537

