# Supplementary material for: Is there a relationship between psoriasis and hepatitis C? A meta-analysis and bioinformatics investigation
Source: Virol J. 2021 Jul 2;18:135. doi: 10.1186/s12985-021-01606-z (PMC8252322; doi:10.1186/s12985-021-01606-z)
Supplement: Supplementary file 1 — Additional file 1. Customized Newcastle-Ottawa Scale for studies. [file 12985_2021_1606_MOESM1_ESM.docx]

|  | **Item** | **Bias Risk** |
| --- | --- | --- |
| **Selection** | | |
| Is the case definition adequate? | Yes, with independent validation Self-report without primary reference  No description | Green  Red  Yellow |
| Representativeness of cases | Consecutive or obviously representative series of cases  Potential for selection bias  Not stated | Green  Red  Yellow |
| Selection of controls | Community controls  Inappropriate or biased controls  No description or hospital controls | Green  Red  Yellow |
| Definition of controls | No history of disease (endpoint)  History of disease  No description of source | Green  Red  Yellow |
| **Comparability** | | |
| Study controls for | Additional confounding factors + age and sex  No factors controlled for  Only age and sex or not stated | Green  Red  Yellow |
| **Exposure** | | |
| Ascertainment of exposure | Secure records or structured, blind interviews  Self-report or medical record with validation only once  Interview not blinded, self-report or medical record with validation more than once, or no description | Green  Red  Yellow |
| Same method of ascertainment for cases and controls | Yes  No  Not stated | Green  Red  Yellow |
| Non-response rate | Same rate for both groups  Rate different and no designation  Non-respondents described | Green  Red  Yellow |

**Additional File 1:** Customized Newcastle-Ottawa Scale for studies.

Green light: low risk of bias; red light: high risk of bias; yellow light: unclear risk of bias; one red light results in the study being classified as higher risk of bias
