# Supplementary material for: Is there a relationship between psoriasis and hepatitis C? A meta-analysis and bioinformatics investigation
Source: Virol J. 2021 Jul 2;18:135. doi: 10.1186/s12985-021-01606-z (PMC8252322; doi:10.1186/s12985-021-01606-z)
Supplement: Supplementary file 2 — Additional file 2. Analysis results of gene distributions of psoriasis and hepatitis C. [file 12985_2021_1606_MOESM2_ESM.docx]

**Additional File 2: Analysis results of gene distributions of psoriasis and hepatitis C.**

| **Table 1** A total of 1308 known therapeutic targets for hepatitis C were collected form the DisGeNET database. |
| --- |
| MMP2 RPTOR DONSON TRAF3IP2-AS1 TFIP11 SEA DNASE1 POLR2G GRAP2 NBAS FIBP MIR193B IL12RB1 TACC2 EDN1 CHST8 HSPB1 IL26 MIR31 CXCR4 IRAK1 IGF1 MIR146A MIR20A SLC52A1 PLAU TRAF3IP2 ADCY7 EHF NTRK1 MNAT1 PTEN ANXA1 KLK6 PRKRA MALT1 NTSR1 DELEC1 RENBP NOS2 TRIB3 CCNB1 SH3BP4 SLC6A4 SOD2 POLI HCP5 C17orf67 SYNE2 SLC22A4 AATK SEMA4D ITLN1 LINC01147 IRF1 FABP7 COPD HLA-DRB5 BANK1 CD1D MBL2 PLAAT4 RAB40B SLC12A8 NGF NR4A2 NRP1 ACAD8 CDH13 NLRP3 LEP PKD1 SERPINF1 GJA1 RPS6KA5 MAPKAPK2 CCL22 BLOC1S2 PTPN22 AKR1B10 CYP2D6 RTL1 TGM2 RUNX3 LAMP1 BSN PRKCB NFATC4 DEFB1 C5AR1 CYP2C19 TBK1 PRL TIMP3 NR1H3 ACACA TLR1 ELANE TEK CLCN6 COG6 CCL4L2 LYNX1-SLURP2 NFATC1 IGH HES1 SNRNP70 PROK2 CLC MTHFR LINC01620 PSORS5 PTPN11 LNPEP SMAD3 CD1A FOXP3 IFNL1 MIR492 CCL19 NKD1 DPP4 TRAJ23 ZNF148 CD38 MIR19A TNFRSF9 RIPK4 KLK8 FYCO1 IL17RC PLA2G2A HRH4 SMUG1 LGALS1 EZH2 APOE PF4 MUC16 HLA-E SLC25A5 ALDH2 C3 FAS HDAC7 ORM1 PON1 HSPD1 F2RL1 BDNF HBEGF INSRR MIR99A OSMR SOD3 FOXC1 SEMA3A BTRC LOC102724971 USP49 HSPB3 NM SHBG MIR223 TNFSF10 MGAT5 IL1F10 MUC1 TICAM2 BCL2 FZD2 ASIP TNFRSF11B CADM2 SON IL24 SBNO2 PLCL2 ID1 OPN4 THADA PIK3CB ZPBP2 TNXB MBTPS1 COX8A TNFSF15 PRORP CD6 TLR6 MRPL28 CXCR2 EXOC2 IFNG-AS1 F9 CCHCR1 BCAP31 MTX1 CYP1A1 STAT4 IRF3 CD40 RNF145 CAPN1 NME1-NME2 CTSV RYR1 TLCD3B RASIP1 PPBP TSC22D3 LRRK2 HTR3A ARHGEF2 APOB CFH TAP1 NRIP1 CS ITPRID1 IKZF3 SETMAR PRINS NCAM1 MIR203A LAG3 AGT ERAP1 ADGRL2 OPRK1 CAVIN1 C4A IL6R CDC6 PPARG CHI3L1 DBH JAG1 ALOX15 CRP CCL7 CALCA CXCL11 TRIM65 SERPINA1 STK40 ANTXR2 PPIG LINC02693 LINC00993 CRHR2 SPRY2 RPSA GSTP1 RUNX1 KIR3DL2 SLC9A3R1 C1orf141 MIR340 HLA-DQB1 AHCY AURKA CXCL8 MAPK9 MIR122 EGR1 CCL17 PTPN2 ECM1 GLP1R LINC02571 IL36B GRHL2 C10orf99 SELE LRRC43 TNFSF12 IL17C FN1 KLRC2 CNR1 ABCB1 AHR KRT17 DHFR NAA16 FGF19 PSORS7 UBE2N PSORS1C3 NFE2L2 IL23R TGM5 SPINK5 YWHAZ FCN2 LMO4 SCUBE1 ARMH1 IFI27 HP CCL27 SKAP2 KLRC1 VAMAS6 EMP1 TAGAP IL17A IL1RL2 IL22RA2 IFI16 GOT2 CSTA MTOR ASAP2 BMP6 VPS51 CDSN NPPA CDKN2D CARD14 IARS1 CASP5 ETFA WT1 RPE65 INS-IGF2 SNAI1 BRD4 SLC22A1 IL13RA2 SERPINB2 ZNF816-ZNF321P MAPK14 AOC3 TNFRSF4 SOX9 C4B_2 KSR1 CRABP1 MYPN DEFB4A CCN2 CETN1 SATB2 MIR424 ANXA6 CEBPD IL17D RDX CARD9 CTSB KLLN JUND CTSS ZFP36 ADCY3 ZC3H12A TNF HSPB2 DDX39A SUB1 FURIN IL23A DEFB103A EGF IL1A TMED7 CDR3 SLURP2 CCR2 UPK3B SCRN1 LORICRIN SMAD7 PARK7 FAM177A1 RNF7 ELN SELP ADRA1B TLR8 S100A14 HLA-DOA PGLYRP1 HLA-DMB TANK AOC1 PCNA DNAJB4 CSF3 REV3L HLA-A ATG5 ZGLP1 MIR210 CD27 SLIT3 NFKBIZ TNFRSF17 YDJC JAK2 CMC1 CD226 TNFRSF1A KYNU CASP1 STAT3 HTC2 SPHK1 CCL20 ATN1 SPP1 MIR17HG IL22 LINC02605 RPS26 SFTPD HCAR2 IL13RA1 WNT5A IFNGR2 IFNAR1 H3P13 OSM ARG1 NF1 LHFPL6 DUSP1 LRP5 OLR1 OPTC SPRED1 SLPI IL31 AGER TCF19 TRIM27 HOXA5 CTNNB1 CHROMR HPGDS MIR221 GPRC5A MICA IL20RA IL21R SMCP TGM3 CCR5AS FCGR3A DNMT3A DEFB104B NT5E PDC MCHR1 CD200 SPATA48 PSORS9 TPM1 IL18 OXA1L CCND1 CYP2E1 NOTCH2 ACO1 FLNB SPINK7 SULF2 CD93 CCL4 ATXN2 S100B IL15 LINC01185 SOST ESR1 TAC1 ATP5F1A SLCO1B1 TRAIP SUGP1 HSPA1A BMP4 GPX4 MIR4516 SFRP4 CDK4 ACE2 OLIG3 MCAM AGTR1 SLC12A9 IPMK MC1R CMKLR1 CAMP GRHL3 ZNF750 USP2 LINC02863 BAIAP3 IL15RA BMS1 CLMN PDE4D IL1RAP PRDX5 CCL4L1 KIR3DL1 PTX3 PDE3A CCKBR CCK FAM118A ITGB2 CDKN2B IL36G CSMD1 NXF1 PSMB8 VEGFA GATD3B STAT6 GART PIK3CD SCARB1 IL3 AREG PCDHGA12 GCHFR TGFB1 TTC7A MME S100A1 ACKR2 QPCT SPATA2 PGLYRP4 TES UBE2L3 NTS NR4A3 MIR197 TNFRSF8 BIRC2 H3P23 AAA1 SOCS3 KEAP1 MST1 ZC3H12C DAG1 JAZF1 TIMP1 S100A8 WNT10B GATD3A MGMT PI3 MRC1 CPQ MAVS TNFRSF13C PCSK9 GPR160 HT SOAT1 RAVER1 IL13 CD86 AIMP2 MIR21 TCAIM TNFRSF12A GSK3B TSPAN31 TNFAIP3 IL12A PLA2G10 LCE3B TSPAN14 FOXO1 PUS10 CCL2 MIR125A FBXL19 LINC00824 MIR187 ADA STAT1 IL27 LRRFIP1 HLA-DQA1 PTTG1 GABPA POU5F1P3 DCTN6 IL6 CAMK2G NRTN NXT1 MMP12 CDCA5 ANGPT1 MIR744 SLC2A1 SEMA6A CREM PUDPP2 CD68 CCR7 CXCL17 TEPSIN PRDM1 SLCO6A1 BSG TNFSF12-TNFSF13 ISG15 TRPV1 ISYNA1 CASP3 HSP90AA1 EFEMP1 ANGPT2 UBASH3A SCLY OR2AG1 DUOX1 ZNF831 NAP1L1 SLCO1C1 CCRL2 SLC22A5 PARP1 PSORS4 PTPRC CCR4 GNLY KIAA1109 NCKAP1 CD14 TH2LCRR VNN3 VDR LOC110806262 ACE PPARD ANXA2 PNISR ITGAM CD34 TGM1 C4B DGCR8 PDPN NCOA5 CNTN3 VASH2 PDZK1IP1 FAM20B IL19 ENG FASLG MIR126 GLIS1 INAVA KIAA1324L GGH CD163 MIR125B1 AP1S3 KDR PSMD9 MIR330 ZC4H2 KRT14 FOSL2 SYT1 ABCG2 HMOX1 CD1B FCGR2A PRKN PSORS6 CALML5 R3HCC1L TREX2 SERPINB8 ERAP2 FLT1 EDIL3 PKIG HTR1A ALOX12 RARA CARM1 EIF4E PECAM1 EBI3 MYD88 PTPN1 SMAD2 PRKCZ CRHR1 WDR11 IL20 TRAF6 LRP6 MIR145 CCR6 TLE1 PPARA CD244 CBLIF F13A1 GATA3 IL17RD ALOX15B CD59 IL2RA ZNF365 CD24 GIPC2 TBCC CXCR1 CCR1 UVRAG PTPRN2 HCG22 PSAP ACOT13 MYOM2 BST2 MMP8 COPS5 ELOA LYN POMP PCLAF RBP4 AHSA1 NME2 ADCYAP1 DMKN GSTM1 ADAM33 IFI6 CRH S100A4 IL10 CYP2S1 GPR183 MAPK1 MYRF LOXL2 PGF IGF2 NFKBIL1 BPTF CTSG UQCR10 NPEPPS TXK RARRES2 ACR KLK7 POSTN ID4 CBLL2 MIR486-1 PLA2G1B IL12RB2 CYP27B1 TRIM21 PPP3R1 RBM45 GAL3ST2 DMXL2 NAGLU GZMA POTEF FABP1 IL9R IGF2-AS PSORS1C1 PIK3CG PLAT NLN COPE ACKR1 MDM2 SDF4 LPA TNFSF11 FGF7 TAP2 IL37 GALR3 CXCL2 CLDN3 TAT EGFR NAT9 TYK2 C20orf181 TRGV5 DENND1B ATOD1 GSTK1 SPTLC2 LCE3D MIR130A MSX2P1 CD79A S1PR1 CXCR3 PIK3CA FLT3 IRGM SERPINB4 CKLF CTLA4 MIRLET7B UBAC2 FZD5 AQP3 IL2 FOXA1 VNN1 WG LYVE1 SDC1 CACNA1A TLR7 SERPINB1 RNASE7 MUL1 PLCD1 STAB1 POLDIP2 LGALS3 MXRA7 AP5B1 MYT1L GRIN1 MOK CLEC7A IFNG FOS HSPA14 EDA CHRNA4 C16orf82 IL17F RETN NAPSA JUNB TNFSF13B CFL1 IL4 CYP27A1 CD28 SELPLG NAT2 MASP2 SLC7A9 HLA-DPB1 FGF2 CLU AMH IRF5 CXCL12 CCKAR TET2 OAS2 QTRT1 RPA1 HCP5B FNDC3A KRT16 SP140 ELOVL6 MGST2 CST6 TNFSF8 CX3CL1 KHDRBS3 MAPK8 NR5A2 IGF1R RIPK1 GJB2 KLK5 KIR3DS1 GPR35 CPEB4 IFNA13 ICAM1 CXCL9 HLA-DQA2 RTEL1-TNFRSF6B THBS1 CAV1 TAL1 CD19 TNFRSF1B PSORS8 MAPK3 MIR143 MOCOS LINC02213 TRMO GZMB FCGR3B PWAR6 VIP JAM2 TAZ PDE7A CTSL ELMO1 HLA-G CA2 TPO CYBB TEX41 MYDGF ETS1 RAD50 SLC25A20 IL22RA1 ISG20 S100A2 GCKR APLN CXCL1 TRPC6 LINC00598 DEFB4B PAK5 IL25 MIR320B1 EHMT1 WDHD1 TNFRSF10B SH2B3 PLA2G7 CDK5R1 CRNN IKBKE HIF1A RELA IRF1-AS1 GRN TMED7-TICAM2 SOCS1 MIR155 TRIM32 IL17RA DUT FZD8 ARPC4 PLF GLYAT TNFRSF11A PTH TGFA NOS3 ANPEP TNFRSF14 ROBO3 ANGPTL6 FGFR1OP PTPN6 PWP1 PLXNB2 NFATC2 NPSR1 IFNLR1 TNIP1 SAA1 CYLD REG1A H3P28 MED15 FGD5 XBP1 CASR ITGAX NR0B2 CDK2 IRF2 PGR PDE4B DKK1 IL1RN SEC14L2 KRT7 NEDD8 MSMO1 NOTCH4 NRAD1 ZFYVE16 H3P9 RABEPK TFPI HAT1 AQP1 OPRM1 ACTB SCN7A LYNX1 TLR4 PRO2268 RIC8B LCE3A CXCL10 CCL21 IL16 PLG TYR TCF3 SERPINE1 EIF2AK1 IL36RN CCR10 PTTG2 TNFRSF13B POU5F1P4 MKI67 TSLP NHS STS FFAR2 MIR17 VCAM1 IL4R STAT2 CCL5 BTNL2 SLC9C2 TP53 REL PPP6C ENTPD1 HNRNPA1P10 COX2 TAS2R13 STK11 OSCP1 PSORS1C2 TLR9 IL33 SETD1A RGS6 HLA-DMA NLRP1 FLG LINC01193 KIR2DS1 IFNA1 LINC02085 SUCO CCN1 LURAP1L HLA-DRB1 PDCD1 TAB3 CLEC16A MARCKSL1 ADH1B AKR1C1 MIR369 MIR876 MIR146B SLC38A1 CDK5 CDKN1A IL32 ADAMTSL5 CAT HLA-B CRK TCF7L2 PRKCQ GBAP1 LINC01714 HSPA1B CD84 PSORS3 ADAM17 JAK1 SLC39A11 GCG CXCL5 LYZ RARRES1 TPM2 B3GNTL1 MFSD4B PIAS4 FABP4 PPP2R3C DAP ZMIZ1 SHARPIN CFB DEFB104A DDX58 AKT1 EBNA1BP2 TMEM258 CCR5 ANKRD30A IL17B FPR2 CD47 KCNH7 IFIH1 LILRA3 KRT10 CYP2B6 AZI2 NES SLC9A8 TXNRD3 POU5F1 PSMD7 STX1B BMI1 KLK1 PLB1 USO1 SLURP1 MIF GH1 NFKB1 AIM2 SIRT1 TNFRSF6B ZNRD2 ABCC1 FOSB ACD BCL3 COMMD3-BMI1 IL1B HAX1 REN MFAP1 MIR6731 S100A7 LINC01250 HPSE BMX H3P10 BTD FGF21 IL12B ST6GAL1 POMC IL9 DDX39B DEFB103B S100A7A FLG-AS1 NAA25 LCN2 SUMO4 AKR1C3 RNF19A RNF114 NFKBIA KLF13 ACTA2 FANCE ALB IL20RB SAA2 PTGS1 IFN1@ NOD2 F3 IL21 HTR2A EPAS1 NR1I2 S100A12 PRELP DLAT TREM1 NXPE1 PHB APOA1 HLA-C KPRP TIMP4 CX3CR1 CD69 FOXO3 LTA ITGAE IFNB1 GAPDH TLR2 LINC02210-CRHR1 FUT2 GPX1 P2RX7 WNK1 CDKN2A SERPINB3 KLK11 GNA12 LPAR1 IL18R1 MMP19 VNN2 ANKRD55 BCL10 GEM CCL3 AMOT GSTM2 ERN1 ATG16L1 JUN LCE3C NAT1 REG3A RBP1 CXCL16 BACH2 MMP10 CAST TREX1 EPHB2 UGT8 IVL WNT7B ATXN2L FLT4 NME1 ESR2 ACOT8 TRBV20OR9-2 TNFAIP8L2 PZP NDFIP1 APRT PRR9 NOTCH1 CD274 PGLYRP3 CP HNRNPA1 LOC102723407 ZNF816 CDKAL1 AKT3 NANS CRB1 SUOX IL1R1 RMI2 GORASP1 CYP24A1 KIR2DL5A FOSL1 GSTT1 MIB1 CD40LG SLC17A5 PIPOX SRF HIPK1 ICAM5 SYNGR1 CYP3A4 ITGAL UBBP4 PDE4A IL7 TP63 APP COMT SPHK2 LANCL1 FGF23 C1orf68 B3GNT2 FNBP1 CD1C LINC01932 PSMB9 TNNT1 CKAP4 GPR15 HMGB1 S100A9 CSF2 RPS6KB1 MMP9 ELOA-AS1 WLS PIK3IP1 ADIPOQ |

| **Table 2** A total of 10084 known therapeutic targets for hepatitis C were collected form the GeneCard database. |
| --- |
| FSTL1 CREB3L1 RPS11 MMP2 SLMAP TMEM216 ABRA SMARCD3 RALYL ERCC5 BBOX1 KLHL13 GCSH XDH EDN1 NAPRT CLK4 DECR1 CXCR4 GGT3P SALL1 IRAK1 RPS18 MSBP1 SLC10A7 CFHR5 PXYLP1 MIR20A LMAN1 CXCL3 MIRLET7G piR-39858-549 SUMO1 GP1BA TMC4 UQCR11 DDB1 STYK1 MYO9B MMP7 EP300 XAB2 UTY ZBTB12 CENPQ DTNBP1 ZEB1 ZG16 CDK14 ERG IGKV2-26 FAT4 PARN SOD2 ENSG00000285558 PNPT1 STK16 ARID3C SEMA4D AGPAT1 NOSTRIN MIR1246 NINL MAP3K3 MIR489 SFTA2 MAX OSBPL8 SERPINA12 NR4A2 PIR IPO9 LONP1 LOC102723566 PCARE EVC CXCL13 MIR200B SCYL3 SORL1 FIBCD1 CHD1 NAT10 SYN3 LINC00210 SLC22A2 SERPINF1 HEIH RPS6KA5 FAHD1 DHRS11 STK25 SLC12A4 BRCA1 CDC25B MAP1LC3C ENSG00000234273 MIR218-1 SLCO2B1 ABCD4 MIR331 BBS12 SLC25A46 TEK TERF2IP CD160 PHF20 COL1A1 GTPBP1 HNF1A-AS1 AP1S2 RAB33B CA11 NUDT1 ACBD3 ZNF83 PRDM2 SEL1L3 PDCD4 CEP350 FOXP3 SMYD3 MIR1228 RF00017-1272 NUP42 SELL LGI2 RPL23AP56 WDR45 SHROOM1 LTBP4 PYY BECN2 SIRT6 CCL19 PPIL1 GPR55 ABR FOXE1 C2CD3 CD38 NEFH RMND5B CFAP157 ROR2 FOXM1 FAM174A MAOA BPIFB2 GYPC H2BC21 RFXAP CDK8 EMC1 MIR181B2 HDAC7 ALG2 PLEKHG4 PPP1R3D GRB2 SYT13 EPB42 DUS3L EIF1 CETN3 CDK1 POLR2B RPS28 GDF10 HCCAT5 COQ9 TRAPPC9 P2RX3 MIR101-2 PLXNA1 HS2ST1 ENSG00000202542 CD82 PMM1 CBLL1 IL1F10 SETDB1 MIR193A GCLM PPM1A CPEB3 INTS1 MMP16 CAMTA1 DRD3 GMPPA AIPL1 PTP4A2 IL24 MLF1 CHFR TAF12 ID1 NDUFS4 COL18A1 PIK3CB COX8A CDCA4 EMX2 FUS TF CLCN4 MMP15 CELA1 DNAH11 NPR1 ASS1 USP42 TMEM59 POLR2J PLAAT2 UQCRC1 SNX6 ADAM23 POLG TFR2 RICTOR GRXCR2 RAG1 POMT1 IDO2 HLA-J LRRC8A CYP1A1 GNA11 ARSH FSIP1 CAPN1 DDHD2 IGSF11 CHST6 UNC13B TTLL4 PPBP HTR3A SDC2 ANKRD49 COX7A2 TDRG1 CACNB1 QKI KDM6A SLX1A MAN2B1 ACADL OFD1 PDP1 AHCYL2 CGA KDM4B PEX16 GNG12 HABP4 MIR885 CHRM1 CKS1BP2 CDC14C UFL1 FUT4 BAGE4 CD53 DBH ARAP1 AGPS SPACA6 WASHC5 PCCB UNG MRM3 COMMD5 GSTP1 LTBR SLC9A3R1 GNB4 TRC-GCA24-1 SLC9A3 OSBP PRR5 DOCK11 LILRA6 FKBP4 ADRA1A ASAH1 MIR122 ZNF350 GIPR MIR206 AHI1 GORAB NCR2 DEFB125 FAM221B KRT15 IL18BP GFI1 SCN11A FHL1 SMC6 GATA1 PSMD5 RPL4 MAD2L1 PRPF3 E4F1 PXN-AS1 CHMP2B RPS21 IRAG2 ACO2 XCR1 UBE2N WAPL IGSF8 FOXCUT KLF4 ACAD11 LMO4 KLKB1 LOC117204000 SCHIP1 HDAC3 HP BTK KRCC1 CLCA1 C1orf112 KIF19 HID1 GOT2 UIMC1 FXYD2 ATP7B RAB38 POLD2 DCX ACOT2 KIN COX6CP18 PPARGC1A LGALS7B KCNK10 FSTL5 EXOC1 ETFA AHRR PDAP1 POLRMT ZAN MIA2 MANF KLHL6 ENTPD7 CYP3A5 CUL4B CENPJ PITX1 PDGFRB IL17D RDX CELSR3 DNAJC9 JUND EHBP1L1 FETUB DCTN1 MT1DP LIN28B TNKS2 MRTO4 TTC3 C1QBP MUC2 NDUFB8 IL23A PSTK NDUFAF8 C5 PAQR3 ANKRD26 CMA1 PTCRA MTRNR2L8 HCRTR1 FST VWCE PREX2 LDLR ASTN2 CXCR2P1 TAB2 DDI2 METTL7B UCN2 KIF4A FAM161A LOC730098 PGM1 POLR3G CPD DCT HLA-DOA TANK DNAJB4 UGT1A8 PECR MIR16-2 TACR2 NUP85 CD177 LGR5 PIGG JAK2 TSHZ1 RAB5A HERC6 CASP1 LRP1B XAF1 PLA2G4C ABCG8 DAND5 OTUD7A AQP11 APOA2 KALRN IFNAR1 LAMTOR5 TALDO1 RAB11FIP4 B2M SQLE GLIPR2 EIF5A NEGR1 YTHDC2 NCF1 SPG7 ZNF614 HOXA5 NUP133 MIR221 XXYLT1 INPP5B SNTA1 PPID TRIM56 IL20RA GCM1 UBE2J1 HNRNPK TCFL5 TLR5 ENSG00000250839 CASC9 UTS2R SAFB2 NCK2 OXA1L DNAJC25 KLRG1 ZSCAN12 PSMD12 CLEC4M HSCB CLDN10 TAC1 GYPA TRAIP MEX3D KIAA0232 SEMA3B-AS1 ERCC8 NFAT5 GPX4 FBXO3 CCAR2 MSS51 DVL1 PRECSIT LRRC37A16P CMKLR1 HBA-LCR TYMS PEF1 ALG9 ZFAS1 SDK2 KCNK7 PTP4A3 CALCOCO2 SNCA ESRRB SLC48A1 ARHGAP26 PRCD KLRC4 HRAS ARSK NDUFAF1 MAP2K4 WSB1 CDKN2B ZNF480 MAP3K6 PROS1 PSMB8 MAS1 SLC16A11 PPP6R2 APOL1 RNF175 AIF1L ZBTB7C AHCTF1 ITGA2 MME SHANK3 TUFM THEG KCTD2 ABHD16B WDR26 SGSM3 NR4A3 ARSA MBL1P TOMM22 SOCS3 CCNA2 C19orf44 CGRRF1 RUVBL2 MCAT PIANP ARID1B PEAK3 ISCA1 ENSG00000255310 FALEC MT-ND6 WDPCP MAGI1 GBX2 SLC35A1 GAL SLC14A2 SLC30A2 RDH11 TSTD1 AUH EIF3H KIF1B MBD2 PIGT PSRC1 PLA2G10 ALAS1 FOXO1 DUSP10 CRLF3 CLCNKB PSMA5 FARS2 lnc-ZDHHC18-1 PIAS1 NPHP3 FBXO7 TMEM134 TSEN54 CFAP20 LRRFIP1 CNOT2 ALDH5A1 SERPINA4 FKTN CRTAP TK2 BLVRB STMN1 GUCA2A GALNT10 DPP6 PRELID2 MMP24 CKS2 SFTPA1 RHO XKR3 BANF1 CR1 RPS6KA2 DNAJB1P1 HMBS ACER3 SLC25A15 GADD45B MIR152 TDP1 LOC110599591 RFK ZFYVE9 TCTN3 DNAJB11 RF00001-212 GBA2 FBRS HSD17B13 NDUFB3 FPR1 FMNL1 CHTOP PTCD2 GNLY SMOC2 USP38 NDE1 ARHGDIB ITGA10 ANXA2 HORMAD2 HLCS SCARNA22 FH TBC1D24 LGALS2 C4B DGCR8 H2BC12 GSR NEU2 PAQR7 CACNA1C PDCD6 HCG23 AMPH UCKL1 ASB4 ALDH1L1 HNRNPH1 CCL23 CFAP47 SLC23A1 RBM4B LINC01554 PPP1R15A CD1B FCGR2A LINC01139 MDC1 MAN1A1 RBMY1A1 PRKN KRT9 NKAIN1 HTR1A ATP11B IFNE ALOX12 RB1CC1 NEIL2 EIF4E SIGLEC15 ABHD5 EXOC4 TMBIM1 CFAP36 MSBP2 EXOC3L4 F2RL2 ZDHHC1 LRRC32 SLC29A4 BATF2 BGLAP SLC35F5 URGCP TRAF6 AXL LMNB1 CKMT1B PNPLA3 CD276 KRT80 IFIT1B CSNK1E KCNQ4 PIK3C2B C6orf89 TUBB2B USP15 RNF31 TCEAL2 PTGER2 UBN1 SLC31A1 IFT172 CTNNA1 PKN3 COMMD1 FOXN3 NTRK2 MFGE8 IL10 PRMT5 DCAF4 LCLAT1 TSKU PDLIM1 KRTCAP3 RNF126 WDFY3 CEP63 CLIC4 GPSM1 DPM1 ACR EFL1 ALOX5AP LRP1 ANKH TNK1 RIPK3 RAET1L RFT1 APOC1P1 POLR1B OLFM4 TACC1 SAMHD1 ATP13A2 KRT4 PAX6 SCG2 PADI4 FMO3 NLK CAPNS1 MDM2 KCNK9 SNHG15 ITGBL1 TAP2 OR2C1 EFHD2 NBEA CLDN3 GNAI1 AKT2 EGFR VPS4B SLC25A4 CYB5B NDRG1 GBE1 ZNF285 MRPL46 MAPKAPK3 IFNL3P1 NCAM2 PRKD2 MIR130A MIR154 ZFP36L1 CYSLTR1 TMX3 MIR1269A GPR161 ERFE RNF146 CFAP54 CCNB2 ENSG00000271901 ERBB2 CREB1 NEO1 DDX3Y RPS3A MXRA7 LGALS3 CFL2 LINC01501 UPB1 LAT DHX38 NECTIN4 CLDN14 ZSWIM9 BTBD1 TARS2 ABCF1 ZHX2 CD28 SMARCA4 LRP8 MASP2 GPATCH3 ZNRF3 AGL NFE2L3 TTL ENPP7 STAT5B ENPEP SSB SH3D21 MIR93 SCT SLC22A13 TIMM23 SKI OAS2 TRIM11 GLIS3 C1RL-AS1 ATXN7L3 FAIM DBNDD1 SP140 G6PD NUDC DNAJC5B SCARA3 TACSTD2 EIF3J RF00017-5469 PPP2R2A BCAT2 C5AR2 MIR199B F10 COMP RTEL1-TNFRSF6B SCLT1 TAL1 ACSL1 ABCC5 H2AX TCAP ABL1 NCOR1 PRDM10 CMTR1 TXNRD1 SRGAP1 UBE2H BRDT VIP CALCR CCNE2 SLC6A8 LILRA1 MC2R MBTD1 NRDC PPP1CB MAP3K12 PAAF1 BARHL1 CCT3 SLC3A2 PIWIL2 RAD50 SYNE1 MBTPS2 MTHFD1L MVK RBMY1E NCL PSMD10 HRH1 piR-31470-425 HCG18 RNPEP NUDT16L1 PRKRIP1 ADH4 GRPR ZBTB10 LINC01018 HTRA1 SPTBN2 BHLHE40 RNF111 SLC26A2 ATP2B4 PTH CHST12 TARDBP STAU1 CHMP1A DDX21 ZNF160 B3GALT6 H4C4 HDAC11 PEDS1 NXNL1 MTO1 HADHB ADAR MPIG6B PLXNB2 ARMS2 GAS2 ADIRF EN2 ENSG00000235099 NUMB TNIP1 TM9SF2 INHBE RPL19P8 PSKH1 NSFL1C HBB HEY1 KNG1 ITGAX CASR XRN1 CANT1 LIN9 IRF2 GNG7 PGR RUNX2 MT-CO1 GPR84 SOX1 FCRL5 ZNF99 FGF10 MBNL3 ODC1 SDC3 ADORA2B TMEM218 SEPTIN3 SYNJ2BP POLE E2F2 THRA MAPK11 TBC1D22A ENO3 COL4A6 MIR22 IRGC NAB1 TRMT10A TNFRSF18 TRPM3 MAFIP RAP2A FECH PLG CRIP1 ASB14 AOC4P AZIN1 SLC7A7 LAMTOR4 IL10RA IL36RN POLR2J3 CDC42EP4 ARG2 ASH2L PANK2 TSLP DNAH8 ICAM3 IFNA14 TAF8 SLC1A3 RNF139 RF00017-1304 ADAM8 SFTPC BCL7B NUDT13 MT1F C1orf53 RPL35A CHP1 LY6G5B RAPGEF5 SAA4 POTEI RPL29P19 MAP4K2 ACAP2 SULF1 ELOVL1 PPP4R2 KCNV1 ACP1 PTGFRN SIGLEC5 ZMYM2 SLC2A4RG LOC645752 LPL CA5B CEACAM19 SH2D4B GET3 TGM4 SLC6A12 CD84 RASA1 PHKG1 MAGEA11 NONO RARRES1 CLEC5A B3GNTL1 ITCH MGAT3 TAT-AS1 KLHL26 MBP GJC1 C20orf194 EXO1 SLC2A2 HRC LRG1 PIK3R1 MAF HELZ ADCY4 SLC46A3 ADRB3 MPEG1 PTRH2 DMD MIR181C NDUFA12 RF00017-4974 ADCY2 lnc-OLFM1-1 SRM OTC MIR199A1 ABCB11 OTULIN KCNC2 SSH3 CHURC1 CCBE1 SARNP PER1 TIMM50 SELENON KLC2 HDAC1 SMARCAD1 OIP5-AS1 IATPR FNDC3B MTCP1 VMA21 BLZF1 SHOX ZNRD2 CAV2 AREL1 piR-37567-196 MED26 BCL6B DUS1L CHRM3 NEK9 DDO WARS2 MARCO DCHS1 TOMM40 TPPP C8orf49 ADNP GSTM5 BTD MBOAT7 PDCD1LG2 MIR196A2 APBA3 APOA4 ZNF689 PMS1 EIF3E IL9 RPL31 SLC25A51 MKX KDELR2 TFCP2 LY9 NLRP12 TLK2 CTSO RPN2 MARS2 SDF2L1 HIBADH ENSG00000288577 IGFBP3 DEK PSPC1 ARHGAP24 SORBS3 C4BPA ALB JPH1 AGBL2 C1QA TERF1 KIF22 GSE1 EIF3CL ACTBL2 NINJ1 COL11A1 SFXN4 UCP3 TREM1 HLA-C SPRED2 PLA2G12A BBIP1 ELOB TIMP4 HAUS3 GBP5 PITPNC1 SLIT2 RSF1 MYLK3 CCP110 C2orf88 CACNG6 CEP128 NKX2-2 WDR83 SNORA71C FUT2 CGB8 GDF7 FZD4 GALNS STX6 CIDEB CASP4 KCNJ11 TPR BRD3 LYRM7 SLC45A1 SERPINA3 HLA-DRB3 SNORA73B STAB2 NATP RPL35AP C9orf92 IRF9 MCPH1 SLC26A7 FABP2 FBXL4 TRG MINAR1 SYNM BMP2K NFU1 POMGNT2 HINT1 GLP2R H2BC3 WNT7B CHCHD2 SLC4A4 BAIAP2-DT RPL38 RPS16 CSNK2A1 MAP7D1 FKBP6 TWIST1 TRAM1L1 EPO NTN3 AOX1 JRKL DTNA TMOD3 LY96 EEA1 KLK3 IL1R1 KTN1 KIR2DL5A MAB21L1 CCT6A PHKB EHHADH HEPACAM KMT2C LAPTM4B LCN8 ITGB5 RGL4 FKBP2 TYMP GGT5 C9orf62 NR1D1 TRIM5 APOC1 RNASE4 NEK2 ARID1A SIGLECL1 RRAGB RCN2 SHMT2 MLYCD UQCRH AP1S1 SDHA SMAD6 ZNF251 THORLNC YPEL2 RBMS1 COLQ HNRNPUL2-BSCL2 UGT2B28 DRG2 KLRB1 DNASE2B RPL23AP1 NLRC3 SAFB LOC108961161 RFX2 MAPK8IP1 ST6GALNAC1 FANCD2 ARL6 MEF2C LBR IL2RG COG7 HLA-U MYB KLF3 PLXNA3 KCTD14 FZR1 XRCC2 MMS19 ESPL1 GMPR2 ENSG00000230941 CHPT1 RPTOR ERBB4 TSPAN32 MAP4 ACTR3 UGCG LOC111216273 ZAP70 SLC6A2 LPP PTPRCAP HIVEP1 IL12RB1 DFFB EIF4EBP2 ATRX DMRT1 CHST8 MIR31 ARV1 IER2 MIR26B NMB PCNA-AS1 COX5A EIF6 AIFM1 GSTA1 POLR2L MIR146A TRAF3IP2 LILRA4 LAMA2 PXT1 RLBP1 lnc-ARHGEF10L-1 APOBEC3D MALT1 ARVCF MTCO2P1 MEX3B NID1 C1QC KIF7 MIR451A MMP13 C8A MIR33A RTP3 ZBED3 KDM4D SLC15A5 METTL23 SLC25A37 CCDC12 HCP5 CLHC1 AMBRA1 UCN piR-56399-061 USF2 FHL3 MMRN2 RAD51B DSP NDST1 IRF2BP2 ZNF230 MBL2 RNF41 THOC5 MSN SLC9A5 MUSK SMARCD1 SLX1B ZNF347 FKBP7 STRADB DDOST GPNMB TTF2 SFPQ KCNT1 SLC39A14 AKR1B10 RGS16 RPS20P33 RARS1 MECP2 PSMA1 BCO2 NFATC4 CSN3 NOS1 BMPR1A HLA-H PRL FUBP3 H2BC13 ACACA ABCG1 ATOH1 CREB3L3 MIR149 SNORD42B LARS1 CD55 LYPLA1 RPS23 ZNF890P SSX2 NUP93 LNPEP SMAD3 YARS2 TBC1D12 RMC1 PTGDR DDX27 COQ7 RERG NEDD4 THAP11 NEK4 KRT13 MIR148A PRKAR2A ATP6V0B MIR204 CACNA1E SKP1 PEX14 PAFAH1B1 MIR19A OSTF1 H2AC18 FYCO1 FBLIM1 RAPH1 PLTP CTIF lnc-HLA-DRB1-7 SLC25A1 DOCK8 EZH2 GRB7 HLA-S LINC00941 RNASEK PELO MIR127 PON1 ATP4A HBEGF lnc-ARG1-2 PI4KA POU2AF1 MRPL12 LINC00222 MIR192 ZC3H3 ENSG00000253170 DDAH1 MIR223 ACBD5 MGAT1 CCDC137 TIPARP UGT2B15 PGAP2 DNASE1L3 CHEK1 CKB LOC110599571 RTN3 FZD2 PSMB6 MAP3K14 PHC3 PPP1R8 TNFRSF11B GOLPH3 EXO5 PIBF1 PDE6D NKRF CLK2 MIR10A CXCL1P1 ZPBP2 DUSP2 TNFSF15 SECISBP2 SPRTN C9orf50 URI1 GNG10 PIK3C3 DHDDS OTULINL CTSW NEFM TNNC2 ANKRD28 MRPL28 RAB14 GRK3 PIGV CXCR2 APBB1 ARHGAP45 B4GALT1 BCAP31 CLDN22 MIR181B1 CD40 FAT2 lnc-FOXC1-2 HEPN1 PIH1D1 RAET1G EFTUD2 HSPB11 MIR23A CS ZNF318 IGFBP7 SCN4A SIRT4 ARHGAP4 FAM135A EHMT2 NDUFAF6 MIR1271 EXOSC10 WTIP COA5 GTPBP3 PACS2 SCD5 LSG1 MAL SNU13 ENSG00000225116 AQP4 ARMC8 RD3 GRIN2C SLC47A1 DMPK MTRNR2L6 KIFC1 SLC7A14 POC5 LHX5 SMC1A LPXN TRPC7 IDNK SERPINA1 RPS13 CRHR2 PRKAA1 FADS2 KIR3DL2 C3orf33 LIPH ATP2C1 SYNE4 CYP4F3 RCC2 RNF103 AHCY CROT MIR574 ALG12 EGR1 CCL17 KHK ESRRG SLC12A2 RDH5 OTX1 CD58 PTGFR IFNL3 STOX2 SELE EXOSC9 BPIFC PSMC6 LNCRNA-ATB PROKR1 KLRC2 ABCB1 FAR2 SSR3 MSI1 PPP4R3B TRAT1 PLAGL1 USP54 MTF1 KLF6 THBD ABCD2 ITGB7 DNAJC7 PRH1 CHKB SCUBE1 TM2D3 CLEC4C HUWE1 DCD CDH17 CD180 FAAH IL17A TMPO TM7SF2 GNAT3 FLOT2 OXCT1 H1-3 LTBP2 ADGRL1 CYP2A6 ABCA8 NDUFS2 UBR5 BTBD16 GP6 LEF1 KCNQ1OT1 MAPK14 SERPINB2 GTF2F2 WDR17 PLA1A CAMKK1 HPR NHP2 CLCN2 ANXA6 RRP36 DPM3 H1-5 SLC25A13 KRT24 SMPD1 TH ZC3H12A CNTF MAP3K8 NCCRP1 STC1 EFCAB14 STOM ENSG00000225867 CPSF3 CWF19L1 ENSG00000199633 MYH3 HOGA1 SULT1A1 YY1AP1 LOC109286563 PLA2G4A MAPRE3 MIR100HG GGPS1 EXOC3L2 MAP2K2 RPS9P3 ELN MPI HHCM TLR8 LNP1 ZEB1-AS1 ABCC3 RRAGA KCNK17 HLA-DMB CARS2 RHAG UNK HLA-A PWWP3B RSPO4 EDNRB LDHA FAM167A SCD ARMH3 BAGE2 MIR106B ZNF875 XKR5 HBB-LCR SHANK1 CD226 RBM14-RBM4 TCTN1 STAT3 PPP2R5C SYNJ2 CAPG MBD4 SLAMF8 ST3GAL6 ATN1 ROBO1 MPZL1 ARSB TDH TSSK3 MAD1L1 TPH1 DDX52 AMPD1 NOP16 TSPAN16 ANKRD11 NPAS4 PCNX4 EIF5A2 EGFLAM GUCY2D TMEM238L FGF4 CPM UOX CIDEA SLC26A4 IER5L N6AMT1 DGKB RF00017-5475 DPYS MTCH1 WDR4 SLIRP HK1 LOC106096416 VPS33B HEXB MIR24-1 TNKS SLC35G1 CRLF1 TICRR WAS MADD LOC110599585 KLF12 ARHGEF18 KCNK1 ARRB1 HSPA1A DAPK2 EREG MIR30C2 IL17RB CENPA MCAM AQR IPMK MINDY3 CAMP GRHL3 GABRA2 ADAMTSL2 TNFSF18 PPIB CALY CCDC127 SFN GOLGA5 UGT1A4 SSTR3 MAGEA5 RBL2 PYGO2 ENSG00000247679 PPP1R10 CSE1L ASRGL1 LCA5 TOM1 OR5AN1 PTX3 ANG UGT1A6 ENSG00000259605 ATG13 CINP piR-57133-394 U2AF1 CASC2 VCX PFKM SGMS1 DKC1 CREBZF PRPSAP1 PPP1R12C TTC7A UBD S100A1 RPL6 NAGS ELP3 GPAT2 MIGA1 C8orf34 PELI1 PPT1 SLC35C2 MT1X UBE2B KEAP1 MUC4 MST1 OMA1 BCL2L13 GNB2 JAZF1 MIR3928 NUP50 C7 KAT2A CCDC130 GLYCTK ENSG00000285806 MTUS1 UROS HARS1 TRAPPC10 LEMD3 PLEKHF2 CPQ HOXA7 POLQ HDL3 DIMT1 PYM1 DYNLRB2 SOAT1 MR1 FFAR1 HSFX2 MT-ND1 F8 ACHE TNFRSF12A EGR3 ENSG00000250899 ZNF184 SERPIND1 OASL IFRD1 PRR14 KCTD9 ZNF267 LOC101929829 PPP1R3A PRR11 TXNIP KIRREL3-AS3 NUDT3 RAB4B-EGLN2 MIR187 IL27 ENSG00000228559 CHAD GABPA TLR3 CAMK2G ENSG00000145063 FGF14 HMGB2 TRPM5 POLB PTAR1 MARCHF2 KIF3B MTMR9 MIR758 SEC31A REELD1 GLRA3 UGT2A1 TOR1B LSS HNRNPC TMEM100 CLDN19 DHRS9 ISG15 ZCCHC14 FXR1 ZMYM3 TGDS CASP3 KCNQ1DN SCLY CCRL2 ENTPD5 MT-TE PDIA5 SUCLG1 TMEM67 KCTD20 KLRF1 PRPF40A AGO1 CLDN12 NPY5R IRF4 MIR449A PRKCE ITGAM TUBE1 SKA3 RGS2 PDPN P3H4 COX7A2P2 MST1R MIR423 MGAM COL3A1 MOB3A PTMA ZNF602P NAPA GFM2 LIPF IQCN LOC110386951 ASGR2 C1QTNF4 IVNS1ABP MIR125B1 SIGIRR DUSP19 NDUFV3 PPP1R2 DNAJC14 ACSS1 FOSL2 TUBA4A IL1RAPL2 EPPK1 EEF2 SPAG17 PRDX3 EPHA3 HNF1A RPL7P51 P4HA2 MOV10 RFWD3 TMEM150C SNHG20 B3GALT4 SERGEF OSCAR ATP5F1E NPFF PECAM1 DEDD PTPN1 PEX2 AOPEP MYH14 LOC110806263 CCR8 GADD45G SRARP ENSG00000201547 IFITM3 SLU7 CC2D2A GPATCH1 PHACTR2-AS1 LHCGR SAP130 UBE2S SNORD35A ANAPC11 CNR2 CD200R1L ATP6V1B2 PLCG2 MAGEA2B CAPZA1 CDYL2 GLRX3 HRH3 CRYGD HTR2B CCR1 PGRMC1 UVRAG NUS1 CLDN5 ITGA2B PSAP MYOM2 NCS1 CHM CYP26C1 CCNL1 LYN TGFB3 ELK1 HGD SERPINE2 MYO3A TCP10L MIR1231 ADAM33 MAPK6 COX6B1 DNASE2 MSH5 TCN1 NTMT1 RNFT1 PYGL NUCB2 SNX27 FOXO4 NUBPL PLAAT3 ID4 NOD1 CDH22 INA MCOLN1 PERM1 RIN3 THY1-AS1 PSG2 HIBCH PLA2G5 MED17 DIP2B PSORS1C1 LPA PIGA COL4A4 LY75 TPP1 UTS2 RREB1 MIR92A2 NQO1 MIR1207 SP1 TOX4 ENSG00000225544 SEC24C GTF2A1L HSPA9 APOBEC1 AXIN2 SURF1 GTF2F1 SRRM3 RPS15A TAF1 MYO15A GIMAP7 AP1G2 ATP6V1E2 MT4 CKLF H4C6 PLEKHN1 MBNL1 NUP160 USP21 SERPINB1 FGA IGFBP1 SLC1A1 ACSM4 PSAT1 ENSG00000287596 MOK PNPLA5 TBR1 IFNG FOS IQGAP1 CYP2D7 ZNF226 TGM6 EMD RETN CCL8 CCDC82 PUS7 HCG4B TP53INP1 TUBB6 ZNF300 FOXL2 LATS1 HTR6 MIR26A2 SPOCK2 HTATIP2 FGF2 PRODH DDX6 NTN1 DPH2 VPS28 FCGR2C TRAF4 CORO1A ZNF460 RRM2 C19orf12 COG5 MRE11 COPS8 AKAP9 NGLY1 MT-ND3 HNRNPAB GPSM3 ADGRL3 MIR429 SLC20A1 ADAM1A SIL1 ASB6 MRPS23 SPTB EFEMP2 EFNB2 LOH12CR2 PCM1 FMO5 TSG101 ATRIP CEBPG GTF3C4 TNFRSF1B CLEC1A LAMA5-AS1 IQSEC1 AEBP1 RBM22 MIR509-3 IGKV2D-28 ANKRD2 SHROOM3 CCL28 JAM2 SNRPN ALX4 TRIM38 CACNA1S CORIN CD300LB COQ8A PLEC SNHG17 SMG5 VWA7 ENSG00000234406 NCAPD2 SLC25A6 WNT2B DCP2 ASNS GLYR1 RPL18 ISG20 CASZ1 POLR1D LOC110596866 GJD2 MIR92B C1D CXCL1 LINC00926 EPM2A LDHC PC DEFB127 FAH UBE4B TMEM116 PAX8 IRF1-AS1 NR2F1-AS1 SLC2A4 SLC26A9 DUT LY6H LRRC59 MAP2 EDEM1 NOS3 ADH1A RF00017-6523 CYP11B1 ANGPTL6 FCRL4 GSTM3 KLF14 EIF3L KCNA5 PMS2P5 SERAC1 WRN NFATC2 DZIP3 TNRC6C RIT1 RAB13 FBXW11 ETAA1 AKT1S1 PHF14 NOS1AP FAM3A TMX1 PRDX2 PRMT3 BCDIN3D CDKN1C HSPH1 AQP1 COL17A1 GFAP KIF24 UBA2 TMEM92-AS1 PRSS16 SUGP2 PCDH9 PI4KAP2 ADAMTS13 MIRLET7D IGFL3 GBP3 MIR324 RARG RANBP10 MYO7A ACVR1B CD96 DENND1C RBMS3 FAAP100 PSMD1 C7orf31 ILVBL RNF186 IFNGR1 SLC29A2 MATN2 GNRHR2 FGL1 PSORS1C2 TGFBR2 ORAI2 CDC123 ACMSD IL33 MSLN MT-TK DEFA6 WWTR1 DDX60L COBL CIB1 CLDN20 HLA-DRB1 CTNNAL1 TLE6 MIR325 TNFAIP2 FOXN1 HERC4 DRAP1 PEMT SLC38A1 MCM3AP KCTD7 MYO1F RNVU1-18 ST3GAL3 RAB11A PTBP2 CLEC18A OGA SETD7 PARP3 RBM8A VANGL1 NDUFA3 FABP4 KCNB1 CANX ENSG00000272279 SLC25A28 SHARPIN ADIPOR2 UGP2 CIRBP SEC14L4 FRZB PABPC1 MIRLET7A3 APOBEC3G CLDN8 VPS52 VEGFD ZFHX3 PCSK5 TMEM126B GIMAP5 NDUFA9 SCRIB AURKC ORM2 EGLN3 GUSBP2 PITX3 CUX1 UNC119 GPX3 GPM6B EIF2S2 CKAP5 UCHL5 AIM2 LOC105369388 PNO1 ANK3 LOC117600004 KRT5 SIX1 TIMP2 MIR371A MIR345 QRSL1 SHC4 CCL15 CCDC22 MORC2 TNXA MT1H MIR572 FOXD1 COX4I1 DSN1 AMT CBR3-AS1 CTAGE1 HSD11B1 KITLG HDGF KRT6B SETX DDR2 KCTD12 WWP2 CTSH PAF1 SPATC1L TRMT10C IFT57 ENSG00000246541 PRSS36 TNFSF9 DCTD CFP MAP4K4 MFNG DOCK3 ALDH3B1 STX16 FEZ2 THSD4 DLAT KCNJ5 CASTOR3 CDC5L LMNA TBCD CASQ2 PEG10 PRAME MIR431 SRSF5 SIGLEC7 PANK4 RAET1E WNK1 GSDMD MIR500A RECQL4 PPA1 DPAGT1 HAS1 MAGED2 ANKRD55 ACOX2 ATCAY DOK7 TFPI2 GTF2H3 ATG16L1 LHX2 PDHB ERN1 TRA ADAMTS5 PTH1R NOTCH3 NF2 KRBOX4 ZKSCAN7 PHYHD1 FBXO32 CLPP MTMR3 TXNDC17 PAM16 TRNP1 ATP1B3 TRIM25 SDCBP2 HOXB2 CRKL ENSG00000228363 GATM NUP62 PGD CDKN3 CDH4 MPG GAD1 ITPRIP SERPINB12 H2BC15 DHX36 TNFAIP8L2 TFF3 NDFIP1 TNR APPL1 WAC ADCY9 WHRN SULT1C4 PTPN13 SLC22A9 TAS2R20 SSTR5 CDKAL1 SFRP1 SUOX EIF3G ARHGAP22 CASC11 ULK4P2 MIB1 H2AC20 NPC1 ALDH9A1 SUSD1 ZNF678 ICAM5 ADD2 RASSF1 PKD2L2 CDKL1 SLC27A5 SNORA99 CLIC6 PRODH2 IL7 HCAR1 MACROH2A2 HOTTIP PAK2 ATP2B1 GATA4 ELMOD1 PRKAG1 MS4A14 EEF1G SMAD5 INCENP IGF2BP1 FCHSD1 HLX MDM4 UBE2J2 MIR216A GNL2 VARS1 CDX1 ZNF200 PFDN2 FBLN5 HBA2 MIR490 PUM1 KLHL24 CCDC88A RAB7A PMS2 ERVH48-1 CBR1 ITPR3 BIRC3 NRSN2 LIPT2 ZNHIT6 SPOCK1 DCAF8 ZNF638 APBA1 RXRB ACTN4 PLCE1 INSL3 EIF2B5 DLG1 VPS33A PINK1 FIBIN GRAP2 PSTPIP1 MTCO3P22 CYP26A1 APOL2 TACC2 CNBP ENSG00000258711 HSPB1 INPP5E SNHG16 ARRB2 U2SURP RMRP SLC7A6 CUBN H3-3A IGF1 G3BP1 HSD17B10 NUP155 CYP39A1 POLK GDF15 CYCS LPO TPRG1L GZMM PAIP1 DDX11 C18orf12 CACNA2D1 IMP4 PROP1 UBB PON3 RPA4 NDUFS1 SLC38A5 SEC1P ARRDC2 MIR15A PPP1R13B ARL2 TOP3A NDUFAF4 UGT2B17 FGFR1 CEACAM6 MIR545 FXYD6 CERK CLCNKA MAGEA2 HLA-DRB5 GOT1 BBC3 CASP6 UNC5A HOXA1 ZNF181 MRPL38 PIKFYVE UGT1A1 PI4KB B3GAT1 SUSD2 NOC4L FADS1 MIR6124 CSF3R POLR3K RHOT1 OXT GPR153 RIOK1 HERC2 HSD11B2 ZNF48 CACTIN USE1 HNMT MIR129-2 HCST ACTR10 NASP CYP2C19 COL9A2 PALLD TBL3 MT-ATP6 TLR1 CPNE5 ZIC2 TMEM240 MAP3K20 KPNA2 MIR374A PARP16 A1CF PDIA3 MACROD1 DCP1A NECTIN2 CCN4 CLN5 BIRC5 IFNL1 PRR16 CES2 NIBAN3 GNAS DDX53 MRPL52 GHR CCDC124 TRA2B CLEC4E MGAT4B BBS7 GLE1 SERPINB9 GTF2H2 KNTC1 MYL12A CCDC57 FGF17 LAIR1 MIR151A IL17RC ITGB1BP2 RBPJ ALDH18A1 TPI1 DNAJC5 GATA6 CABP1 ALDH2 LOC110806306 IP6K1 EMP2 C3 SRSF6 MRAP KATNIP GJB1 PIN1 RIN2 MIR378A MIR99A APOC4 ATAD1 NELFE MRGPRD UBR3 DNM1 HYOU1 UGT2B10 ATOH8 USP7 ASCC1 HNRNPR CARMIL1 SERPING1 TPCN1 STARD13 ASIP SLC7A3 TNFRSF10A ADI1 CDH10 KL NPTN-IT1 SCP2 MAFK SON SH2B2 SBNO2 RACGAP1 SLC6A6 PSMD3 PPP1R9A RAB35 AVEN BCAM PCK2 C14orf132 FKRP HLA-DRA GUCY2C PEX11G DAO NLRP5 EDNRA ZNF331 HMGN4 EIF3M GHRL SLC17A3 ZNF24 MICU1 ESRRA ATPSCKMT FUCA2 PTMS IRF3 ENSG00000250993 BDH2 APOBEC2 PENK SMAD9 SLAMF1 MT3 ATG10 DLGAP4 LOC102723944 LIF OXTR ACTC1 SUV39H2 CNTFR EPX ENDOG WNT3 ZNF467 SLC25A21 IL5 KRT37 BAGE ENSG00000285245 PTGES OPRK1 CST3 WDR6 NUP88 CD300A TEN1 FCGBP VASH1 SAPCD2 DHX16 LEMD2 CLEC3B ARL2BP CRP CD99 MEG8 GLA MOXD2P HSPA8 CCDC18 PLA2R1 COG3 NGDN PNOC STK40 DPF2 YIPF7 TRPV5 UGT2B4 PKIB COLEC11 MED12 ABCC11 MGST1 CYP7A1 PHF1 AGTRAP RHOBTB2 ZNF432 SRD5A2 DCSTAMP FUBP1 IL27RA KIAA0586 IGLL1 MT-ND2 IL5RA SCARNA12 SMPD2 TNFSF12 ENSG00000268790 FN1 MTR DHFR ALPP PPP1R12A SHFL RSPO2 ZNF7 ACTG1P20 MLIP GPR132 TPMT TTC21B SERTAD2 ATP1A1 FRMPD3 MIR144 PLXNB3 UBQLN2 SORCS2 ACVR2A PRIM2 NR1I3 YWHAZ FAM83H GNAO1 OPA1 RPS6KA6 MMP23B PFKL HSBP1P2 AURKB LIPI SCARNA8 IFI16 CSTA DISC1 USP4 DZANK1 OSBPL10 CLTCL1 NUAK1 NPHP3-ACAD11 ALG6 NPPA LAMB3 CARD14 EGID-105180392 ANKRD20A1 CCL26 LCN1 ARHGAP42 MFSD4A NCOA3 MTHFD1 CCAT1 PLA2G6 TPT1 MRPS28 SEC63 TDRD10 MTRNR2L10 FAM66A MIR208B CEBPD ALDH6A1 G6PC2 GRWD1 WFDC21P BCKDK CTSB PFKFB1 RPL12P33 ZFP36 CMYA5 POC1A PXN TNF CYHR1 SIRT2 CTAG1A PHYKPL GANAB PDSS1 PGAM1 NGFR ATP8B4 MEIS1 BMP8A NEDD4L EDAR IL1A GNRH1 ERCC1 FAM3B CAV3 AQP8 CREBBP ITGB1 PARK7 DM1-AS BRINP3 COQ5 PSMC4 SGK1 NOP10 SELP IFT43 PSMD8 SOX10 RARS2 MAP1LC3B MEMO1 LRBA ZNF827 FNTB KRT8 AURKAIP1 UCA1 GAS5-AS1 DPP7 FHIT PPOX NSMF RHCE SENP8 ATG7 PLIN3 PANK1 SPP1 GLI1 SFTPD MCFD2 CASP14 EPS15 PLAC9 SH3BGRL2 ASPSCR1 GNS FDFT1 ENO2 CBX1 PEX26 PTGS2 BMP7 UBE2D3 LRP5 ARFGEF2 ARR3 REXO1 CD2 NEFL NELFCD HPGDS ENTPD2 NAV2 COX7B TRAPPC5 ANKRD17 ADH1C MIR183 ATXN7 IL21R HAS2 TGIF2 PRDM6 EPHX4 CYP2C9 DNMT3A FCAR MIR1204 piR-30188-078 RUNX1T1 IL4I1 CTSK HNF4G PSIP1 ZFP90 AP2B1 SLC30A7 SMCHD1 MIR658 S100B BMP2 ESR1 ZFPL1 SYT7 ARHGAP12 PGGT1B SLCO1B1 CCNDBP1 KCNN3 NACA SST PRF1 CDK4 TCF15 PRMT1 ITIH3 TNFRSF19 PIM3 KAT8 KCNAB1 SRRT RBM3 MXRA5 H1-10 GRM1 IBA57 RAPSN PDE4D ROPN1L RAMP3 DLEU2 ADGRE2 ITGB8 STK19B THEM4 FRS3 PDE3A TNFRSF10C RASGEF1A UBE2K XYLT2 HIPK3 TEX14 GAL3ST1 IL36G LOC111255642 DHCR7 PDZD7 FGFR4 CRAT SLC19A1 LIMS1 TRIM14 MFAP3L PIK3CD SLC7A11 FHL5 ACAN TINAGL1 ENSG00000224605 ACKR2 CWH43 SMIM23 SLC5A3 CHST2 SPATA2 ELOCP28 PGLYRP4 NTS CANDF1 BLOC1S5 RPS4Y1 TNFRSF8 TMEM259 FOXE3 PTGIR PRM1 ACAA2 FCMR S100A16 PLS3 RNPC3 DAG1 WWOX GTSE1 TMEM121B GP2 PLOD2 CHIT1 KLF9 RPS24 FIGN MRPL9 TSPAN2 PCSK2 RPL24 ZFAND6 NUF2 ARID2 RCC1 CD86 RGS18 CALM2 GYG2 LFNG PARS2 DYNC2I1 CTSF GNA13 TSPAN31 ARL8A FUT8 IBSP AGA B9D2 STAT1 PTTG1 COL8A1 CASP8AP2 MMP12 TNFSF13 GABRA1 PEX11B HABP2 DLL4 MYOCD POGLUT1 MICOS13 CD68 SMARCA1 APOF WEE1 GCDH SSR4 ENSG00000255422 SPAST RGS1 TEPSIN MIR338 INTS5 MIR384 BSG PARP8 TEAD4 MS4A3 FN3K DTX4 EFEMP1 TULP2 TNRC6B ARMC2 RERE TIMM8A PIGB AJAP1 ZNF608 TRIP11 MIR335 SYP GALNT15 FKBP8 POT1 KIAA1109 PTPRC CCT7 MMP11 EEF1A2 HEXD HACD1 XCL1 BPIFB4 RAB24 CARHSP1 LOC111242785 ZNF577 HDDC2 MIR134 SDS SHC2 MTUS2 CASQ1 MIR942 INPPL1 ASB11 CHGB IHH DIAPH3 GGH SNORD124 GGT1 ADARB1 VPS35 DBP WNT1 COL5A3 RHOD WDTC1 ZC4H2 COL4A2 HEBP1 SLC1A6 LUM C1S UBASH3B PAGE1 GLO1 SNORD144 CD302 RABIF ERAP2 SIRT7 PFAS PPIL3 MICD HNRNPD RPLP0 ENSG00000200090 MKNK2 U2AF2 CPLANE1 EFNA5 CCS FCGRT YWHAG ZNF555 IGF2BP2 CCR6 COL11A2 CNTLN CDC14B SYNE3 LDLRAP1 ANGPTL4 HHEX LGALS7 ACOT13 MIR493 INSM1 HDAC4 SYCP2L GLRA1 RPS6 ERVK-6 ZNF717 MIR1236 SPPL2A SFXN1 CTNNBIP1 SCGB1A1 HMGN2 CYP2S1 TTC36 LIAS MAPK1 MRM1 ENSG00000255122 ATP9A HSD17B3 SAG CTRC HNRNPA2B1 GADD45A FYB1 TMEM70 GNG3 FTO PPP3R1 ENSG00000272489 GNAZ DHX8 STIM2 SPI1 GYS2 UBE3B PLAT SF3A1 COPE KIAA2012-AS1 EIF3A COA4 GPRC5D LRRC23 KIF12 BLVRA SLC22A7 DAZAP2 CASC15 KDF1 IKBKB BOK THBS4 SDHAF2 DOK1 PGAP1 CXCR3 FSCN1 KIF17 CHI3L2 TRIM66 ENSG00000252824 CBR3 BNIP1 AQP3 KRT6C SIRPA IGFBP6 LYVE1 APOH ENSG00000273209 FLG2 ARHGEF15 NPHS2 SRD5A1 NDUFV1 STEAP1B RPSAP15 REXO4 CLEC7A HSPA14 SOGA3 ANKHD1 VPS37A GPRIN1 KYAT1 BAZ2B C1QTNF6 POU6F2 BRD7 MIR30D NISCH DEFA1 DTYMK ALDH16A1 TAX1BP3 F13B RPL34 MARK2 AKAP12 ENSG00000232080 KIRREL2 SLC25A27 KIR2DS2 HSD3B2 AMMECR1L ATF2 SLC39A13 QTRT1 TNKS1BP1 CCNT1 AP1B1 CD74 DYNLL1 PLSCR1 LIPG APOD CLDN25 DENND6A MAPK8 RBBP8 WNT11 SRP54 GPR35 NRGN SIGLEC12 IFNW1 ACVR1 STAR HLA-DQA2 RNASEH2B KLB TUBA1C TTI1 SH2D7 PIK3R2 SYTL2 LOC112529895 CYP3A43 AKR1B1 MTFP1 GRID2 ZNF423 PSMB4 XPNPEP3 CYBB SART1 SHB ENSG00000270060 CHST7 SEMA6D NDUFA1 HSPE1 MYCN KCNJ3 MAPK13 TBX18 PCDHGA8 RPS6KL1 NTF3 MT-TH DPP9 GSPT1 GTF2B IGHG1 GUSB GSDMB EXTL3 LIG1 MIR320B1 NID2 HYLS1 LSM8 TMEM97 SUZ12 KRT18 MRPS16 MACC1 FAM72A OR2F1 CDK5RAP3 YTHDF2 GGCX ARPC4 TCEA1 RNU4-1 SUSD4 EGLN2 KBTBD4 TNFRSF14 DEFB118 TRIM28 FGF5 IGHG3 NSMCE1 PTPRN TST RNF24 IFNLR1 CHCHD10 MIR25 TENT5B CELF2 CD70 ENSG00000201346 SRC SETD1B ZNF606 NFS1 SNRNP35 OSBPL9 NUP107 PLIN5 PDE4B SNORA64 ARFGAP3 IL1RN MIR296 MIR9-2 RAC3 MAFB TMEM14B GRINA ADIPOR1 TFPI DPEP1 MIR323B SLITRK2 FABP6 SMTN BBS5 NACC1 CYP17A1 TWIST2 SCG3 GFER CD209 DDX59 GJC2 DNTTIP2 ENSG00000236352 TNNI1 GABRB3 PCBD1 DDX1 SP3 FLRT3 NR2C1 SLC25A10 TBC1D7 H2AC7 PCYOX1L TNFRSF13B ENSG00000246477 VPS54 MKI67 PET117 GLS ENSG00000255899 LGALS13 IL4R CCL5 PEX5 CLSPN LOC109951029 EMILIN2 SMG7 MIR373 TLDC2 GINM1 DZIP1L VASP LOX MIR455 BAG5 COA8 BUD13 UNC80 TGFBR3 MIR4709 CCN1 MED24 CASP9 PDCD1 MAP3K7 AKR1C1 SPSB4 CARS1 ZNF32 BRD2 IL32 LAMP2 CAT SLC7A1 MIR365A PRKCQ CPOX LAMC2 TIRAP MADCAM1 CTNND1 F5 LYZ TMEM186 TPM2 MEI1 PPP1R11 OLFML3 MIRLET7A2 ELAVL1 GCNT3 FBXL21P SLC35F2 BNIP3L ATP10D LCP1 IGBP1 GALNT8 DCAKD PITRM1 GUK1 STXBP1 ADM LSM2 GHRHR HINFP CD22 CD47 LAP3 IMPDH1 ACP6 PRKACA PNPLA7 ASXL1 IDH1 XRCC5 RAB1A POU5F1 INHA SNX12 KMO NUDT4 USO1 RAB9A CLDN1 FASTK FBXO38 NFKB1 ICOS UBALD2 GSTA5 RF00017-4973 ABCC1 SPHKAP IFNA6 MIR27B PCBP2 MUC13 NUP205 GDF1 SYCE1L ACTA1 MIR422A TDRD7 WRAP53 SNX15 WIPF1 TIMM17B IGF2R AGXT MMADHC RNF181 ATP8A2 AFTPH FGF21 COLGALT1 SBF2-AS1 FCER1G PICART1 DDX39B APEX2 TXNL4B TGFB1I1 UBE3A CLTRN MT1A SNRNP200 TOP2B SHOX2 RNF114 ITGA7 RSPO3 KLF13 TSPAN4 ZFAND2A DNAL1 NLE1 FAM117B AP5S1 PFKFB4 CEACAM1 MED19 SREBF1 ENSG00000237669 PAGR1 IL21 LTK DKKL1 EPAS1 RAB18 UBTF POLD3 TMPRSS12 TPH2 PHB CYP8B1 APOBEC3A_B AKR1A1 TMEM184C HSPB7 ZNF350-AS1 POU2F1 ZC3H15 ELK3 FAM111B LINC01135 SEC61B ENGASE RRP15 PRAL KRT84 RSL1D1 P2RX7 PSME2 RBM25 DHX58 ARCN1 POLR2E IFITM1 PSCA TNDM INO80 CLEC10A GAS6 AMOT TSHR SUN5 GSTM2 ADAM11 NRK NAT1 HGFAC TRAFD1 MTCH2 BACH2 LINC01591 TFF1 BANCR PPARGC1B EPHB2 ACTG1 CENPT CTRB1 CCDC78 MAP1LC3BP1 HAVCR2 MT-TV PLAUR NME1 BHLHE41 MESP2 LACTB SYT16 LPCAT1 ANKS3 DEPP1 FCRL1 SRRM2 PCSK7 HNRNPA1 PBX2 SIK1 BCAS3 HGS POLA2 NOL10 H4C1 ERCC6L2 IFI27L2 SLC16A1 TCOF1 EVL NUDT21 LNX1 GUSBP4 IFNA21 CFD MYBPC2 PYCARD MIR29A ENPP3 ZNF235 ACSL4 ERC1 TRMT61A NHLRC1 KRT18P39 SCO1 SOX2 HTRA4 COX10-AS1 KAT2B COMT KCP MIR26A1 SPHK2 LOC106627981 THNSL2 GPR108 C1QTNF1 TRADD EFCAB11 C1orf43 AS3MT SCARF1 AGO3 LETMD1 TMEM54 KRT75 STT3B B3GNT2 MAGEB2 DUSP13 EYA2 CD1C FANCG DCAF17 S1PR3 ENSG00000261220 ZNF224 CKAP4 AVP EFS PITPNM1 ZNF652 UCK2 MOB3B RNASEH1 WLS OAT DRD1 IQGAP2 RBP5 ANKS1A CLPB SLC30A9 E2F8 SPINK1 YIF1B DIO3 AAVS1 HPN NBN FAN1 SLC17A1 TAF5L RGMB RAC1 MOB2 NFE2 TOMM20 RNVU1-23 AGFG1 APOBEC4 COL22A1 ATP1A3 PNKP POLR1H KRT82 MATR3 PROZ BTLA SEC23B AASDH DCK TIA1 SLC6A4 TOPBP1 MID1IP1 HOMER3 KLF11 SMDT1 KLHDC4 PHLDA3 GABARAP NUP214 TUG1 DPYSL2 GOLM1 AGR3 SOX13 SDCCAG8 KEL IFT46 EXOC5 FPR3 GK AFAP1-AS1 SLCO1B3 DNAJB1 ENSG00000280047 CYLC2 GIMAP8 GPR101 NRP1 NR2F1 PLEK ACAD8 NLRP3 UBE2E3 FBP2 LINC02874 GNAI2 SLC39A7 CAMK1 PRDX6 KIR2DS3 ILF2 NSD2 AKAP7 MIR135A2 NOM1 PSMA3 FARSB ASCC3 CLDN4 DEFB1 H4C8 SLC22A18 CGAS DNAJC22 GRK6 ARHGAP30 RMDN2 EPHA2 MIR18A PAIP2 ZNF879 PROK2 SNRNP70 MTHFR RPGRIP1 ACAT1 AARS1 CSGALNACT2 DACH1 PLK3 SEPTIN1 PHKA2 MIR561 CARD11 RSL24D1 UBE2F TMEM45A MPP7 MRPS34 FBN2 LAIR2 ST3GAL1 PHF3 HLA-DQB1-AS1 DVL2 LCT MTRNR2L1 MIR346 DICER1 KLF16 SSBP4 HIF3A CAPZA2 MIR320D1 AQP10 IFNA2 PLA2G2A FOLH1 MAPKAP1 SULT1A4 SRPRA TRPV6 PIAS3 IRX1P1 LMLN RAB37 FRRS1L YWHAE NEUROG3 MRGBP BDNF LMBRD1 LDLRAD4 LOC102724971 NDUFS8 TEFM TFCP2L1 LOC111674463 EIF2B3 RPA2 RBM4 CCDC26 CSF1 TNFSF10 SMARCB1 DTX3L PLA2G4E ULK1 TAS1R3 MRPS11 SLC2A3 WDR43 MYO5B SNHG18 GNG5 ACOX1 MTHFSD MALRD1 KIF5B UHRF2 CPLX1 NUP153 ANKAR EFHD1 NUDT7 ZNF665 TLR6 GPI ANGPTL7 AGPAT3 ADORA2A CACNG7 KCNH2 CAMK2D CCHCR1 CELF6 RPS27A GCM2 FAM135B IGFALS E2F1 NR0B1 PLCB3 NPAT COIL RASGRP2 SWT1 APOB PIGR CDC25C RAB11FIP2 RCOR3 TSC2 NRP2 CPSF7 FUT6 CDH2 PTPRB ACP2 CECR7 GTF3A MRPL54 LAG3 ARID5B FLNC MARCHF6 KRAS SPTAN1 CDX2 CAVIN1 ACTR1A C4A PPARG ENSG00000267769 DSG2 BBS10 SIGLEC14 IRF6 HSPA4 DHX40 PRRT4 IFT80 LSM10 DLL1 LIF-AS1 IL31RA MYBL2 PPIG RPSA MAPT NAGA HLA-DPB2 C8orf37 PLXNC1 STBD1 MT-CYB CENPO MTTP CXCL8 MGAT5B KCTD5 RLN1 IL36B DYM TOM1L2 METTL15 MAGEA9 TMEM165 HSD3BP4 RPS8 FOXJ2 MSX2 IL17RE KIAA1549L PBRM1 CPB2 MT-TI ZCCHC24 RNF20 FGF19 CTF1 PIM1 EGFL7 USP9X IFIT2 S100G NR3C1 FCN2 NEK7 CORO1B PARP11 MIR103A2 F11 MAP2K6 ATP2A1 AGO4 MT-ATP8 SERINC2 ZC3HAV1 YY1 PNLIP AASDHPPT CKS1B MCM3 RTL5 SLC19A3 IARS1 BNIP3 STEAP4 HTR3B FES YJU2 CCDC120 NPTX2 CBLB RORB MIIP CETN1 CCN2 AP2M1 STAC2 SNAPC4 FAF1 CDK6 NPRL3 P2RY1 TEAD2 CSK VAPB RAB27B IGHM CTSS LILRA2 IFT22 MYOF NEMP2 LCK MIR19B2 QPCTL PRPF6 SOX12 FURIN DEFB103A UQCRFS1 RHNO1 RAMP1 ADH7 KIR3DX1 GAS2L3 CLDN23 APOA5 GGTLC3 LOC105372508 MYO1H EDN3 BLM NALCN SF3A2 FN3KRP LINC00974 UNC13D SMAD7 RHBDD2 PPP1R3B SDC4 HLA-DQB3 FHL2 KRT87P CDC42BPG PSMB2 CADPS IQCH-AS1 FGFR2 ASPA CLDND1 CERS1 MAGIX XRCC3 ITPKA BTBD2 GH2 LMO2 CSF3 PTK7 PPP2CB DDX60 DOLPP1 AP1M1 TNNI2 CD7 RPL22 CYC1 FER1L4 PRB2 CHUK CHAT TTBK1 PDPK1 DENND4B KYNU CPT1B MS4A5 AK9 PEX7 IL22 RPS26 HOXC5 ZNF234 STX11 MIR506 THOC1 CYP7B1 TBXAS1 KXD1 INTS10 ALDH1B1 LOC110599569 USP25 ACOD1 MT-ND4 NICN1 PCYOX1 HLA-K FBXO17 piR-50437-012 CYTH2 SLPI SPEN SULT2A1 PRPS1 GNA15 MT-CO3 STING1 GAMT ZNF217 CCR5AS MIDN MIR939 MIR218-2 NOX4 MCHR1 MPHOSPH8 CYP2E1 GALM ACO1 OPRL1 CTH ATF5 TULP4 PAPPA2 SLC4A2 TP53COR1 STK33 ATP5F1A NR6A1 PKNOX1 PFKFB3 SFRP4 OSTM1 CLCN7 AGTR1 SELENOM ABCB5 MAP3K10 RBMY1J JPH4 DDA1 USP2 MAN2A1 CLCA2 MYCLP1 STRAP RND3 COQ3 RRBP1 IL1RAP MAT2B MTMR10 CSNK1D CCL4L1 PRKAG3 TJP1 KIR3DL1 ZNF599 PTPRT RALGDS PER2 PRMT6 ARFGAP2 ZKSCAN4 CCKBR TAF4 NDUFB6 ADRB2 ENSG00000265778 PRSS3 CD3D CSMD1 TBP RETNLB CTSD SEMA3B JCAD FKBP1B MIR190B IL3 GLI3 VAV2 TWNK TP53BP1 ERCC3 PTCH1 TADA2A AMACR TFRC RHCG AK2 VPS13A LHPP OSBPL6 BCOR AVPR2 MIR197 PFDN5 KCNK12 GRB14 RP2 RXFP1 SERPINF2 PICK1 SEMA4B CSNK1A1 FANCF LONP2 HSALNG0052590 CCNC SLC10A2 ZBP1 CHKA CCDC151 MAP3K1 PBK CPPED1 ALDOA EXOSC5 AZU1 GAB2 DIS3 THAP3 VPS13D IQCB1 SKIV2L KMT5C ATF4 CLEC6A IL12A RAPGEF4 FUZ BMPER HEG1 SLC35G5 MIR125A PSMC1 SLC7A8 RFXANK SPATA7 ADA MAZ PIN4 PDCD10 ACADS GRAMD1A ANXA5 EPHX2 ELOVL4 MIR661 CCL24 BATF CCDC8 MIR744 H4C11 RPL17P50 VIPR2 ADPRS TEF FOXK1 LIMK2 MRPL44 ETF1 HHAT MIR185 UBE4A MX2 HSP90AA1 SHF NDUFC2 TSBP1 DUOX1 LOC109117330 ACSM5 ADGRG6 GPR31 TAMALIN PARP1 SIAH1 NARS1 PLIN1 SH2D4A LHX1 KRT2 MIR3936HG RPS15 GOLGA7 CPA6 MAGEC2 GCNA JAG2 BLOC1S1 CD34 STX3 SLCO4A1 MCTS1 NXPH4 SLC37A4 TGM1 SLC25A25 PKD3 WDR81 PHACTR4 ARPC2 ACYP1 P2RY13 ENG PRKCSH C9orf64 HLA-F RXRG PGP SULT1E1 RGPD4 CGB7 BRD9 HSALNG0044854 GLUD1 KIAA2012 OGT RNF207 PLA2G3 ST3GAL6-AS1 PHIP RPIA MYLIP AARS2 KCNT2 LOC110599567 SLC11A2 NBEAL2 SCAPER ENSG00000227958 NONHSAG031148.2 TYROBP MEN1 NIP7 RARA BDH1 STN1 CARM1 NPHP4 TAF6 SFRP5 IKZF2 MYD88 HOXA11-AS PML TAF1A EBAG9 UROD DAB2 TUBB2A RPL36 ASPM LRP6 RBCK1 SERPINH1 AFF2 TLE1 RACK1 CD244 lnc-HLA-DQB1-2 MIR30B MGLL IL2RA CD24 STXBP5L FAP GPR37 SCARA5 PRG4 CLCA4 LSM6 SMURF1 MLLT1 TPM4 COPS5 TNFSF4 ACADSB BCL2L11 SLC28A2 INHBA CEP78 CDCA3 UGDH PLCB1 LOXL2 ENSG00000237838 SERPINA7 DENR CHRNA1 RPL21 RARRES2 TEX44 JKAMP XPNPEP1 ADPRH H4C12 MIR1908 PIWIL4 RNF26 ADAMTS1 RAP1B ZNF571 ENSG00000267138 PRKG1 P2RY6 RASGRP1 TRIM21 CADM3 CFI NLRC5 KIR2DL3 C19orf71 TRIM7 CYP20A1 FABP1 CDH16 SORBS1 PEA15 NDUFAF2 FGF7 ENSG00000268655 PCSK4 GUCY1A2 IL37 NPC1L1 TAT PACSIN1 UMODL1 SNHG5 FNBP4 CYGB C12orf43 SNAP23 CBL CLECL1 GP9 LOC111216272 CLCN5 FBXW5 SGCA GALK2 FLT3 MTURN EVA1B SERPINB4 CEP97 UBA6 CCEPR UCP1 FGF9 FOXA1 FADD VNN1 SRPK2 ABI1 CACNA1A ALDH3A1 SLC1A4 MIR196A1 HINT2 SPG21 CYS1 GRIN1 OR1A1 CALU PXDNL CDB2 CLEC2L TNFSF13B CMPK1 IGES UPK3A FIP1L1 SSH1 ENSA RHOB GNAQ CKAP2 MMAA CCNG1 MTMR7 AMH HADH PPP2R1A CCKAR HNRNPM EPG5 TET2 COL2A1 IFI30 AUNIP GLUL HK2 ELOVL6 MDK EPHX1 RASSF2 DLX3 AIMP1 PRTN3 SLC27A4 AVPR1A GJB2 ERO1A PRKD1 SOX8 MICALCL ATXN8OS ICAM1 SIX4 ZNF674 BCL2L1 MIRLET7I CD19 RAPGEF3 LGALS9 SCARB2 MIR136 MAPK3 USP34 SLC16A3 STK4 APLP2 lnc-TBC1D2B-4 ADK HDAC2 THBS2 CA2 TIMMDC1 RLN3 TGOLN2 CTBS ETS1 EDC4 MIR34C MPZL3 GATB MIR181A1 KCNK15 HSP90B1 SEPTIN12 CIRBP-AS1 GSTA3 HOXB6 TNIP2 BCAS1 GPR146 SORD NOL6 STEAP2 EIF3C ZP4 TCIRG1 TRIM39 TMEM231 TRRAP RYR2 TRAF1 GRN HSH2D SCAMP1 TRIM32 PTS PRKACG ADCY6 SIRT1-AS DDC CYP51A1 NPR2 ANPEP PROM1 SIGLEC10 MIR10B TRIP4 ZFYVE26 CFAP410 GDF5 CSH2 TBX20 ALPI DST GLRX5 RAB5C FLAD1 MSH2 PIP4K2C NPPC ZEB2 CYLD LOC111832670 WTAP C4orf54 NECAB3 PET100 MTMR2 TSC22D4 CHRM2 MIR1-1 CCNH NR1H4 RRM1 GABRA4 C1orf35 SSTR1 RAB42P1 CTDSPL APOBEC3C SALL4 PDE11A SPPL2C ZCCHC8 RAD17 LILRB5 CD5 ABCA4 OPRM1 ACER2 CLYBL CYP26B1 CMBL LECT2 SRSF2 PCCA AQP5 GNB5 CCL21 H4C14 PRKAR1B FMO2 IL16 MSTN VCL DIPK1A TCF3 ZSWIM7 SERPINE1 HFE RAVER2 SMC5 SLC13A5 SMN1 ENSG00000270679 BACH1 NRDE2 PPP2R2D DTL PORCN CENPV LPAR6 FAM124B RSAD2 CTBP2 CDH23 INTS4 TTN PLLP SLC26A5 SRGAP3 NAF1 PDK1 NBR2 LMTK3 MIR194-2 LINC02055 GACAT2 MEG3 RAD51 RPL13A ALPG DYRK1A HTRA2 SARDH ANGPTL2 ERAS HBS1L CPA5 TMEM30A CRK NCAPH2 RNASET2 NEDD9 HSPA1B PSMD13 MYRIP CFAP52 LY6E ATP12A AUP1 PIP GSTO1 HNRNPA3 ENPP1 SCNN1A PLPP5 LSM1 IDUA P2RX4 CCND2 RPS3AP42 CUL4A PDZK1 CFB UBIAD1 HEMGN CDC42BPB CXCL6 SLC34A2 MIR411 TARP ACTA2-AS1 CAPN10 SLC6A9 NDUFS5 CCR5 VN2R20P PDLIM3 ZNHIT3 SNRPD3 SCN8A UNC93B1 KCTD16 SPCS2 CSF2RA PAX3 MIR425 H4C3 HSD17B8 PRKAB1 KIF20A YAP1 YBX3 ITPKC SIK3 RAD21 L3MBTL3 RPUSD4 SNRPB MIF MT-TT IRF8 MICALL2 ENSG00000256029 ACD BCL3 ITGB3 TCEA3 SPATA13 CEP152 CFHR3 piR-41245-246 SEMA5A lnc-HLA-DRA-3 ZNF222 TTTY5 INHBC GNG13 F12 MIR432 DEFB123 NAA25 SUPT6H GSK3A GIPC1 ELL2 CEMIP2 GPR89A TMEM199 FZD1 AGAP1 TRPC4AP FREM1 SAA2 IFN1@ EIF4G2 ZNF385A IGFBP4 MIR95 CD2-LCR HNRNPH2 NDUFA10 CLIC2 CCDC92 KCNK5 CD69 LTA TTPA IFNB1 CLNS1A FUNDC2 PEX5L GPA33 MIR499A UBR7 ING1 BCAT1 SLC2A9 ARL17A KLK11 DNER MED22 ETV6 TMSB4X CEBPB FMNL2 MIR873 HMGA1 CCL3 ALKBH3 TM6SF2 RANBP2 LIG3 IRAK4 ECI1 KDM3A MLPH RETSAT NRG1 CAST TTK TSBP1-AS1 TGFBI DDX41 PGM3 PSMB10 SLC4A11 UBE2D2 GABARAPL2 MARVELD2 TAOK1 CISH ZNF683 SPINT2 GIT2 CLDN18 NCAPG RASSF6 HSPG2 ABHD6 TIFA CD274 ENSG00000272953 FDPS DUSP9 SLC52A2 TM4SF5 NT5C1A SUCLA2 NANS ATP10B SCNM1 FUNDC1 NPAS3 PRKCA GGA2 TRIB2 CCDC115 TTC1 CTC1 ATP8B1 CLN3 STX4 TUBB1 GLUD1P5 SCN5A RUBCN GRIA2 VHL NUSAP1 FGF23 PCNT BCKDHB IFNA8 FLRT1 SNX17 HDAC6 MIR375 SPART FTX HBG1 OTOP1 CFAP45 SPOCD1 GHRH SMCR8 H2BC17 IL11RA GDF3 RPS6KB1 DNAJC19 ATP5MD TOP1 MIR320C1 HBA1 SHISA5 RNF169 ADIPOQ MPST DPM2 PLPBP SLC18A1 CD44 ABCA3 RPS17 KRTCAP2 FARP1 TUBD1 GNL3 BTG2 ITGA5 ATF6B LOC107882133 MEFV NDUFS6 ECHS1 NMBR GLT1D1 ABCB7 VPS4A CYP11A1 PFKP ABCC6 STOX1 NCF4 SLC25A3 ZNF646 MTHFD2L TSPY1 RAB1B IFT122 LDB3 HOMEZ NTRK1 MSL1 UBAC1 NAP1L2 TOP1MT CNGB1 NTSR1 GPSM2 ENSG00000267030 NOS2 PRKCG CHORDC1 SLC2A5 TRIB3 C2orf49 DDX5 RGN COL4A5 CCNB1 HROB CCDC28B KCNK3 FGR MSRB3 COX15 KCNK6 HLA-DRB4 GDA XK AQP9 SLC15A2 HEPH THRAP3 MET ING3 RNF185 TNS3 KNDC1 C4orf3 SRPK1 ENSG00000126500 MED13L DUS2 LY6K NFIA PTCD3 TEAD1 SNX33 RPL7A LEP IRAIN UBA1 GLRA4 MAPKAPK2 ATXN10 ECH1 NMNAT1 CCL22 PTPN22 CYP2F1 ITGA3 UNC45B CYP2D6 LPAR4 RUNX3 LUZP1 PRKCB C5AR1 SIGLEC8 ATP5MG A2M CDK20 LMNB2 ROCK2 SNX16 ANXA3 VKORC1 PPP1R15B ELANE UGT2B7 FOXK2 EED ATL2 SLC8A1 PDCD5 DGUOK HES1 CLDN16 FGGY LINC01138 CLC ISL1 GAGE1 C1QTNF3 VGLL3 GRK1 MANEA RIEG2 CD1A SUPT4H1 SRRM4 TRAF5 GARS1 RAPGEF6 lnc-GRM6-4 ANO5 NEIL1 EGOT LINC00261 TBXT STARD5 MFF STAU2 EXOC3 RAD23B PGAP4 NCOR2 TNFRSF9 KLK8 CCNE1 PKDREJ SSU72 GPR78 GSTA2 SMUG1 UFM1 CORO2A USP14 APOE NR2E3 ST8SIA4 GMPR ING5 ORM1 HIKESHI HSPD1 F2RL1 LOC106014249 MIR128-2 ADH5 MIR1225 SHBG CDIPT MIR200C TUBB TOR3A PREP ENSG00000232043 ENTPD8 RFX3 MYH6 BCHE TONSL BCL2 ARL13B piR-57133-370 PLCL2 SNX19 ZNF284 NAALADL1 DUSP5 FCN1 NRIR TNXB C1QL1 A4GALT ATP11C BIRC7 TOMM34 XRN2 CD6 COG1 CRHBP THRB MYBPC1 RNF39 PSMD11 ALAD AQP2 SLC9A9 CFAP221 GSTM4 CDC42BPA F7 CNOT1 PTENP1 CEP120 B3GALT5 LUC7L2 SPTBN1 ENSG00000247853 IRS1 LSM4 CDC42EP3 SAMM50 SRP72 MFN2 TSPAN7 LOC111365141 MTX1 NUCB1 ATR ARL16 TACR1 GBGT1 CDK17 SEPTIN4 RASIP1 DPYD MAPK10 ECRG4 NCAM1 PRDX4 AGT KRT71 GLIS2 RPS3AP34 ATF7 PILRA ABCF2 TARS1 FGF1 IL6R NFIL3 COX10 DNAJA3 GCA ECEL1 GLG1 CLDN6 CIITA EPHA4 FANCC MUTYH ZNF227 MRPL15 NQO2 KCNQ5 NEU1 SELENOP ZBTB20 HP1BP3 MED14 H2BC11 ENHO EPGN SPRY2 TSN LZTFL1 XPC GMFB CCR3 HBG2 PDIA3P1 ALDH1L2 NOP58 CEP85 PCSK6 STAT5A PTPN2 SF3A3 HCFC1 NFYA SLC3A1 NRG4 PIGF AHNAK ACTR5 KIF14 TRMT6 DUSP18 IL17C AHR MIR4638 PTPRH ATP6V1C1 ATP10A PLPP7 SORBS2 NFE2L2 IL23R SPINK5 NRF1 CYP4A22 H3C1 OPA3 IFI27 TAGAP MT1G IDS MILR1 PARG MICALL1 SFRP2 TRB JPX CEP131 TBX21 DUS4L KCNJ6 TNFAIP6 ZMIZ1-AS1 RTEL1 CCT4 PCAT1 CASP5 CHMP6 MIR199A2 BRD4 MIR379 CYP19A1 NDUFA13 CD151 TOMM20L ARFRP1 TMEM238 HRH2 MTMR6 UBTD1 SOX9 CRABP1 TNRC18 SLCO1B3-SLCO1B7 FAU ENSG00000218027 DTNB GPR158 RPL22P3 CARD9 SLC4A5 ZNF137P FBXO46 SNORD35B SVIL OAS1 PHB2 WARS1 ADCY3 NDRG2 CELF5 GRIP1 MIR29B2 IGSF9 TCF21 ENSG00000285404 AMELX SLA JPH2 MYCBPAP MIRLET7F2 SLC51B TRAM2 LXN TLK1 UPK3B UNC5B-AS1 KLHL21 ZNF384 RER1 ELAVL2 KLF2 PMVK RPN1 FAM160B1 SIX2 EIF3D HPS1 RNF7 TFE3 KCTD13 MIR571 DLGAP1 FBF1 IRF7 SET NAB2 MIR519D FAM120A ASCL2 SHH SH2B1 THPO TYRP1 LAMTOR3 EIF2S1 TNFRSF17 TAF9 LOC111674464 TERF2 TNFRSF1A ACADVL CD8A GTF2H5 ISCU MIR17HG TNNT2 PHGDH XPO4 FAM131C XIAP CA9 HCAR2 GPR62 GPS1 MIR491 RPS29 GPR174 ATP6V0A2 KIAA0319L SLC29A3 LGALS4 SDHD OSM SUMF1 USH2A NF1 MIRLET7E APOBEC3A OLR1 TRIM63 LYPD3 BCL7A AGER MAP4K3 TCF19 CBS ATP5PO KIAA0753 MICA FIS1 ELF4 CLIP2 IGLL5 KCNV2 MUCL3 CLPS LINC01194 KDELR1 NT5E NCSTN CD83 GALNT9 IL18 NPY GCGR BEGAIN FOXRED1 SLC16A2 SC5D IGFBP2 NOTCH2 SIGLEC1 CD93 SULF2 CEP164 ADD1 TOR2A ZNF136 KDM6B LOC111242783 TUSC7 ANP32A MGP SOST TRPC1 ACLY USP43 CYP4F12 FKBP5 CHCHD5 SMS MAPK7 VIRMA CCDC85B WIF1 GCH1 FLI1 DGKQ GPRIN2 CPA4 PERCC1 MELK IL15RA CUL3 HMBOX1 PODXL MUC20 GPX2 ITK CLIC5 KMT2B DNAJC4 NR1D2 FBXL5 DEFA5 MEF2B UQCRQ ITGB2 PTPN4 STX5 ENSG00000278769 PHPT1 CD72 KAT5 MIR361 VEGFA XRCC1 PARVB LAMA1 MAS1L ELL CDH11 PGBD3 NDC80 GIMAP6 AREG NRSN1 LIPT1 ENSG00000212273 SENP3 GRIN2A CAVIN2 MAN2C1 RNVU1-32 COG8 SGO1 SNHG32 MYC NRXN1 SLC37A1 SUPT16H MUS81 PHYH ULBP1 OIP5 IL10RB POU3F2 RPLP2 FGF22 SPNS1 IL1RAPL1 E2F7 PCSK9 VCP NKX2-1 BGN LAMA3 APOBEC3F COCH LZTS3 SRSF3 SLC11A1 ICAM2 MIR21 SH3GL1 FAS-AS1 GSK3B RALB PDXP KIR2DL1 TNFAIP3 NDUFV2 BLK PANK3 GBP4 WDR82 APOM SPATA16 USP51 TESK1 MMP1 CEP104 SPINK13 NCOA6 HLA-DQA1 GCLC SEC61A1 UPF1 CTDP1 ZMYND10 lnc-DDRGK1-1 MIR518D IL18RAP KRT86 SLIT1 CDC40 SLAMF6 CRNDE MIR326 PRRX2 MKKS TRIM47 CFAP74 ACOX3 CD109 ADCY1 RHBG SMURF2 TP53I3 SRL ADAD1 EBP SLC22A3 TOR1A EFR3B P2RY4 GPBP1 TRPV1 RNU6-1019P ADAMTS12 PES1 SERPINA2 UCP2 MIR28 FOXG1 MAGEA10 OR2AG1 WFS1 PROC PABPN1 SLCO1C1 SLC22A5 DEPDC1 KIF23 KIR2DS5 QSOX2 PPARD KDM4A URB2 YBX1 STK24 SPEG H2BC4 SCTR AAAS DACT2 ABCD3 FIG4 IGHMBP2 SPRY4-IT1 FAM3C BHLHA9 EIF2AK2 DBH-AS1 AP1S3 CCL11 PAM IDO1 MRM2 HOXD8 CITED2 SLC39A4 GPIHBP1 MIR195 GRAMD1B FOXP2 POLR3A MIR501 ENSG00000267114 LALBA H2AZ1 ADGRE5 KMT2D BPIFB1 MMP3 CRHR1 WDR70 MIR590 CCL18 RINT1 F13A1 SLC10A1 BRCA2 ACADM FMR1 DUSP3 CHMP2A KDM7A CXCR1 BBS9 UNC119B C9orf72 SLC12A3 ZDHHC7 ZPR1 APELA SDSL ERCC4 MMP8 HACL1 PLA2G12B MIR34A ACY1 PPP5C MIR222 RNASEH2C ELFN2 IRAK2 SCAMP4 LOC110673974 MYO9A CCDC3 ADCYAP1 SP110 MIER2 SH3BP2 PNMA2 AGRN CEP290 TFB1M PRPH2 GOLGB1 MSRB1P1 DXO NFKBIL1 TLR10 SLC30A8 RPS10 HIC1 TLE3 IAPP PSMB7 GPC3-AS1 SHQ1 MOV10L1 COMETT ADHFE1 OCLN NAGLU NELFA FAM117A SPRY1 MYSM1 CDON IGKC MMP14 SERPINI1 THSD7A OVCH1-AS1 CXCL2 LHX4 DHRS12 IZUMO1 HSP90AA2P LOC111255645 SLC10A6 APPL2 TNPO1 CARD10 EEF1D SOD1 PAPOLG CHML PACS1 BCR SIRT5 CAPN2 TIAM2 FZD5 NR4A1 CELA3B EGFR-AS1 RPL15 TRAPPC11 APTR PRC1 LINC00589 ABCB9 HOXC4 MIR675 STAB1 CEACAM5 MELTF UGT1A7 LRRTM2 FUT3 GIMAP4 DBI PIF1 LPIN2 KIR2DP1 UFD1 DIABLO TRHR RPS6KA4 ARMC9 RNF152 SLITRK3 TGFBRAP1 FAHD2A BBS4 PFDN1 CCL3L1 HOXC10 CLPTM1L AKR1D1 ATP6V0C PRPF8 PSMD6 IPCEF1 RTRAF NOX3 MIR224 C2orf72 D2HGDH RPL18A HM13 TCERG1 KRT16 UBE2O UBL5 APOLD1 piR-61240-289 ARHGAP29 TNFSF8 PROKR2 CDR1 PDE5A FBLN2 HBE1 TMEM167A FOXI1 CPEB4 APOOP1 RAB4A BCKDHA MYOC CKMT1A RPL3 THBS1 APOO FARSA PPP1R3E CMAS EIF4ENIF1 HEATR9 SLC30A1 TNFRSF21 TMPRSS7 PLAC1 KLHL18 LOC110366354 TBX3 CRIP2 TAZ DAXX DIO2 GPER1 MYDGF UNC13A SCIMP TAF11 TARBP2 CABIN1 TRIOBP PPIAL4F APLN UVSSA NCOA1 LINC00598 CDK7 IL25 SNCG AMDHD1 CHEK2 DRD4 GCN1 GDF11 CD247 MCM2 NEURL1 CDAN1 TGS1 MIR155 LOC110740340 MCM4 PKHD1L1 PDZD2 TOMM70 MCC IFT88 RPL9 RAB32 P2RX2 ENSG00000267255 MIR101-1 ID2 ARHGAP5 ZNF688 CETP APOC2 PTPN6 BAHD1 DNAH10 GRIK3 METAP2 PAN3 UBE2V1P1 PDHX MTA1 GNG11 CLCN3 NABP1 CDK2 GPAM LINC01271 SLAMF7 CDC37 TENT2 INTS2 GRK4 PPP2CA POLR2A SEC14L2 MIR219A1 MC3R PHEX MSMO1 CCZ1 MECOM POLH CAMTA2 DDX17 PLEKHA2 SCN7A GAS1 ESRP1 DGKA PEG3 SGSH SLC35G2 CXCL10 GALT MCF2 STK32B TYR ATP6AP2 TACR3 SLC35C1 KIF1A RSPRY1 VCAN HERC5 SYDE1 UBE2A MED12L DDHD1 SHPK MAFG LINC-ROR ALKBH5 MIR140 JUP OSBP2 CBX3 CARNMT1 MIR138-1 KBTBD2 KIF3A DCXR CDC23 PBXIP1 EHD1 CYBC1 MARCKSL1 RAB11FIP5 CDKN1A NCK1 KCTD8 SCAF1 ZNF550 DRAIC LSR L13304-025 CUL7 LIPC DIO1 GSTZ1 OMP CTRL PAK4 RNF10 FAT1 PATJ ISLR2 MAGOH CENPB NREP GATA2 CELSR2 SNHG12 PTPMT1 GPC3 GRP SV2A MAGEA12 TAF1C GSG1 PDGFRA CACNG1 CIAPIN1 CSTB PTPRJ PLEKHJ1 HILPDA SNHG6 RPP21 UCN3 FPR2 TTC14 MLKL CCNF SLC22A12 NMT1 AKAP8 SOCS5 MVB12A HADHA MRPL58 PGM5 COL26A1 TFEB RND2 HDAC10 ATG12 BMI1 MASP1 SLURP1 MBNP SWI5 BOLA3 FOSB ASB5 KIR3DL3 SEMA3F SLC27A2 TMEM135 ARHGAP35 G6PC KRT33A PREX1 CPE GNB1 CXCR6 TRIM46 HLA-DOB HPSE UBE2V1 DMWD POLD1 VIPAS39 LCAT AIG1 ST6GAL1 POMC ZNF404 SKIL RPL21P41 HMGCS1 RAB10 CDIN1 ALAS2 WRNIP1 EGID-106632268 ABCC13 NFKBIA CNOT3 GAK PSMA6 CRTC1 GRAMD2B CPT1A ALMS1 EIF3I DCN SUCLG2 MIR24-2 FOXF2 MLH1 S100A12 LINC00554 ACTN1 AMPD2 IFNAR2 MRPL36 CYB5A NRBF2 MKS1 FBXO6 CX3CR1 PDE1B KCTD3 ITGAE PCNX3 HTR2A-AS1 PLP1 KISS1 MICA-AS1 IFRD2 PTCHD1 CRLF2 NARS2 DOCK7 SERPINB3 TMLHE LIN28A MARCHF1 lnc-C20orf194-1 ARHGEF19 TSFM ABCC8 BPI MAP1S TTC4 BNC2 NDUFS3 MAGED1 MIR718 MIR657 STMN2 MTCO3P1 MT2A KLHDC9 HSD17B14 ALG1 ZNF674-AS1 ENSG00000268670 HEXA HNRNPU USP39 FLT4 MAP2K3 SCAP MIR483 ADAMTSL1 RFC5 BRAF BABAM2 MTFR1L MIR20B PAX5 GJB4 PEBP1 AAK1 FBXO2 COL1A2 RMND1 LOC105374882 GMEB1 AKT3 AANAT CRB1 UBR4 LTBP3 CTTN PROCR PASK ZNF697 USP8 BAIAP2 SI GULP1 KCNE4 KCNS3 CELA2A BAAT TMEM37 MBD1 ITGAL GKN2 ARFGAP1 CH25H CYP4B1 H1-4 LYPLAL1 ALPL TRIM62 BTAF1 UQCRC2 TTC26 ACKR3 MAEA HYAL1 H1-2 NCR3LG1 PSD3 ISCA2 AGR2 PNP HDLBP CHRNA7 PIGM GNB3 ACY3 HMGB1 ACTR2 FAM174B EIF2D SOAT2 CDIP1 DARS2 CDC25A CEP295 BAIAP2L1 ESM1 B3GNT5 CASS4 PTGER3 RFLNA ARHGEF10L ATOX1 USP30 KANK1 BCL2L12 ZNF225 DNASE1 STT3A PTN MIR193B RAE1 GNG8 CCNA1 RYK IL26 RPL10A BID IRS4 MACF1 CXCL14 TGFB2 CHRNE HSPB8 NUFIP2 ZDHHC2 ADD3 MBNL2 ADGRB2 MTA2 RAP1A AXIN1 POLR1C TRIO ID3 FCRL6 WEE2 GPR107 MTM1 CAPN3 CSNK1A1L NLRC4 AP1G1 RNF170 FOXQ1 SMARCC1 KBTBD13 GNAI3 KCNE1 NRM POLG2 CD4 TRMT61B ITLN1 RNU6-1 IRF1 PTPRE PTK2B UNC5B SDHB NXNL2 CLRN1 PLAAT4 SOX4 NUDT10 IFI27L1 SH2D1A MYO3B USP20 DERA L3MBTL2 APOB-ICR MCRS1 TPP2 PKD1 JAM3 PHKG2 IL21-AS1 DOLK TUBAL3 ACVRL1 CLN8 ABL2 PTPN12 CRY2 H2BU1 LAMP1 FCGR2B PNPLA4 MAP3K5 RXRA ABITRAM CLDN11 TIMP3 LINC01772 ARPP21 CIB2 MAPKAPK5-AS1 MAML2 MIR98 COG6 NFATC1 CCR9 SP6 RAC2 SCYL1 UAP1L1 YOD1 RALY TBX5 RHD HMOX2 YIPF4 LINC02027 ZNF513 PLCG1 FOLH1B ENSG00000268030 HTR2C TAGLN2 HAGHL MDH1 MIR370 ITGB4 MIR125B2 SYT10 FMO4 SEPTIN6 DRD5 TMEM30B UBE2I DUOXA2 LOC117307477 ANGPTL3 HRH4 LUARIS LOC102724122 MYO6 ATP11A RBM24 MUC16 SLC25A5 MYO1C RBM23 FAS KIFAP3 GLRA2 BTN3A2 NPB ATP5F1D CSTF3 OSMR SOD3 CYP21A2 CYSLTR2 RPL12 C19orf25 WIZ ALDH4A1 NLRP6 RIOX2 CRYAB TRNAU1AP RNF17 NEIL3 COPA SLC26A3 KIF11 SKP2 ZNF175 MIR1-2 SYNC PARVA PTHLH SLC40A1 SNORD118 PKLR MIR27A RNF34 CD36 COA6 FAM83A-AS1 CCT2 RBMY1F PRKG2 ARF4 ANAPC13 LMF1 SLCO3A1 MBTPS1 MIR215 NCF2 SH3GLB1 SLC12A6 MRPL48 MIR320A XRCC6 SUPT5H RANP1 SOS2 H3C14 C1QTNF5 NNAT USP16 MIR1269B ZBTB2 ABO TRAF3 LETM1 ALG14 USP10 NIPSNAP1 F9 CNGB3 LIPE PI4K2A MED8 MIRLET7C KLRA1P STAT4 RNF145 LY86 CLDN2 MRGPRX2 ZMYND8 SNRPA GIHCG GATAD2A PDXK PTGES3 CCM2 TAP1 CYP11B2 ETS2 MIR181D FCRL3 PRDX1 FGB ZNF382 LINC01116 CCAT2 COX17 INTS8 ITGA1 PIWIL1 EPHA5 FAM24B FOLR1 MYOT HAL BCL2L2 OCTN3 EMG1 MERTK BAG1 MYNN ORMDL3 TBXA2R TECR NEAT1 NUPR1 CCL7 ITGA8 DCLRE1B TP73 PRKCD DSC2 NDUFB4 TRIM65 TMPRSS6 ANTXR2 COL4A3 PUS1 MIR92A1 PEX1 BCL9L OLFM1 LOC79999 RBM19 RET TAPBP RUNX1 IL1RL1 ALX3 MID1 TAC4 MAPK9 ZNF366 RIPK2 PPTC7 KIF4B CLDN7 GLP1R CSTF2T TRIM37 LRRC25 GRHL2 IREB2 FAM215A MIR124-2 CNR1 ABCC4 NAA16 LOC108660404 MAGT1 EPPIN MS4A6A FTL PCLO CYP4V2 PIK3C2A IFITM2 ARNTL2 ASPH LEPR EARS2 ERN2 GH-LCR VPS51 PRH2 CDKN2D PITPNA PGLYRP2 KDM1A STUB1 SLC25A22 ILK SLCO1B7 SLC22A8 SLC22A1 MRPS7 RASA4 PITX2 MYBPC3 FABP3 GJA4 TRPV2 SIM1 CDADC1 BLID TFPT TSPYL2 EIF2S3 AP2A1 ENDOV FZD7 CHL1 VANGL2 TDGF1P2 MTRNR2L5 FA2H TIGIT ALG13 TRD GSN EGR2 SMARCA5 MIR363 FRMD6 lnc-NDFIP1-2 CDR3 EFNA1 LOC107963949 SH3GL3 MYCNOS AKR7A2 PRC1-AS1 CERKL VEGFC H4C5 VLDLR LARP1 ITGB6 DBT SLC1A2 INVS MPO ZBTB16 PDGFB MIR32 RFX1 NTF4 VIM REG1B UQCRB SUMO3 LGALS3BP HOXA10 SAMD9L NGB LZTS1 ABCE1 CSNK1G2 SUPT3H ADAMTS4 MIR99B CD27 SLIT3 EGLN1 PEX13 GRIN2B COX20 HSPA6 CACUL1 ATP6AP1 NR1H2 ARHGEF39 PDK2 EGILA DCAF1 SLC38A2 SLC25A29 SPHK1 TRIM26 ZNF578 ORAI1 IL34 RUVBL1 ADAM9 APAF1 NEK1 MYH11 MPP2 TNK2 MATN3 AGK IFNGR2 NDUFA4 ZNRD1ASP ITPR1 TBC1D20 DUSP1 DDAH2 BCAR1 ATXN3 TRIM27 CDC42EP2 P2RY2 H19 SOX17 TGFBR1 RNF187 MRC2 GBP2 CDC42 GRK2 ENSG00000269918 LRPPRC LOC110386947 NDUFAF7 PTER POMGNT1 DUSP5P1 ATP1B2 RBX1 GRHPR ANKRD54 MYO18A TAS2R1 SEMA7A ZBTB7A GARRE1 ATM TIE1 ODF2L L13708-003 ENSG00000269148 CRYBG2 GTF2H4 BLOC1S3 ZCCHC3 TINAG NEUROD1 RNFT2 PARP2 TFF2 NDFIP2 TMEM75 IFNA5 piR-51600 PCGF2 CYP2A13 PLCD4 TNS1 KCNH6 CD9 ALDH7A1 EIF3F VDAC3 CASP7 KCNJ10 USP53 CAMK1G MCRIP1 MIR216B E2F5 ACP7 MIR128-1 WFDC13 ACSL5 SMAD4 SLC35B3 AFM G0S2 RRP1B STAT6 CCDC40 ZNRF2 THOP1 ASB16 SCARB1 CACNA1F ADCY8 ARMC10 PSG1 KRTAP5-AS1 VARS2 PTPA GREM1 NFIC ITPR2 SUN2 IGHV4-38-2 PRKAG2 AGXT2 OR6K3 COX4I2 PLK4 MED16 RAB7B GOSR1 RPL30 IGSF5 PLOD3 ELMOD2 HAVCR1 SHC3 DYNC2H1 SPCS1 SRP19 MMEL1 LRP10 KRI1 PRXL2A MGMT B3GLCT BUB1 PIEZO1 DYNLRB1 AIF1 CSH1 PANTR1 MAP4K5 EXOSC8 LASP1 SLC28A1 VPS35L KRT6A MYO7B PTF1A SEH1L GPHA2 PANDAR SIGMAR1 MCL1 ATAD3A OSGIN2 PRNP PDIK1L SHMT1 POPDC2 CRISP3 ADCY10 TRIP13 RSPH6A FOXA3 SRSF4 TLE5 ASGR1 IL6 IFNA16 RAB17 FBXL2 PTBP1 USP37 BRD1 LPAR2 STATH ZNF335 CPT2 HEATR1 IVD PSME1 SPRED3 ATP5PF EPOR MVP MTX3 TMEM94 PSTPIP2 LYPD1 SF1 PNLIPRP2 ZP3 NUP210 RAB11B UBASH3A YWHAB NNT-AS1 CUL1 DHX9 CNEP1R1 CXCR5 SLC7A2 CLK1 MRPL43 TNFAIP1 HSD3B7 PKN2 VDR LOC110806262 ACE ZNF333 SMIM24 PSMA2 INTS11 KCNQ1-AS1 DUSP11 BCO1 SH3RF2 NEK11 DLL3 CNTN3 CREB3L2 CPA1 RTN4 FASLG PPT2 FOXP4 MIR126 WASHC4 RPL26 GNL3L RPL28P4 NDUFB10 CACNA1D PRKAA2 UHRF1BP1 CD163 CD37 GIMAP2 DPT MIR330 PRKAR2B PRDM8 KRT14 BBS2 ABCG2 PGAM5 FABP5P3 GRB10 SLC8A1-AS1 CLSTN1 TBL1X INSIG2 STXBP2 FUT1 MIR148B DFFA COQ6 LATS2 MIR205 MS4A1 NOCT CA3-AS1 BTN1A1 NT5C3A TMEM65 CBLIF CHP2 WDR46 LSP1 CDK9 CD59 ENSG00000258798 ERP29 TTR DDIT4 MAOB PTGDS BST2 ONECUT1 CYP1A2 STX17 MRPL3 POMP TMPRSS9 BMPR2 ALLC KLF7 EAPP NME2 GCSIR CDO1 IFI6 FRMD8 MGAT2 GPR183 SLC5A6 LEPQTL1 NOP56 MT-TF HIVEP2 MYRF SUMO2 CTSG TXK GRIK1 HACD3 ICAM4 FPGS ANAPC2 ORC2 CERS5 OTX2 SLC5A5 IL12RB2 SLX4 REG4 EIF5 PALB2 SEPSECS RNASE3 PIK3CG ANOS1 BCORL1 CD5L POLR2K NBPF4 C8B PREB SASS6 HNRNPF TUBA8 STIL CYP3A7 FHOD1 HNP1 TFDP1 DHPS RPL5 FFAR4 LINC00383 HCCS DENND1B LHX3 MIR23B MIR200A CD79A S1PR1 CCDC18-AS1 SYT3 IL1R2 TEX35 MIR9-1HG SLAMF9 PIK3CA TNPO3 COLEC10 NBPF10 IRGM ITIH6 HTT HOXC9 DCUN1D1 KDM4C DAB2IP SLC6A5 ERBB3 ZKSCAN8 SDK1 CYP2C18 SDC1 SYK HNRNPDL IFI44L NUP58 RPL3L TDG PMP2 PLAG1 MYCBP2 AGPAT2 SLC12A1 NAT16 ENTPD4 CYP27A1 XPO1 DCTPP1 WDR35 KCNN1 CCDC80 CAVIN4 SNORA66 MIR142 ZNF841 PEX3 AZGP1 CLU RPS12 MED1 CXCL12 ABCB10 IDI1 C4orf17 KCTD6 ULBP2 OPRD1 CCL16 CST6 SACM1L CD63 LGI1 KLHL12 CRYBB1 TOP2A CX3CL1 GYPB NR5A2 FEN1 JSRP1 IGF1R FAR1 SNRPGP15 KIR3DS1 PCBP2-OT1 ODAPH IFNA13 MAGEA6 DEFB126 MIR191 PKD1L1 CAV1 ENSG00000255627 ALDH1A1 H3-2 DGAT2 HPRT1 RNASEL HELLS DNAH9 FANCI TNS4 NAV1 IGHA1 PSMA7 DBF4B GET1 TTF1 TUBG1 PLAGL2 HMGCL MSI2 ABCD1 MRTFA SPARCL1 ACAA1 IL6ST RNA5SP125 PRSS23 FAM172A ANK1 MMUT IL22RA1 PPP4R3A TEC PSMB5 APCS MTRNR2L3 ABCB4 USB1 SFTPB MXD3 SGMS2 CARTPT HNF4A CNTNAP2 TNFRSF10B RABEP1 FRK TBX19 CTDSP1 CDK5R1 CHD4 MIR503 OPN1LW IKBKE HIF1A RELA MIR133B LRWD1 ACAD10 RPS19 C1QB TXNDC9 KHSRP TNFRSF11A FBL MATN1 SOX2-OT CFTR CAMK1D RTP4 MIR96 DYNC1I1 PPP4C SLC47A2 LINC02210 TREH CD48 KMT2A ST14 GDI2 LOC110467516 PKN1 XBP1 FDX2 CEBPA ACSF3 RF00017-5474 ARL3 SURF6 BPHL MTCL1 ATP6V1G2-DDX39B COX5B ERCC6 S100A10 TDGF1 EIF2AK4 RRP12 PTPRO CLEC12A CD3G PPAT SIRT3 CARD8-AS1 NIPA1 C1orf189 ACTB IL7R OTUD7B MSRA DMAP1 IFNA4 TLR4 ANKRD12 BSCL2 NSMCE3 CFHR2 SLC30A5 AZIN2 ACVR2B SUFU LMO3 UBQLN1 NEUROG1 LOC111216291 SOCS2 XKR6 CYP2C8 DOCK5 MIR17 MIR107 DSEL ROM1 UBA5 SFTPA2 CD1E NOX1 OFCC1 LOC117038795 SRFBP1 COPB1 SRSF9 TMOD1 REXO1L1P DHDH RABL3 COQ8B MRPL21 INSR TLR9 GLRB HLA-DMA UBXN6 ITGA11 CRACR2A CTU1 CBY1 PGC FGF3 CLEC16A SLC35A3 IER3IP1 RASAL2 MIR383 SYNJ1 MMD RADX NOG POLR2J2 KLF15 HLA-B ATP5F1B PPP2R2B TCF7L2 CHD5 JAK1 MT-CO2 GPD2 GALNT11 GPX5 FLOT1 MMP25 ULK2 PIGO CHST5 NRCAM CSRP2 LYRM4 HIPK2 GPANK1 ZMIZ1 DAP DDX58 AKT1 ENPP6 GBA3 CDC34 MCM6 ABCA7 EIF4A2 LOC117204001 RPGR RRM2B COL6A3 MIR130B FABP12 USP22 PTCD1 GNPAT LOC110599572 WDR18 CDNF HSD17B12 FAM107A SUDS3 CYP2B6 H1-0 CAMKK2 NES PHETA1 TBC1D2 SLC25A12 APOC4-APOC2 PON2 MUC5AC EIF3K HPX MIR342 MALAT1 PLB1 CLEC4A CT70 PRR12 MLXIPL MAP2K1 GH1 SLFN5 PTP4A1 MARCHF8 CARF UBR2 PPIE FCRLA MAK ZNF613 MIR217 PIGW PGLS OAZ1 WNT4 IL11 YIPF5 ANKRD34A CSN2 TCF7 TDO2 MMRN1 LCN2 AKR1C3 GKAP1 CACNA2D4 GMDS SPARC MIR22HG CBX5 UBE2V2 COL5A1 ASB7 DERL1 FBXL18 SERPINC1 KCTD21 F3 MTCO1P2 GTF3C1 PLEKHM2 NR1I2 EFCAB1 APOA1 SEPHS2 TATDN1 PYGM KPNB1 WNT5B MIR188 PRSS57 FOXO3 KHDRBS1 LARS2 SLC25A38 PPP1R18 DCTN2 FTCD PSEN1 IFT20 CTHRC1 HULC UHRF1 CDKN2A CARMIL2 DLX1 TSHB ADGRV1 RELN RASGRF2 MAMDC2 SSBP1 CASP2 PDIA4 SERBP1 ATHS CSF1R JUN AGMAT NAGK MYL2 STK38 RBP1 QTRT2 HSFX1 TREX1 TNFSF14 PRRT2 CRISPLD2 AOAH CEP57 AR PPP1R3G DDX3X LEXM SESN2 piR-61240-164 HNRNPL RPL3P2 CASP12 SH2D5 MIR30C1 E2F3 HDC KLHL3 SYTL3 ATP6V1B1 NOTCH1 ATPAF2 ENSG00000141979 ECD OCA2 CALML6 PNPO DAPK1 RPS26P11 CDH5 TDP2 PINX1 SELENBP1 NEK3 TNFRSF10D SSTR4 FOSL1 GSTT1 UGT1A3 ATG14 CD40LG CES1 ZNF259P1 TRAF3IP1 UGT1A5 SMARCE1 VRK1 CYP3A4 AGMX2 MBOAT2 GPAT3 SKA2 TRAP1 PYDC1 CENPM NPTXR SAR1B TTC7B LNCARSR GPC1 MIGA2 RBL1 GLS2 APP SLC51A CENPF C6 SNORD33 PRSS1 PTGIS PATL1 CEACAM3 LOC110599577 KRT23 PNKD SERHL UBC MIR33B ATRN HAPLN1 RANGAP1 NDUFB9 CNOT6L NADK2 S100A9 KCTD1 KCNA7 SRY CDCA8 CCND3P1 KCNK16 CEP250 MLC1 PYROXD2 IFT52 LOC110599575 PDHA1 SLC4A1 TKT BYSL PMPCA DDT RNF38 AHDC1 KLHL2 ZNF785 MPL GLTP DMP1 C9orf78 ADAM12 SLC52A1 RNU4-2 GAS5 PAK1 ADCY7 PPP1R14A GJD3 TPX2 ATP9B DAZAP1 HMGCS2 PRKRA TLN1 B9D1 MITF NKX2-5 KIRREL1 MIR608 GYS1 NSMCE4A IGF2BP3 DNAI1 FNBP1L MIR4632 HRG CLUH SLC33A1 TRIM33 FBXO42 HTN1 AATK LINC00665 RAB3IP NDUFAB1 ITLN2 MRPL45 BAK1 MTNR1B SPECC1L NSMCE2 TTC37 TET3 SPN PCSK1 SLC13A1 RPS5 ARF6 YEATS4 HOXA13 PCOLCE HMGN1 CXADR PCYT2 PUF60 SPPL2B ELOVL5 SHC1 TANGO2 BLOC1S2 GGT2 PUM3 S1PR2 LIMS2 ITM2B GINS1 PAPSS2 SMO ACSL3 MIR132 GLT8D2 UTRN CDC14A CNN1 KRT1 SDAD1 SIGLEC9 RPL35 IMPDH2 ENOX2 INTS6 CLTA SEC14L5 MIR320C2 ZNF702P IGH OGDH GAREM2 GPR37L1 GSS BISPR SIX5 TAF5 ANLN KCNA1 HSPA1L RHOC SSTR2 LYPD4 EPHX3 PPP3CA UTS2B SLC1A7 ALDH3A2 OXLD1 DDB2 CLEC1B ADGRE3 MKNK1 DYSF MTRNR2L4 MT-ND4L FOXJ1 MTRR SLC27A3 HTR7 PLEKHO1 RING1 BHMT NPC2 A2ML1 AVPR1B LMAN1L TRUB2 CEP170 PF4 MSH6 AIRE IPO4 QDPR FOXA2 SLCO2A1 MAPK12 PRDM16 FRMD5 SLC38A10 PDE6C EP400 PTK2 THAP12 MIR34B NDUFA8 LINC00323 LAMP3 FABP5 UGT1A10 SORT1 DPP9-AS1 PLIN2 LRIT1 YWHAQ SLC8B1 lnc-HLA-DQB1-1 SPPL3 RBFOX1 ENSG00000269807 KIR2DL4 MCU TICAM2 NFKB2 FZD9 RPLP1 GHET1 GNAT1 NKX6-1 IFT140 ALCAM ABHD2 FAM76B QPRT TRAM1 TRIM29 ATP1B4 RHEB SLC22A6 GPD1 NPM1 H4C13 PRPS2 GCK DNMT3B RPL29P8 ADRA2A CELF1 TERC TASP1 WDR19 EDRF1 TMEM92 MIR9-1 PDE4C CCT8 RAD18 WT1-AS FRA10AC1 LDHB BAMBI RAB5B MRGPRF TAF10 CRIPT DEPDC5 LTBP1 RYR1 AIR NDUFA2 FGF8 SENP7 LRRK2 FAAP24 CPEB1 RASSF5 IKZF3 TMEM14C EZR ATF1 FGFR3 DYNC1H1 HES7 TMEM33 POLDIP3 RPL32 POU6F1 BBS1 RGPD5 CDC6 PLD1 TP73-AS1 CHI3L1 VTCN1 ATP5PD NBR1 PKHD1 HPS4 TENT5C RARB UBXN7 DIRC1 MAFA MIR372 ULBP3 IMMT CEL CNOT6 DHX15 MARK3 HLF ARPC3 ADAMTS2 NPHS1 BRD8 APOBEC3B TCF4 NR2C2 LINC01508 NKTR MIR340 VSIG8 NAALADL2 FXN ASAH2 AURKA PAEP PNPLA6 SPG11 NOC3L AP3B1 AKR7A3 DNAAF1 COP1 CPAMD8 MLN TIAM1 MIR182 CDH24 PBX1 PXMP2 KRT17 FAM126B SYTL1 ZNF711 GGA1 VAMP4 BCAP29 DDX24 LOXL1 ALYREF ZNF275 NEK6 TAB1 LOC105943586 MIR181A2 PRRX1 SULT1A2 LDC1P MYOD1 GAA ZNF747 ARHGEF16 ACSF2 SCN1A KLRC1 IL1RL2 GDF9 MITD1 MED18 TRAF2 ECE1 BCL2L15 HCK FAM189B IL22RA2 WNT3A MIR29B1 ASAP2 YIPF6 GABBR1 PROX1 BMP6 SNORA49 LSM3 MOGS FMN1 SSRP1 ASXL2 RPE65 LINC00601 MIR485 SNAI1 SPCS3 CLDN9 CHAF1A DEFB4A PIEZO2 GC FTH1 MIR424 IER3 KCTD10 YWHAH MBL3P ADRB1 MUC5B FOXL1 CGB3 H2AC21 CSRP1 BOD1 UMPS C12orf65 COG4 NTM NFRKB FANCA DDX39A VTI1A GDF2 TIMM17A ZNF354C HNRNPA0 IKBKG FRMPD4 TM4SF20 PCBP1 SPOUT1 MAGEC1 NSD1 SEC13 GLDN EGF HAGH MIR7-1 CNDP2 FYN AGO2 TRIL GTF3C5 AKR1C4 KIR3DP1 DUSP14 AP4M1 ZNF649 ABCC2 CNGA2 SNX13 MIR614 CSPP1 NAIP OR1J2 XRCC4 AOC1 THY1 KIT TBC1D8 DRC7 EPHA7 SVIP MIR210 SCN4B SERTAD1 MARVELD3 CRCP TMPRSS13 CREB3 DGAT1 ETV7 HASPIN TAC3 RAD54B OR8K1 MED25 H3C6 BARD1 DGCR5 MIP F2 IL13RA1 WNT5A SPDL1 WIPI2 RANBP1 CSNK2B CFHR1 WBP11 TMEM176A IL31 CTNNB1 GALNT2 DYNC2I2 TMEM185B TERF1P5 MIR141 FCGR3A ANO10 CCNK FSHR ESRP2 CIBAR2 IMPACT WNK4 KRT83 GFUS ITGA6 GAD2 BMP15 CYP2A7 SLC7A6OS CA5A CCND1 USP35 SGK2 USP11 PIK3C2G PRKDC MSR1 CCL4 PDCD6IP NOL11 PSMD2 ZNF740 USP18 ATXN2 RRAS LAMC3 NET1 TOX IPO5 NPY1R MVD PCDH15 CHDH GCNT2 IK GABRG3 SURF4 BCS1L ACE2 KCTD17 SPSB2 GIGYF2 TPPP3 CILP2 CALB2 NARF-AS1 CD79B DNA2 CEBPZ ZNF479 FMOD IRS2 LUZP2 CLN6 ANAPC1 SIK2 SH2D3C PTPN14 TRIM42 APTX SNRPG RF00017-1860 BMS1 RPL17 MAGEA3 NFATC3 LRRC45 ADCK2 FASTKD2 ERC2 POFUT2 GNG2 HSD17B7 ENSG00000212161 ZNF354A DMGDH CAND2 RIMS1 SELENOS RCN1 SF3B2 LOC102724596 CPB1 BCL6 MDH2 BCAR3 POR SREBF2 TRUB1 ENPP2 MIR299 TGFB1 ALG11 ENSG00000257060 ZNF780B APEX1 CEP68 ZIC3 EOMES PAX2 MRPL11 LPAL2 VPS53 MPDU1 GAST UBE2L3 ADRA1D FZD10 ATP6V1E1 SCART1 SAR1A ASB1 KRT31 ZNF223 BIRC2 LMX1A NLGN3 MEP1A ENSG00000286088 TIMP1 PTPN9 UCK1 MRC1 MAVS KIAA0087 YES1 PWWP2A SDHAF1 ABT1 KRT78 IL13 PRNT PIP5K1C POU2F2 MT-TS1 NAA40 GPT CLIC1 PDK4 LILRA5 TREML2 NNMT ZNF74 SNX29 RPS2P6 EFCAB14-AS1 HYAL2 TBC1D10A IGFBP5 NRTN RBMY1D LOC109279841 EPHB1 CREB3L4 COLEC12 TNFRSF25 BMP10 SLC2A1 CREM PTGER4 METAP1 CCR7 KCNQ1 EPCAM RECQL5 NR2F2 CTTNBP2 ANGPTL1 ZBTB24 NLRX1 FHOD3 SLCO6A1 PRDM1 ARHGEF5 HAGLR FGD2 BIN1 SRRM1 PCSK1N PPP1CA ALS2 MOCS1 MIRLET7A1 NAP1L1 ECT2 DOCK6 CCR4 VWC2L DUSP23 CD14 SNAP29 HMGCR NUP35 WDR45B CDH3 IFT74 INSL6 NR3C2 IQGAP3 CRADD INPP5K LINC00921 DUSP12 CGREF1 BLNK PDZK1IP1 VASH2 IL19 COPB2 CTR9 VIL1 PTTG1IP CDC27 ZNF767P UBR1 COPS6 MCCC1 COL9A1 MIR129-1 VAC14 PSMD9 SOGA1 SRCAP KDM5B PPP1R13L PARP15 TTC19 GFPT1 MTRNR2L9 HMOX1 ARHGAP32 SLC27A6 MC4R EGFL8 FAM126A MRPS10 INS TRPM7 SERPINB8 FLT1 SLC25A2 PDIA2 NUCKS1 DIP2C-AS1 EXT1 HOTAIR CLEC4D RCOR1 EBI3 SMAD2 NXN ADA2 PPIH TSIX PRKCZ EIF5B IL20 NIBAN2 IFNA7 CRABP2 MYL12B ASL GATA3 AMY1A MIR135A1 ENSG00000217483 TFAM RALGAPB PARP12 KLHDC7A MIR301A GTF2H1 SRSF7 FLCN MPZ NAMPT SSBP3 FOXC2 HS3ST3B1 GDNF CTNS MMD2 PCLAF TEP1 GSTM1 KIDINS220 CRH PRSS2 CES3 ACP5 AMFR CA4 PLCB4 PGF CORT G3BP2 PYGB MIR214 TSC1 NARF DKK3 DDX31 TULP1 NHLRC2 AASS POSTN JAML TNRC6A APOL5 PLA2G1B RCHY1 RHOU CYP27B1 MARCHF5 VPREB1 ENSG00000255219 TACC3 SEM1 DMXL2 NAE1 IGF2-AS RBM14 PPP1R2P1 ENSG00000283782 ATG2A TNFSF11 RPGRIP1L ICMT PTPRF SEC24B DANCR ZNF808 TYK2 MUC6 ADAM28 NRL RAX2 GPAT4 PIP4K2A TCL1A MED7 CYP4F2 ARL4D SH3BP5 GPBAR1 AKAP6 MIRLET7B CTLA4 LOC108942766 SLC25A17 NECAP1 POLR2F PLA2G2D WNT2 GLMN CSGALNACT1 UQCC3 MCM7 TRIM52 DCLRE1A PDX1 PRPF31 TLR7 SMAD1 ACTRT2 ENSG00000249631 CLCN1 PDLIM4 SLC22A15 STX18 CCPG1 SLC2A6 PDGFA TRDMT1 IL17F ATP1A4 CFL1 IL4 RBP2 NAT2 PRG2 CPSF6 RPSAP18 TTC8 GBP1 PSMG1 CEP55 ZNF408 KRT20 BLCAP CEMIP HLA-DPB1 XIST GNE MGAT4C CTSE SLC35A2 TUSC3 GABRD TRPV4 SLC25A11 EHD4 NEK8 NOX5 NCKAP1L SULT1A3 SYNCRIP H4-16 ELOVL2 LOC110384692 OSBPL1A GOLGA2 TRA2A F2R ESCO1 PHF2 PID1 RFC1 SMIM31 MTAP ADORA3 MIR143 MCHR2 MAP2K7 GZMB HYAL4 TMBIM6 FCGR3B BMP1 DPF1 PATL2 SMPD3 DNAJC21 NFE2L1 SRD5A3 NT5DC2 HLA-G LRP2 MTDH HSP90AB1 ARSG ZNF483 PMAIP1 SLC25A20 S100A2 C2 NOP14 GCKR EIF4EBP1 PPP1R9B RGL2 TRPC6 FER piR-58016 ARPC1B PATE1 ENSG00000259602 SNRK ANKK1 UTP6 PIGY MIR652 DNM2 GLDC FBP1 PLA2G7 CCL1 TERT FUCA1 MIR106A CEP19 MFAP2 KCMF1 SOCS1 IL17RA DARS1 MPP5 SELENOI SPA17 CAMK4 CLPX LMNTD2 NPLOC4 PJA1 RB1 MT-ND5 IFNL4 ANKLE1 MPV17 PSMD4 SLC35D1 COL16A1 ERLIN1 JMJD4 COA7 GPX8 SLC29A1 GLRX TP53I11 SAA1 ALG8 ST3GAL4 PF4V1 REG1A FBXW7 CYB5R3 MIR30E NR0B2 TFAP2A RBFOX2 TIMELESS SLC25A36 MIR520B RAB11FIP1 PKD1L3 ZNF169 KRT7 YIF1A PPRC1 LOC109245078 PHF8 FGF16 CDK11B RABEPK LUC7L3 SNF8 ISX HAT1 RAD51AP1 HTR1D SGCD MAPRE1P1 IFT27 CA3 STX10 SEC62 KRT19 ZNF143 SBDS ENO1 CC2D2B P4HA1 MAN1B1 AKAP8L GAB3 DHRS4 COL4A1 RNASE1 NDUFB11 OSBPL2 STK19 SLC39A8 STS PRMT9 GLI2 BTNL2 TGIF1 SCO2 TP53 RAPGEF2 lnc-FCN1-2 MAP1LC3A ATP6V1G2 MNT OTUB1 ENTPD1 STK11 PRPSAP2 PRIMPOL MX1 PSMC5 EXOSC4 SETD1A EME1 SLN LOC107963950 RELB NLRP1 HOXA-AS2 KIR2DS1 LAMB2 HNF1B CTAG1B UMOD GRIN3B UPRT SRA1 ATP6V0A1 LOC110467515 ZNF615 GTF2I KCNN2 DYNC2LI1 DGKE RPS6KA1 MARS1 KDM1B SSPN KIRREL3 GORASP2 ADAM17 CHRNA3 MYOG SYCN CPS1 NCOA2 PIAS4 USP5 CFLAR WDR48 CDKN2C ACACB CUTC ABCB6 MIR150 FSBP FCGR1A DLC1 CERNA2 RPL11 SPRY4 RORC ETFB KCNH7 IFIH1 TEDC2 DENND2B TUBB4B WDR37 SEMA4A SNHG1 NUAK2 CERT1 AHSG RBM39 TIMM23B NKILA RGS17 PCTP SIRT1 TNFRSF6B MANBA GFRA1 HDGFL2 H3-3B UGT1A IL1B GAS6-AS1 HAX1 REN ZKSCAN3 PLEKHG1 CTSA CDKL5 COQ4 VGF BMX INPP4A PTPRU BRS3 ATP8B3 HOXC8 IL12B LARP4 CWC15 ZMPSTE24 TMEM107 PSMB3 NCAN RPS25 DNM1L ASMT EIF1AD CPT1C EXOC7 MPRIP TXN2 TOLLIP TEX29 TRAPPC3 TAF15 HAMP IDE DLK1 PLVAP NOD2 CERS6 GPR68 HTR2A POLR3GL GYG1 LOC106560211 LPIN1 KRT12 MT-TD TNP1 GAPDH ERRFI1 PEX12 APC CCN5 TLR2 LINC00673 SUCNR1 ZNF233 LOC110599570 GPX1 SLC39A1 PSAPL1 ADM5 LIMK1 CDC42EP1 MECR B4GALT5 ELF2P2 LRAT LOC109279247 MACROD2 IL18R1 BCL10 GLB1 TIMD4 MOCS2 HCRT CCDC7 REG3A SULT2B1 CYBRD1 PARP9 MMP10 TCN2 CDA ME1 TECPR1 IVL CHRNG CALM1 B4GALNT2 RCC1L CKM MAP3K11 ACOT8 CEBPE ESR2 AGTR2 DNAI2 CLASRP MIR513A1 EPM2AIP1 PZP RPS9 SIVA1 CD3E APRT GUSBP15 MIR198 PGLYRP3 GPS2 ATP5IF1 TP53RK KAT7 ITIH4 GATC ADARB2 PIM2 SLC17A5 PIPOX HGF EPHB4 PLAAT5 SOX18 SERPINA10 INSIG1 CPLANE2 REEP2 HSPA12A REPIN1 ARL8B CAPZB PDE4A SATB1 VPS45 PPM1D TP53BP2 GINS2 CYP1B1 GNPTG ROS1 BRWD1 ZFAT BMP8B SLC5A4 MT-TL1 SLC6A3 APC2 MIR210HG GOLPH3L SYAP1 SLC19A2 ZBTB17 C9 PVR ART5 LTA4H MAGEA4 ADGRE1 IFIT3 SLFN12L MYADM PDE3B TMPRSS11A L3MBTL1 GNRH2 STEAP1 PGK1 ACER1 CCL13 RLF PLOD1 MTFR1 GPAA1 TEX264 RYR3 VAMP2 CACTIN-AS1 XPNPEP2 RNF135 PDE6A SUPV3L1 NBAS FIBP SPIB DHCR24 MEF2D ZPLD1 MYO1B VAMP8 PANX1 FGL2 TMEM30CP WASL ADAMTS18 CYBA SOS1 MPZL2 PLAU MATK EHF CTNND2 PTEN MIR328 ANXA1 C1R SUSD5 SLC28A3 ERI1 KIR2DL2 SETD2 ROGDI TICAM1 RASSF3 UTP4 UGGT1 SERPINB5 TFDP3 RFX5 TXNDC15 PTPN18 NDUFA6 PKP1 HMG20B PTRHD1 SYNE2 NDUFAF3 RO60 SLC22A4 ETS1-AS1 NETO2 GALK1 SERPINA6 TRIM23 FBXL6 ARL1 FABP7 CDK5RAP2 PCAT29 FTHL17 KCTD15 LINC01419 CD1D PRAM1 NGF EIF3B AK3 DSCR8 APOA1-AS FLVCR1 COX14 PAX4 BIK NANOG ALOX5 CEP135 TRIM22 LYRM2 CDH13 MIR9-3 HES5 GJA1 IL6-AS1 FASN TAF7 PHLPP2 VEPH1 GK2 AMBP CD33 MRI1 CROCC GPX7 TGM2 BASP1 STAM MIR7-3HG ACTL6A UGT1A2P ARHGEF7 SLC23A2 MIR30A TBK1 STX2 PKD2 RPL29 MPPE1 NR1H3 CLCN6 SQSTM1 AADAC C3P1 BIN3 DEFA4 ENSG00000273886 ZNF197 PTOV1 GABPB2 ENSG00000279159 PTPN11 CMIP ATF3 TRDN UCHL1 CDR1-AS PLK1 NPHP1 NDUFA11 DAB1 H2AW PUSL1 ZDHHC16 ANXA13 ATP8B2 DDX47 BTG1 DPP4 APOC3 NPPB ZNF148 TLCD4-RWDD3 IKZF1 ZNF141 PSMC2 APEH G6PR GGA3 KCNA2 LPGAT1 PCYT1A BRMS1 LINC00173 CYP21A1P SEC16B THOC2 LGALS1 CASP10 ALK COL15A1 DACT3 EXOC6B PCMT1 VAMP1 SRI HDAC5 HLA-E SLC25A32 EVC2 FLT3LG ATP1B1 WDR1 SGPL1 C5orf15 SWAP70 TMEM222 TRIM31 LILRB4 GSTA4 ARNT UBA7 MIR320B2 CDKN1B FOXC1 LSM14A BTRC IQCH STEAP3 CABP5 YEATS2 TRIM3 MIR454 FGG ABCA1 JTB CLEC2B PQBP1 THBS3 REST MIR433 MGAT5 TSPAN8 MUC1 DYNLT1 MIR498 HLA-W TEAD3 COPG1 C3AR1 CADM2 TENT4B HAO1 TWF1 MACO1 MYH9 LBP CGB5 MIR196B BDKRB2 HSD17B4 IDH2 FSCN3 AICDA ENSG00000267484 QARS1 DCLK1 MEF2A NOP2 ZNF836 ASF1B HTN3 COPZ1 ADAMTS9 SLC4A3 CDKN2B-AS1 STIM1 TBX2 TMEM237 RPS4X IFNG-AS1 IFITM5 SYPL2 TRMT10B RPS3 RAI14 ADCY5 MROH8 SGCB FRAT1 LOC111832671 DUOX2 APMAP PRKD3 CASP8 CFH NRIP1 TRO BNIP2 ZNF707 DHX30 TBC1D22B EEFSEC CASC3 MIR203A KRT3 ARF1 MAU2 MOG NOTUM ATP7A ERAP1 PLCH2 CCNG2 MIR137 GIP HOXB13 SPAM1 PPP2R1B APLNR JAG1 CHGA ST2 CACNA1B MMAB PAPPA RAPGEFL1 MIR494 RHOA TRH DCTN3 NT5C SLC1A5 CALCA MT1M ANKS6 CXCL11 DTX1 AKR1C2 TNNC1 GFI1B KY945953 RF00017-3608 SUN1 LOC112081413 ASB3 CASD1 ZNF527 TTBK2 TRIM43 FOXO6 HLA-DQB1 SCRG1 CBSL NRAS RPL22L1 FBXW2 SIX3 DUSP26 HDAC9 OGG1 ZDHHC24 DNAJC3 LITAF RPL15P15 AFP CLDN24 TUBA1B CRY1 MYT1 VILL HECTD4 TAMM41 CHST13 ZBTB43 VAPA PIK3R3 STAP1 SLC34A1 DLX6 FBXO40 BRF1 CCL27 MIR297 EMP1 MYBPH COG2 PPP1CC CDCA7L MTOR ENSG00000235610 NR2C2AP MIR124-1 RPS2 C17orf75 SLC2A8 WT1 CARD6 LEPROT IL13RA2 TXNRD2 AOC3 TNFRSF4 MTHFD2 KSR1 C4B_2 SLC27A1 OAS3 SLC22A11 STARD3 LIMA1 CTAGE6 C2CD4C GABRB1 FBLN1 GSTO2 MRRF MTFMT PRKAB2 ZNF76 AFF4 HSPB2 RAF1 INF2 ZIC1 LYST KLRK1 UBE2M SLC16A9 ARL10 NCR3 LTB4R2 ST8SIA1 HLA-DPA1 FKBPL lnc-SMAD2-3 CD248 SNX4 CCR2 H4C2 SH3KBP1 SYNRG DRD2 KRT76 COPS2 CEACAM4 CFHR4 GATAD1 FCER1A LMOD1 CALR HIGD1A ADRA1B SNRPD1 LTB NEU3 DES BZW1 ITGAV CCDC158 ZNF221 PCNA RBBP4 MMACHC PHKA1 PRKAR1A HSD3B1 ATG5 ZRANB1 lnc-HLA-DQA1-9 RAD52 ASIC1 CD207 RSPO1 SLC38A8 GM2A MYCT1 SCGN ETV4 CD2AP CCL20 ZFP30 CCDC86 SMN2 MYH10 CTBP1 OR10J1 DNAJA1 MIR103A1 ARG1 KIF5A ASCC2 KIF2C GFM1 OPTC FCRL2 SNRPEP4 HBD PCK1 BABAM1 MYO5A CFC1 IRAK3 SIPA1L3 MEGF8 ADAM10 LIPA GALC MRPS22 GNG4 TBKBP1 CAMLG PPP2R2C TREML4 P2RY12 PNPLA1 KANSL1 FAM53C VWF BAD VTN PYHIN1 CIDEC CRIM1 SEPTIN9 FLNB KDM8 NOC2L SCAI ENSG00000173867 MT-TW CRTC2 IL15 LAT2 ANGPTL8 SLCO1A2 SUGP1 LIG4 ECSIT BMP4 SIN3A SYCE1 VSIG4 CFAP58-DT TXNL1 MC1R ISG20L2 CCNYL1 RNF115 CISD2 OBSCN GBA SLC52A3 RNU6-440P CYYR1 ESD UBXN2B PRDX5 PPIA VDAC1 CLDN17 TRIB1 NT5C1B TXNDC5 CD46 ICOSLG DSG1 SEMA6A-AS1 CCK PRAP1 DEF6 HJV TYSND1 UTP14A RPH3AL NXF1 NHEJ1 SEPTIN2 TRERF1 MTBP TUBB3 SLC24A2 NELFB SEC22B S100A6 CDKL2 NEDD1 EPC1 TINF2 THRSP CYP4A11 SCEL MIR3714 EPRS1 SH3TC1 C1QTNF9 ILF3 GOPC PNPLA8 NTRK3 SPDYE6 LARP6 FMO1 POLR3C MIAT SGCG FBN1 C12orf57 DCDC2 HAUS8 NANP SNX14 ATG101 INHBB TBL2 ADRM1 ATP5ME PEX6 TXLNA ENSG00000266990 AIP SLC6A14 TNFRSF13C DIPK2B CEP89 AIMP2 ZEB2-AS1 LAMA5 MIR133A1 HNRNPH3 AKAP4 DIS3L2 FREM2 ARNTL DROSHA CTCF CEP43 SNTB2 CCL2 PUS10 KPNA1 KRT81 ALDH3B2 MOGAT2 AGAP2 M6PR CCND3 COASY LAMC1 S100A11 CLTC EFHC2 DOT1L ZNF521 EIF2AK3 COL13A1 CHMP4B TMPRSS2 SIGLEC6 ZNF629 CCDC93 ERVW-1 KCNK4 FAM86B2 ECI2 CMAHP ANGPT1 SNAPIN RAN MED23 UFC1 EEF1A1 PSMD14 KDM3B HMGA2 MIR15B EPHA1 GAR1 EIF4G1 INTS3 TRPM4 FAM186A ISYNA1 UBE2D1 RAB39B IDH3A LSM7 CREB5 ANGPT2 DYRK1B HLA-DRB6 EEF1AKNMT SLC4A10 ACSS2 FGFRL1 COPS7B GPR89B BAG6 LINC00844 ITGA4 RIN1 PHLDB1 CLOCK MLX NSUN2 LGALSL MIR18B PSMA4 RILPL2 KIFC3 ROCK1 H4C9 IFNL2 TAGLN TPSAB1 ZNF45 ZNF610 TK1 MIR320D2 ZDHHC18 SNHG3 ARHGAP31 TMBIM4 KLF1 NMUR1 KIR2DS4 TMEM51-AS1 KDR KCNK2 DNM3 RLN2 GIT1 MSL2 HPD PDGFRL SMYD4 ODF2 TREX2 MFAP4 SLC25A47 EPB41 GZMH NUP98 ENSG00000219410 RNF220 TRABD SHCBP1 COTL1 CLDN15 TNC ITGB1BP1 BRAP SESN3 SOCS7 MARCKS FOXO3B SACS RRP8 MIR145 SH2D3A PPARA DVL3 KRIT1 LEFTY2 RORA PTPRD PIAS2 CYP4X1 TRAPPC12 CRX PLK2 ARHGAP9 UGT1A9 PRIM1 PAIP1P1 CRYAA ATP1A2 BACE1 PLEK2 ENSG00000255495 TG OPTN HAR1B MAPKAPK5 ACAD9 ZFP91 RBP4 RNU6-201P METTL9 TFB2M SP100 CPSF1 IFNA17 S100A4 LIME1 BTC MAP2K5 NUP188 RPS20 RAI1 LAMB1 IGF2 ATAD2 PROK1 PSC RPS10P7 NPEPPS EPN3 CD80 DDRGK1 CLIC3 PHF6 HMGB4 HTR1B DNMT1 EPB41L2 JMJD6 MIR486-1 WIPF2 ZNF283 SBF2 PPM1B MMP21 SUV39H1 SLC7A5 GZMA AAMP NFIB MBD6 LINC02875 CAPRIN1 ACKR1 ETFDH ZNF23 SLC15A1 UQCC2 LRCH2 GSPT2 NAT9 MB LIFR ENSG00000253432 CACNG8 CYP4Z1 DMBT1 KCNAB2 EIF4A1 IL3RA NLRP2 MAP3K2 IFNK PCDH19 KMT5B TSPO LEPROTL1 ADORA1 SYMPK RTTN POLE2 IL2 ARRDC3 STX12 NIN DDIT3 HPCAL1 CDH1 PTGR1 ITPA EDA CNTRL SAP18 IFIT1 HSPA5 FNDC5 JUNB WNT7A H6PD RALA CAD SAP30 XPA MGME1 SPDEF APOBEC3H ETHE1 GNPTAB ABCC10 IRF5 ACAT2 GNMT RPAIN IL36A SRSF1 RPA1 PPP4R1L RAB29 EEF1E1 WNT10A ANAPC16 MICB RDH12 CELP METRNL C19orf48 IAH1 ZNF346 NCR1 CD52 KHDRBS3 SLC30A10 MICAL2 UBA52 PPIF RIPK1 COL14A1 ATP8A1 PARP10 ANKRD50 SMU1P1 CSAD CXCL9 RECK ABAT SLC22A25 H4C15 VPREB3 RAB3IL1 FGF18 EIF2A GBP6 CRBN SPINT1 ATPAF1 SNORD59A PCDH10 SLC9A1 CYTOR SSPOP RF00026-678 DNAJC28 IGLON5 CTSL NEXN RNU6-497P ELSPBP1 SRPRB MACROH2A1 FOXF1 MIR2392 LTF TPO MRPS27 UBAP2L MLST8 TTYH2 GATA5 AGRP ENSG00000272668 SMOC1 ADAT3 MOB1A MIR100 RCE1 CD81 MIR155HG MYH7 KCNU1 CYP2W1 SH2B3 KIF1C C19orf33 PAPSS1 BICC1 MIRLET7F1 ELF2 ADAP1 PTCHD4 AP3D1 KCNK13 MTCO2P22 RPL27A SLFN13 CCDC169 GLYAT OGFR MC5R TGFA MMP17 MIR194-1 MYL5 PRLR CHIA MED28 CEACAM7 PTPRA INMT GOLIM4 HSF1 ADSL LAS1L BAP1 UBXN1 HOXA9 TMEM161A LNX2 METTL7A HIF1AN POLR2H ITM2A ABCG5 VLDLR-AS1 DSG3 ZNF180 MIR16-1 BUB1B NFKBIB USF1 JMJD1C ACP3 SLC46A1 PRRC2A DKK1 AAGAB CAPN5 KCNH5 NDUFAF5 MAGEA1 PA2G4 NEDD8 SARS1 MIR139 ABHD11 POLR3H NOTCH4 CADM1 INPP5D RILP PAIP2B HJURP KCNRG MAT1A C6orf136 CALM3 MIR29C TSPAN1 ACIN1 NMI RGS9 B4GALT7 CNKSR1 CEP41 MS4A7 SDHC ATF6 EYS GBF1 UBTD2 RNASEH2A DEPTOR EIF2AK1 CCR10 B4GALT3 TXNDC11 MAP1B HLA-L LTO1 ATAT1 ARC PPFIBP2 NUP54 VCAM1 STAT2 MIR19B1 PTAFR ALG3 REL RNF40 EWSR1 CR2 GNA14 CSN1S1 ALMS1P1 IFT81 CPTP CYTH3 PEX10 PLEKHA8 MLEC KLC1 GALE NT5C2 AKAP13 FLG IFNA1 ZNF337 HHIP GXYLT1 ADH1B MIR146B DLD LOC106627982 DNAJB12 PROX1-AS1 MLANA HMMR PKM RPS7 JAK3 HEY2 GCG FCER2 ARFGEF1 RPL34P5 CXCL5 ARHGDIA AP1M2 GCAT HSALNG0128250 LAPTM5 CETN2 ZNF286B NOLC1 IARS2 TKFC CHERP FSTL3 PEX11A FCN3 NPTX1 PTPN3 PLD3 ART3 FOXP1 CLEC4G ENSG00000268938 SLC5A1 MLNR POFUT1 C16orf95 MYO1G RGMA KLHL17 TJP2 KRT10 SERINC1 LILRA3 CCT5 SLC9A8 RAB27A PEX19 CNP EDN2 PSMD7 TM2D1 LRP4 PPP1R3C HLA-DQB2 CPS1-IT1 HLA-DPA3 SLC5A2 ELP1 C4BPB ORC6 CCL25 ARL15 POMT2 SOX11 CCN3 HIVEP3 CGN EIF1AXP1 PKD2L1 BMPR1B GATA6-AS1 SYVN1 PDE8B LPCAT3 LTC4S PSMC3 SMARCA2 MIF4GD TUBGCP6 P4HB ADH6 MTRNR2L7 TLL1 LOC105372509 CLDND2 ALKBH8 CST2 ENSG00000223944 ENSG00000269191 BRF2 SIGLEC11 DEFB103B BCL11A RAMP2 F11R STAC3 RFX4 FAM193B CLUAP1 LINC01234 PSMB1 VIPR1 TXN EXOSC6 ACTA2 FANCE BECN1 GMPS LGALS8 GPT2 ANKRD1 DDR1 ATP6V1D ALDOB PTGS1 TACO1 ZNF112 H3-4 RBMY1B SNRNP40 UBE3C TRIT1 LRP2BP PDSS2 MEIOC ATE1 HES4 ARAF KLF10 NORAD ASCL1 CLEC4F lnc-TFAP2A-12 HKDC1 PSME3 IFNA10 MIR1307 REX1BD CYP46A1 PVT1 L1TD1 NMD3 CHN1 MIR1205 RAG2 HGSNAT PMM2 EMILIN3 GATAD2B VPS13C METTL3 ANXA4 CA1 UAP1 KCNJ15 DHODH MIR186 TULP3 RPS14 ATP2A2 ATIC APBB1IP CXCL16 ONECUT2 ATOH7 NPL GAB1 SULT1B1 SNAI2 KCNN4 GLI4 UBE2Q1 JPH3 MIR520A C20orf204 GPR39 CYP2J2 PXK APOL3 FLNA IL2RB BAX LRPAP1 IGHE CP LINC01956 G6PC3 LOC110283621 GORASP1 FKBP1A POC1B CYP24A1 SMARCAL1 NDUFS7 ABCB8 PRMT7 COQ2 AHSP USH1C SRF DNAH5 MAT2A MAL2 TUBB4A PGPEP1 SIN3B MIR202 KLRD1 TRIM8 NDRG3 RNF6 SEMA3C VAV1 EVX2 PDGFC PEPD TP63 JCHAIN ANXA11 IFI44 RPS6KA3 RETREG1 PHF21A MIR212 DACT1 PNPLA2 PAH ERCC2 PTCH2 TRMU CCDC9 VPS13B DCLRE1C PSMB9 CYP2R1 HCLS1 GPR15 CSF2 SLC2A11 MMP9 GPLD1 RAB8A DHRS2 RPS27 SNRPD2 AQP7 CTSC |

| **Table 3** A total of 1768 known therapeutic targets for psoriasis were collected form the DisGeNET database. |
| --- |
| CERNA3 RIT2 HPSE2 CHPT1 CD44 MMP2 RPTOR BCL2L12 PINK1 MT1B ZAP70 DNASE1 RPL17-C18orf32 GRAP2 SPINK1 MEFV CNBP SLC4A1 VPS4A DHCR24 HSPB1 ABCC6 PMPCA CXCR4 DECR1 BID MIR26B GLTP CREST1 EIF6 IRAK1 CYBA IGF1 MIR146A SOS1 MIR20A G3BP1 TGFB2 GAS5 PLAU RAB1B MATK MIRLET7G IFT122 JPT1 HSPB8 SUMO1 MNAT1 DDB1 PTEN ANXA1 GDF15 SIDT1 AGFG1 TPX2 HMGCS2 SLC28A3 EP300 TRIO NTSR1 RENBP RTN1 KIR2DL2 ERVK-7 NOS2 TICAM1 UGGT1 TRIB3 BTLA PACSIN2 SLC6A4 CTNNBL1 SOD2 HCP5 PPP1R13B TOP3A MSH3 HLA-DRB4 SEMA4D MET TUG1 GABARAP IRF1 GOLM1 PTK2B GOT1 CD1D UNC5A PLAAT4 MBL2 RAB40B CECR SH2D1A FLVCR1 UGT1A1 PI4KB ZFYVE1 MIR200B CXCL13 B3GAT1 NANOG PLEK NAT10 TRIM22 NLRP3 LEP HEIH DDOST FASN CCL22 KIR2DS3 CD33 ILF2 PTPN22 AKR1B10 BRCA1 GGT2 BMP3 DIAPH2 CYP2D6 IGKV3-20 ACVRL1 USE1 CRY2 RUNX3 TGM2 FCGR2B LAMP1 MIR7-3HG CLDN4 RXRA PDLIM7 ARHGEF7 MIR30A CYP2C19 H3P8 TBK1 ACSL3 STX2 PRL GLT8D2 SLCO2B1 NR1H3 RMDN2 ACACA IMPDH2 EPHA2 CRLS1 ELANE CREB3L3 MIR98 SQSTM1 CCL4L2 FEV MIR149 CD55 IGH PDIA3 ACAT1 MTHFR ERVK-24 PTPN11 SMAD3 LNPEP MIR511 UCHL1 FOXP3 HMOX2 SMYD3 IFNL1 RSS CES2 NEIL1 CAVIN3 EGOT FBN2 ARHGEF9 GPR55 PPIL1 MIR148A RAD23B MIR204 DPP4 NPPB APOC3 CD38 MIR19A TNFRSF9 LAIR1 SPG16 LAMTOR2 IFNA2 PCYT1A MAPKAP1 BTF3P11 LGALS1 SLC25A1 APOE PF4 PIAS3 RBM24 HLA-E ALDH2 DAPK3 C3 FAS PPP2R3B GRB2 BTN3A2 MCS+9.7 PON1 FOXA2 POLE3 ATP4A UBA7 PI4KA CDK1 MIR99A CDKN1B INSRR APOC4 FOXC1 MIR192 LOC102724971 NELFE MIR34B HSPB3 SHBG NM ABCA1 MIR223 LAMP3 MIR200C TNFSF10 PLIN2 PREP CD82 BCHE SERPING1 CKB RTN3 MUC1 PPM1A TICAM2 ULK1 SLC26A3 NFKB2 BCL2 PART1 ADI1 TNFRSF10A FAM72B SLC40A1 TNFRSF11B MIR27A GOLPH3 EFNA4 CD36 QPRT IL24 RACGAP1 ARF4 PSMD3 PIK3CB RHEB LBP MBTPS1 MIR215 COX8A SF3B6 MIR196B TOMM34 NPM1 XRCC6 CARD8 AICDA PCK2 XRN2 PIK3C3 DNMT3B DCLK1 TLR6 CELF1 SMIM10L2B TERC BCL9 MRPL28 CNOT1 EDNRA DDX18 ABO CDK2AP2 CXCR2 TFR2 PLXNB1 ERVK-19 RPS4X SND1 F9 GHRL LIPE IRS1 MFN2 BCAP31 RPS27A MIRLET7C CYP1A1 STAT4 IRF3 CD40 IGFALS E2F1 DEPDC5 DPYD ARHGEF2 PTGES3 LIF CASP8 APOB OXTR SDC2 TAP1 SUV39H2 NRP2 IGFBP7 H3P36 ACP2 NCAM1 DYNC1H1 LAG3 ARF1 AGT KRAS ERAP1 POLDIP3 ODR4 CAVIN1 IL6R MIR885 PPARG MERTK CHI3L1 CD300A DHX16 MIR147B HSPA4 CLDN6 MIR494 CD99 CRP DCTN3 HSPA8 TP73 MIR619 IMMT CEL CBFA2T2 ENAM CXCL11 SELENOP SERPINA1 HLF PPIG CRHR2 RPSA TAPBP PRKAA1 GSTP1 PKIB CCR3 APOBEC3B KIR3DL2 ATP2C1 OSBP CYP4F3 HLA-DQB1 STAT5A AHCY NRAS MTTP AURKA CXCL8 SRD5A2 FKBP4 MIR122 CCL17 PTPN2 PAEP CLDN7 CD58 OGG1 HDAC9 AP3B1 GLP1R FUBP1 DNAJC3 AHI1 DYM ZNF410 COP1 STON1 IFNL3 RPP14 AFP MIR3648-1 IL18BP TUBA1B MIR182 CRY1 CYCSP25 FN1 KLRC2 CNR1 ABCB1 KIAA1549L AHR ALPP TRAT1 TPMT VAPA NFE2L2 IGSF8 IL23R KLF6 NR1I3 PRIM2 YWHAZ FCN2 PRH1 CLEC4C DNALI1 ARMH1 IFI27 KMT5AP1 ARNTL2 HDAC3 HP KLRC1 CCL27 IL1RL2 IL17A TRAF2 AURKB TM7SF2 ZC3HAV1 WNT3A GOT2 LEPR MIR29B1 LTBP2 MTOR PDR MTCO2P12 BMP6 SFRP2 MCM3 VPS51 DCX CDSN PRH2 CYP2A6 CDKN2D TBX21 PPARGC1A TNFAIP6 RTEL1 PGLYRP2 KDM1A ETFA GP6 BRD4 POLRMT NDUFA13 SLC22A1 MAPK14 AOC3 MTG1 CYP3A5 TPT1 PLA1A CRABP1 CBLB GGTLC4P CCN2 AP2M1 GC FTH1 IER3 OAS3 ANXA6 PITX1 MBL3P MIR208B IL17D VAPB CSK STARD3 RNA28SN5 GPR158 EIF2S3 H1-5 CTSS CTSB JUND OAS1 PHB2 PXN ZC3H12A TNF LCK HSPB2 DDX39A C1QBP HSP90B2P MIR29B2 RAF1 VTI1A TIGIT SUB1 KLRK1 IL23A NCR3 HLA-DPA1 EGF IL1A TMED7 GGTLC3 CDR3 CCR2 CAV3 AGO2 UPK3B SMOX ITGB1 LDLR CCL14 SMAD7 GGPS1 VLDLR LARP1 SDC4 ABCC2 HPS1 MPO RNF7 VIM SNRPD1 PARTICL TLR8 HLA-DOA LGALS3BP ABCC3 XRCC3 IRF7 SET PCNA CSF3 MAP1LC3B KIT CSNK1G2 HLA-A ATG5 KRT8 ZGLP1 THPO EDNRB UCA1 CD27 FHIT EIF2S1 SCD H3P11 PRB2 CHUK CREB3 TERF2 DGAT1 RAB5A CD226 TNFRSF1A STAT3 CASP1 CD8A DCAF1 PLIN3 ATG7 HTC2 CD2AP CCL20 ROBO1 ATN1 SMN2 SPP1 HBP1 IL22 PLA2G4C LINC02605 PHGDH MIP F2 APAF1 AMPD1 WNT5A MIR491 HSF4 APOA2 TNK2 ERVK-10 IFNGR2 IFNAR1 H3P13 LGALS4 PTGS2 ERVK-32 B2M TBC1D20 ARG1 CAP1 ASCC2 DUSP1 YTHDC2 SLPI STX7 SULT2A1 RMDN3 CTNNB1 TRIM27 HPGDS LIPA MIR221 STING1 MICA PAK3 TRIM56 UBE2J1 HNRNPK ELF1 TGFBR1 FCGR3A CLIP2 PRDM6 CYP2C9 LINC01194 LOC107987479 NOX4 FCAR CDC42 VWF UTS2R VTN IMPACT LRPPRC IL18 CYP2A7 CCND1 CYP2E1 USP11 NOTCH2 FLNB H3P19 SIGLEC1 KLRG1 CCL4 NEURL3 USP18 WAS RRAS MYO18A CRTC2 S100B CLEC4M BMP2 IL15 TOX ATM ESR1 PGGT1B SURF4 SST SLCO1A2 FKBP5 SEMA3B-AS1 LIG4 PRF1 CDK4 TMED5 SPSB2 SLC12A9 TRIM69 IRS2 FAM107B SPECC1 KAT8 SDK2 PPIB IL15RA RPL17 BMS1 MAGEA3 ERVK-20 SNCA GPX2 CD9 ALDH7A1 CSNK1D CCL4L1 PPIA CASP7 TJP1 KIR3DL1 ASRGL1 NR1D2 ARHGAP26 GAP43 HRAS PTX3 PER2 MIR216B TMED2 NTPCR CINP PRSS3 HJV SMAD4 CASC2 CDKN2B BCL6 DHCR7 TBP AFM NXF1 PSMB8 SREBF2 VEGFA XRCC1 STAT6 TRIM14 THOP1 ENPP2 PER3 RIOK3 PIK3CD NDC80 SCARB1 NRSN1 GCHFR PPP1R12C TGFB1 ITGA2 GLI3 MME APEX1 S100A1 CAVIN2 TFRC LPAL2 DDX46 SPATA2 MYC SGSM3 PELI1 TNFRSF8 H3P23 ILF3 ULBP1 SOCS3 UBE2B CCNA2 GOSR1 RAB7B IL10RB DCDC2 TMEM132A TIMP1 HAVCR1 CSNK1A1 GP2 CHIT1 HARS1 TMED10P1 CPQ MRC1 MAVS VCP PCSK9 AIF1 SOAT1 MR1 ARID2 SLC14A2 SMG1 IL13 CD86 AIMP2 LIX1 IGHV1-3 CALM2 MIR21 KIF1B GSK3B ATF4 PTF1A H3P20 OASL ARNTL IL12A ALAS1 BMPER FOXO1 ERVK-8 GPT SIGMAR1 IGAN1 MCL1 CRLF3 SMIM1 CCL2 APOM KPNA1 FUT8 MIR125A PRNP AGA SPATA16 PDIK1L NUDT3 CREST2 CRISP3 PIAS1 MMP1 ADA STAT1 CLTC IL27 HLA-DQA1 EIF2AK3 SRSF4 GABPA POU5F1P3 DCTN6 TLR3 IL6 OR10A4 NXT1 ERVW-1 FBXL2 PTBP1 PTPN7 MARCHF2 TBPL2 TNFRSF25 CDCA5 STMN1 ANGPT1 CARD16 HABP2 MIR758 CPT2 ATF7IP SEMA6A CREM LSAMP CCR7 CD68 EPCAM MVP WEE1 EIF4G1 BCL2A1 HHAT MIR185 ING2 NLRX1 HNRNPC SRL CR1 ARHGEF5 SLCO6A1 HMBS EBP GADD45B H3P17 MIR152 ISG15 ISYNA1 TDP1 CASP3 HSP90AA1 ZFYVE9 RERE ANGPT2 SEL1L PROC NAP1L1 HSD17B13 MIR335 FPR1 FKBP8 PLIN1 DHX9 PTPRC CXCR5 SMOC2 CD14 PKN2 VDR CLOCK ACE PPARD HMGCR NSUN2 AGO1 HFM1 HACD1 ANXA2 MCF2L OPN1SW ITGAM DUSP11 CD34 IFNL2 HDDC2 DDX19A MIR134 SHC2 PDPN MIR942 INPPL1 IL19 RTN4 FASLG ENG MIR126 HLA-F KIR2DS4 GGT1 DCTN4 IVNS1ABP CD163 EIF2AK2 WNT1 AP1S3 VAC14 UCKL1 ERVK-25 PSMD9 IDO1 KRT14 KDM5B OGT SYT1 LUM ABCG2 CAMKMT IL1RAPL2 UBASH3B HMOX1 PPP1R15A FCGR2A PRKN HNF1A EPHA3 FLT1 GZMH NS2 IFNE RARA FUT1 DFFA HNRNPD NEIL2 EXT1 HOTAIR EIF4E PECAM1 MS4A1 MIXL1 TNC MYD88 SMAD2 MIR501 PML LOC110806263 UROD IFITM3 EIF5B WDR11 IL20 EMB BTN1A1 MIR636 TRAF6 SIX6 ASPM MIR145 CCR6 AXL UBE2S CRABP2 PPARA PNPLA3 RACK1 CNR2 CD244 CBLIF KRIT1 MIR30B SLC10A1 PMEL RORA PIAS2 IL2RA CD24 PIK3C2B MIR301A FAP LDLRAP1 LY75-CD302 CCR1 CRYGD USP15 SLC12A3 GTF2H1 ITGA2B BST2 CYP1A2 TNFSF4 MIR34A OPTN RPS6 DUSP6 MAPKAPK5 ERVK-6 SLC28A2 MIR222 PCLAF BMPR2 TRPC5 RBP4 KLHL1 IRAK2 AHSA1 MFGE8 MIR1231 GSTM1 IFI6 KIDINS220 IL10 MAPK1 LOXL2 IGF2 PGF NFKBIL1 SAG CD80 SLC30A8 ACR EFL1 TULP1 DNMT1 TNRC6A PLA2G1B FTO TRIM21 CYP27B1 OCLN RBM45 KIR2DL3 POTEF FABP1 RNASE3 MMP14 PIK3CG ACKR1 MDM2 SORBS1 EIF3A PEA15 LPA LY75 TAP2 IL37 NPC1L1 BLVRA AKT2 EGFR TAT IGK TYK2 NQO1 NDRG1 SP1 SEC24C KIR2DL5B GTF2A1L TNPO1 FARP2 CMC2 MYLK MAPKAPK3 SOD1 RAB6A EIF4A1 NCAM2 MIR200A GSTK1 MIR130A CLCN5 CXCR3 IL1R2 PIK3CA H3P38 GPR161 SERPINB4 IRGM TSPO MIRLET7B CTLA4 HTT IL2 TBPL1 MCM7 ERBB2 APOH CREB1 CYP2C18 SDC1 HNRNPDL SYK SMAD1 TLR7 RMDN1 IGFBP1 IFIT5 DDIT3 MIR196A1 LGALS3 HPCAL1 POLDIP2 UGT1A7 CDH1 ITPA NAAA IFNG FOS CYP2D7 IFIT1 HSPA5 RETN IL17F SERPINB6 JUNB CCL8 TNFSF13B IL4 CYP27A1 CD28 TGFBRAP1 MASP2 HTR6 BRD7 CAD AGL GBP1 SLC7A9 IGHV1-69 CCL3L1 KRT20 CCNG1 CMD1B HLA-DPB1 KIR2DS2 STAT5B CLU IRF5 ACAT2 SSB CXCL12 DDX6 MIR93 ATF2 NTN1 GUCY1B1 SLC35A2 OAS2 RN7SL263P SPRR2A MIR224 RAB29 FCGR2C HM13 RRM2 HK2 MICB CD63 EPHX1 DNAJC5B NCR1 TOP2A CX3CL1 AIMP1 TACSTD2 MAPK8 SLC27A4 KLRC4-KLRK1 HDAC8 KIR3DS1 OSBPL1A IFNA13 ICAM1 RECK HLA-DQA2 CXCL9 F2R CAV1 THBS1 MAFD2 DGAT2 BCL2L1 CD19 RNASEL TNFRSF1B EIF2A LGALS9 MIR136 PIK3R2 MAPK3 SPINT1 MAP2K7 GZMB TNFRSF21 GGTLC5P FCGR3B AKR1B1 LOC102724197 ABCD1 HLA-G CA2 NRDC TPO LTF IL6ST MTDH MYDGF CRYZ ARF5 SLC3A2 SLC25A20 CABIN1 MYCN ISG20 AGRP HSP90B1 APCS NCL GCKR EIF4EBP1 PSMD10 ABCB4 SMOC1 CXCL1 GUSB CD81 HNF4A WDHD1 EHMT1 TNFRSF10B FBP1 H3P24 CDK5R1 ADH4 TERT MIR503 MCM2 BTN3A3 KRT18 RELA ERVK-18 TMED7-TICAM2 FAM72A SOCS1 MIR155 MIR1247 RAB32 EDEM1 PTH NOS3 TGFA FBL PROM1 MIR101-1 ID2 ADH1A ROBO3 STAU1 RB1 FCRL4 APOC2 IGHG3 IFNL4 GGCT PTPN6 ADAR MPIG6B IGHV4-59 WRN BAP1 ERLIN1 IFNLR1 NUMB MIR25 TNRC6C SLC29A1 MSH2 SAA1 ST14 REG1A H3P28 FBXW7 NCKIPSD SRC XBP1 ITGAX CEBPA MED9 NR0B2 XRN1 NFKBIB IRF2 USF1 TIMELESS TENT2 PLIN5 DKK1 CAPN5 PPP2CA TMED10 IL1RN SEC14L2 ODC1 KRT7 SDC3 SALL4 NOTCH4 H3P9 CDK11B RILP CD5 RABEPK SNF8 RAD51AP1 SYBU GFAP OPRM1 CYP17A1 ACTB IL7R CALM3 IFNA4 GFER APCDD1 CD209 TLR4 KRT19 PCBD1 SDHC CXCL10 CCL21 GBF1 ADAMTS13 IL16 UBE2Z ZNF185 AZIN1 IL10RA IL36RN SERPINE1 HFE RNASE1 VCAN HERC5 SMN1 POU5F1P4 MKI67 BACH1 TSLP STS ICAM3 NHS MIR17 TAF8 STAT2 RNF139 CCL5 TP53 IFNGR1 CR2 RNH1 CSNK1G3 MAP1LC3A ATP6V1G2 ERVK-11 SLC29A2 MIR373 COX2 REXO1L1P RSAD2 IGHV3-52 OSBP2 GDE1 RAPGEF5 CHP1 MX1 FGL1 INSR TLR9 HLA-DMA LOX KIR2DS1 IFNA1 CIB1 HHIP HLA-DRB1 CASP9 PDCD1 BCCIP RAD51 GGTLC1 ADH1B MIR146B SCAF1 CDKN1A SARDH IL32 KLK10 NFKBIE BRD2 MYMX LPL CAT HLA-B PTBP2 CD300LF CRK TCF7L2 CD8B PKM SLC6A12 HSPA1B ADAM17 JAK1 GCG SETD7 F5 ATP12A FAT1 MIR629 GSTO1 ENPP1 MBP CPS1 ELAVL1 P2RX4 SLC2A2 CCND2 PDZK1 DDX58 AKT1 GALNT8 GPC3 PIK3R1 CDKN2C EBNA1BP2 RNF125 MCM6 EIF4A2 CIRBP MIR181C SART3 HILPDA APOBEC3G CCR5 MIR130B ABCB6 MIR150 FCGR1A FPR2 PDLIM3 ABCB11 RORC MXD1 IFIH1 HADHA CYP2B6 CTAA1 BOC NUAK2 XRCC5 SARNP PER1 MIR425 RBM39 RAB1A POU5F1 CUX1 APOC4-APOC2 HDAC1 PSMD7 ALG10 HCC RAD21 PLB1 EIF2S2 USO1 SOD2-OT1 MASP1 MIF CLDN1 MAP2K1 NFKB1 SIRT1 ZNRD2 ABCC1 ACD GFRA1 FOSB ATRNL1 IFNA6 ST13 MIR27B TIMP2 PCBP2 SLC27A2 UGT1A IL1B REN MIR345 MFAP1 ACTA1 G6PC CLIP1 SYCE1L WRAP53 TOMM40 CPE CXCR6 HLA-DOB IGSF6 SMARCA2 CD200R1 TPPP HPSE MIR217 TLL1 LINC01672 H3P10 PDCD1LG2 MBOAT7 IL12B POMC IL9 DDX39B IL11 SMIM10L2A HMGCS1 UBE3A LY9 MMRN1 NCAN RNF19A TXN VIPR1 IGFBP3 GAK SAMMSON BECN1 ALB ARHGAP24 CPT1A DDR1 LMO1 MED19 HAMP SREBF1 DCC KRT8P3 IL21 NR1I2 IGFBP4 MLH1 TREM1 RAB18 PHB APOA1 HLA-C IFNAR2 TIMP4 MIR188 FOXO3 CD69 CX3CR1 NORAD KHDRBS1 AKR1A1 LTA NCOA7 ITGAE SRSF5 IFNB1 POU2F1 GAPDH PSME3 APC TLR2 FUNDC2 MIR499A PVT1 ERVK-9 GPX1 P2RX7 WNK1 CDKN2A SERPINB3 DHX58 TMSB4X MARCHF1 IFITM1 RELN TPR CEBPB RASGRF2 SERPINA13P GEM GAS6 CCL3 SERPINA3 TM6SF2 HLA-DRB3 GSTM2 ATHS ERN1 ATG16L1 JUN IRF9 MT2A FABP2 NF2 NRG1 CDA TBC1D9 DDX41 EPHB2 ACTG1 STON1-GTF2A1L CALM1 ZGPAT TRIM25 DDX3X SESN2 NUP62 HINT1 HAVCR2 HNRNPL CHCHD2 CDKN3 NME1 MPG ITPRIP MAP3K11 ESR2 CISH TRBV20OR9-2 MIR483 SPINT2 TNFAIP8L2 HSPG2 LPCAT1 TFF3 APOL3 TWIST1 FLNA DDX56 EPO PAX5 APRT MRPS18B AAK1 NOTCH1 CD274 NT5C1A LOC102723407 SFRP1 DAPK1 IL1R1 HGS GORASP1 CYP24A1 KIR2DL5A ITIH4 CASC11 EDEM3 GSTT1 NPC1 ATG14 PIM2 LAPTM4B CD40LG SLC17A5 CES1 HGF VRK1 RASSF1 CYP3A4 STX4 PYCARD APOC1 RNASE4 MIR29A NEK2 KLRD1 ITGAL ARL8B ERC1 IL7 ERVK-21 SOX2 BTG3 CH25H TP63 RBL1 PAK2 SMAD6 COMT IFI44 PDLIM5 RPS6KA3 ROS1 IGF2BP1 LANCL1 PRSS1 MDM4 PPIP5K1 ERCC2 MIR216A KRT23 KLRB1 MIR375 ARTN UBC CD1C CYP2R1 MAGEA4 GNB3 MIR1304 AVP PITPNM1 RAB7A HMGB1 S100A9 CSF2 PMS2 ITPR3 EIF2D MMP9 LBR TOP1 SRY ADIPOQ |

| **Table 4** A total of 3881 known therapeutic targets for psoriasis were collected form the GeneCards database. |
| --- |
| CCND3P1 FSTL1 piR-46002-152 KRTCAP2 CD44 MMP2 CEP250 TRAF3IP2-AS1 MIR4677 CCDC116 TUBD1 ERCC5 ATF6B PSTPIP1 KPNA7 CYP26A1 MEFV XDH EDN1 PDHA1 INPP5E HSPB1 LOC105369780 ENSG00000256433 PMPCA CXCR4 ENSG00000260249 AHDC1 C9orf78 MIR6500 IRAK1 piR-55655-210 RNU6-1280P IGF1 ENSG00000284779 G3BP1 MIR20A CXCL3 ESYT1 RAB1B ADCY7 ENSG00000222701 NTRK1 ENSG00000268093 FGFR1OP2P1 ENSG00000258860 LOC102724748 DAZAP1 PRKRA RPL19 TLN1 MITF ENSG00000272072 NOS2 ZBTB12 ENSG00000234290 MN309431 TRIB3 RGN CCNB1 PADI1 LCE3E UBB PON3 ERG TRIM33 MIR6511A2 PARN CCDC88B ENSG00000269621 SEC1P SOD2 RF00017-2570 PNPT1 FGR TMEM235 NONHSAG017238.2 HLA-DRB4 PDE1C LOC107984500 piR-57133-396 SEMA4D FGFR1 BAK1 NSMCE2 CCT8L2 piR-61945-437 TTC37 HLA-DRB5 BANK1 SPN RF00994-819 GOT1 ENSG00000256967 lnc-TH-1 SFTA2 piR-48259 LOC105376214 SERPINA12 SLC12A8 NR4A2 LOC106128905 MN309188 LOC107987207 CXCL13 ENDOU B3GAT1 HMGN1 FADS1 FRA1H LEP HMGB3P4 UBA1 SLC22A2 SERPINF1 TSC22D1 RPS6KA5 MAPKAPK2 SHC1 CCL22 BLOC1S2 PTPN22 ITGA3 CDC25B PSMD10P1 RUNX3 ENSG00000253256 MIR611 ENSG00000285616 BSN PRKCB lnc-IL10-5 HCST piR-48456 C5AR1 RN7SL51P CYP2C19 LOC105376934 A2M LINC00460 KRT1 ENSG00000233902 ANXA3 BBS12 TLR1 ELANE TEK CD160 LYNX1-SLURP2 NONHSAG046097.2 KPNA2 COL1A1 PDCD5 IGH HES1 lnc-IKZF1-6 piR-48918 LINC01620 RBM17 IBD5 PSORS5 IFTAP KPNA3 CD1A piR-41306-095 FOXP3 BIRC5 IFNL1 CHRNA5 lnc-RTN4IP1-6 RF00017-1272 CES2 ENSG00000270210 LINC02569 RAPGEF6 SELL piR-46391-002 EGOT MIR492 TBXT SIRT6 CCL19 HSALNG0007877 NKD1 SCUBE3 RF00017-239 LOC105377062 TNFRSF9 RIPK4 KLK8 EXOSC2 MYL12A YTHDF1 IL17RC HCG21 GUCY1A1 RNU7-15P MAOA APOE PF4 SNRPGP7 ENSG00000257303 BPIFB2 TNNT3 ALDH2 AP4B1-AS1 AIRE ENSG00000231355 MIR9901 C6orf15 IP6K1 ENSG00000267520 EMC1 C3 ENSG00000235978 MN298114-200 SRSF6 MIR181B2 WASHC5-AS1 HDAC7 QDPR MRAP ENSG00000283072 GJB1 ORM1 GJA10 HSPD1 F2RL1 CDK1 MIR99A SIPA1 MIR4662A HSALNG0041080 PTK2 GPN2 GSDMA lnc-TMEM235-1 SHBG LAMP3 FABP5 TUBB MIR200C PMF1 FNDC1 OTUD3 CBLL1 MYH6 IL1F10 SETDB1 FBXL19-AS1 KIR2DL4 TONSL NFKB2 BCL2 TNFRSF10A HSALNG0132861 lnc-RAD23B-18 ORAI3 SON SBNO2 PLCL2 IL24 BDKRB1 NAALADL1 ID1 SNORD16 THADA MIR4802 TNXB PRORP CARD8 LIMD1 BCAM ADAM19 EMX2 CD6 TF HLA-DRA lnc-TEAD4-5 DNMT3B HSALNG0041356 PWP2 ENSG00000249042 RBFADN LOC112267968 EDNRA SNX6 PDE4C ENSG00000285082 RICTOR EXOC2 RAG1 ENSG00000247853 HLA-J LOC111365141 piR-36393-409 CYP1A1 RAB5B IRF3 ARSH EBPL TACR1 NME1-NME2 RF00017-4938 BDH2 MAPRE1 ZMAT5 SLAMF1 RASIP1 LRRK2 PPBP ZNF687 HTR3A LOC102723944 MIR3934 LIF ENSG00000240996 ENSG00000272221 ZNF395 MAPK10 RF00017-4631 POF1B lnc-CLEC16A-8 EPX RASSF5 TRG-TCC2-6 LINC00330 ATAD3B ITPRID1 IKZF3 SETMAR MIR6513 PRINS EZR lnc-FOXD1-3 MIR3927 ATF1 MN309627 ZSWIM8-AS1 IL5 AGT lnc-IRGM-2 RN7SL809P PTGES BBS1 OPRK1 FGF1 IL6R NTAN1 CHRNB2 NFIL3 ENSG00000244061 CHI3L1 CD53 DPPA5P4 CD300A DNAJA3 TFAP2B piR-30396 DHX16 CIITA SLC10A4 CD99 CRP EPHA4 TTLL11 FAM205BP HSPA8 DIDO1 RARB lnc-CAST-4 CBFA2T2 STK40 WASHC5 LINC00993 DPF2 CBARP SPRY2 MIR6509 PRSS56 XPC GSTP1 LTBR CCR3 APOBEC3B ENSG00000220506 ENSG00000255060 SLC9A3R1 C1orf141 SLC9A3 lnc-CCDC8-6 ENSG00000237773 piR-56133-186 ENSG00000235888 STAT5A ASAH2 SPRR2B AURKA MIR122 PTPN2 CATIP RABEP2 LINC02571 C11orf68 AKR7A3 FUBP1 NCR2 PIGF lnc-NDFIP1-1 AY077737 PTGER1 RGS14 IL18BP RNY4P14 C10orf99 PERP KRT15 SCARNA12 YBX1P5 IL17C TNFSF12 PCA3 FN1 OR2W5 AHR KRT17 DHFR LCA5L SYTL1 ASH1L ACTG1P20 BCAP29 LINC02429 UBE2N PSORS1C3 RIMBP3 NFE2L2 IL23R ENSG00000270055 TAB1 SH2D2A lnc-MICA-8 SPINK5 C5orf47 KLF4 SULT1A2 ANKRD52 MIR5010 LMO4 KLKB1 IFI27 ENSG00000232124 ENTR1 MIR3614 SKAP2 HP KLRC1 TAGAP IL1RL2 SNN BCL2L15 HCK ENSG00000272501 SNORA58B AURKB WNT3A RNU6-919P IL22RA2 IFI16 CSTA ASAP2 USP4 NIPAL4 POLR2LP1 BMP6 TRB NPPA CDSN lnc-CCDC88B-1 lnc-TMEM268-4 LOC102723878 TBX21 TIPIN TNFAIP6 lnc-FCGR3A-4 CARD14 CCL26 RTEL1 LGALS7B CASP5 SNAI1 CD151 ENSG00000267340 MANF ARFRP1 piR-56341-240 ATF4P3 piR-55186-001 PRSS22 SOX9 CRABP1 S100A3 RN7SL352P DEFB4A RF00017-5009 TDRD10 GC MIR424 LOC105370655 lnc-IL15RA-4 IER3 ENSG00000218027 FAU SPRR2F PDGFRB IL17D RNU2-47P ENSG00000227836 CARD9 RDX OMD DNAJC9 CTSB JUND WARS1 PHB2 EHBP1L1 ADCY3 PXN TNF KLKP1 SGF29 GVQW3 IKBKG NGFR IL23A KLHL10 LRRC2 RF00001-253 ENSG00000283265 EGF IL1A CMA1 SLURP2 IL1R1-AS1 FYN piR-58029-003 ERCC1 FST AGO2 ENSG00000238290 ITGB1 ZNF384 SCRN1 LORICRIN PARK7 FAM177A1 TAB2 ENSG00000259308 PRMT8 RNF7 UCN2 SELP lnc-SCNN1A-1 POLR3G DCT RPS19P3 XRCC4 PGLYRP1 RCAN1 IRF7 ENSG00000256851 SERPINB13 ENSG00000240399 DNAJB4 THY1 KIT LRBA TBC1D8 ASCL2 lnc-FLI1-5 KRT8 AURKAIP1 MIR210 TYRP1 RFNG NFKBIZ TNFRSF17 PPOX JAK2 PHC1P1 TM9SF4 TNFRSF1A RNU6-474P CASP1 CD8A ENSG00000260937 PRRT1 NSG2 NONHSAG026080.2 GLI1 SPP1 MIR17HG SDR16C5 TNNT2 ZNRD2-AS1 IGFLR1 piR-50893 ENSG00000258740 FDX1 CASP14 XIAP OTUD7A IL13RA1 RGS8 lnc-RNLS-1 ENSG00000199550 WNT5A lnc-KCNH8-7 ENSG00000254135 IFNAR1 MYL10 RF00017-3551 CSNK2B SLC29A3 PTGS2 OSM B2M NF1 APOBEC3A MIRLET7E UBE2D3 ZYXP1 LOC101927897 KLK4 CD2 TMEM176A IL31 AGER HLA-DRB9 TCF19 ENSG00000262692 CHROMR HOXA5 CTNNB1 MIR221 LINC00548 DND1 MICA HNRNPK IL20RA TLR5 MIR141 ANO10 FCGR3A MUCL3 lnc-KCTD15-4 TPI1P2 DNMT3A ENSG00000271913 FCAR NT5E CD200 MIR6090 SPATA48 PSORS9 CD83 WNK4 ENSG00000229817 IL18 NPY ENSG00000259375 OXA1L CCND1 ENSG00000262020 CTSK NOTCH2 PRKDC CD93 CCL4 PSIP1 ZCCHC10 TRPM2 SPRR4 ZFP90 ATXN2 S100B piR-50308-096 ENSG00000273338 ENSG00000259097 LINC01185 ESR1 TAC1 ZFPL1 SLCO1B1 SST ENSG00000235679 NACA PRF1 ENSG00000234789 OLIG1 NFAT5 LST1 piR-38259 RNU6-344P GPX4 MIR4516 CNFN OLIG3 AB372574 SPSB2 CCDC85B WIF1 piR-60146-129 GCH1 PRMT1 CMKLR1 ENSG00000267480 TYMS FLI1 RF00001-209 ZNF750 MCM5 TMEM106C SIK2 ENSG00000288587 RPS29P21 IL15RA APTX RF00017-090 CUL3 LINC01149 BPIFB6 AQP12A ITK ENSG00000224934 lnc-IRF5-3 MSTO2P S100A7P1 SLC48A1 LRIG1 PRSS27 KLRC4 HRAS STK19B KRTAP5-6 ENSG00000220412 PRKACB METTL16 THEM4 DSC1 NDUFAF1 KLK12 piR-38257-014 PDE3A HCG27 L13705-004 PHF12 HSALNG0088853 ZNF354A ENSG00000272477 ENSG00000283413 TMC6 ITGB2 SF3B2 KAT5 CDKN2B BCL6 MAP3K6 IL36G HSALNG0001696 ERH OSTN PROS1 LOC100420500 ENSG00000252433 PSMB8 SLC19A1 VEGFA XRCC1 ENSG00000270120 BSN-DT TMPRSS11D ENPP2 DMBX1 ACAN AREG ENSG00000231128 TGFB1 ITGA2 NONHSAG045737.2 MME APEX1 ENSG00000272540 ACKR2 MRPL11 TUFM LCE1C MIR5187 SNHG32 SPATA2 PGLYRP4 UBE2L3 MYC NTS NONOP2 LINC00892 RNU6-144P SLC37A1 BIRC2 TNFRSF8 PTGIR TMEM259 MUS81 SOCS3 ENSG00000277191 LINC01993 ZC3H12C KIAA1841 MEP1A GPR18 IL10RB DAG1 MACIR TIMP1 NELL2 RSPH3 lnc-REL-2 ENSG00000275015 OR6C4 WNT10B MIR6504 GATD3A PI3 EBAG9P1 MRC1 MAVS MAGI1 VCP GPR160 PWWP2A MG828730-001 THEMIS2 LOC105371083 LINC02539 KRT78 RAVER1 IL13 lnc-TSPAN32-4 RCC1 CD86 KLK9 SLC11A1 LFNG ICAM2 MIR21 ENSG00000256546 L13714-306 FAS-AS1 CTSF GSK3B MBD2 KIR2DL1 ENSG00000229425 TNFAIP3 ENSG00000263033 TSPAN14 MN297907-029 FOXO1 GPT lnc-CEP76-3 MSTO1 CLIC1 DUSP10 lnc-LYRM9-3 PPP1R17 FLJ31104 lnc-IQCH-5 NUFIP1 ENSG00000285837 LINC00824 ADCYAP1R1 MN308699 MMP1 lnc-ZCCHC24-7 HSALNG0130695 STAT1 HLA-DQA1 PTTG1 ENSG00000258790 IGFBP5 ZBTB46-AS1 lnc-DNLZ-1 CTDP1 TARBP1 MMP12 IL18RAP TNFRSF25 CARD16 TNFSF13 lnc-ARFRP1-1 UBE2T ENSG00000212144 piR-41041 RPLP0P3 SLC2A1 MIR326 CREM PTGER4 CCR7 RF00017-356 TRIM47 WEE1 CD109 BCL2A1 CXCL17 ENSG00000237371 BANF1 CR1 RPS6KA2 PRDM1 ADAD1 EBP KRT18P56 TOR1A BSG piR-32214-582 EFR3B TRPV1 KLHDC1 UCP2 TDP1 LOC106780804 RNU6-925P URAD piR-39807-004 lnc-PTCD2-5 ENSG00000236935 ZFYVE9 MIRLET7A1 SLCO1C1 SLC22A5 LOC100129776 KIAA1109 GNLY PTPRC CCR4 ENSG00000285868 MMP11 KIR2DS5 ANKRD65 CD14 HRNR VNN3 HMGCR PPARD CHCHD4P2 XCL1 ANXA2 SMG1P5 CDH3 HORMAD2 PNISR H2BC4 LINC00604 ENSG00000268069 ENSG00000235886 CFAP70 MIR548AY ENSG00000224163 C4B RPS21P8 NCOA5 EMCN DUSP12 MUC22 GSR INPPL1 lnc-RUNX3-2 IL19 ATP6V1F IFFO1 GGH GGT1 DCTN4 piR-43105-135 HNRNPCP4 EIF2AK2 CCL11 DBP AP1S3 UCKL1 SIKE1 C10orf55 WDTC1 IDO1 COL4A2 Hsa-Mir-544_3p-001 MIR6508 C1S CFAP47 RBM4B HMOX1 CD1B FCGR2A lnc-IKZF1-5 MDC1 EGFL8 MC4R PSORS6 CALML5 KRT9 FOXP2 INS FLT1 MIR518A1 ERAP2 SERPINB8 HTR1A PKIG ALOX12 IP6K2 ASH1L-AS1 piR-36455 MICD RB1CC1 EIF4E ABHD5 EBI3 SEC16A SMAD2 LOC105377061 TMBIM1 HSALNG0079512 CCS ADGRE5 piR-48325-110 RNU6-991P LRRC32 MMP3 BGLAP PRKCZ CRHR1 HSALNG0132862 IL20 TRAF6 OR1D5 CCL18 CCR6 CKMT1B TSSC4 CRABP2 CHRNA6 MYL12B F13A1 lnc-WASF3-4 DUSP28 GATA3 ENSG00000267737 RF00017-2389 IL17RD ZNF365 KRT72 GIPC2 PPP3CB lnc-TUBB-17 TUBB2B HHEX CXCR1 LGALS7 USP15 HCG22 FNDC9 NAMPT PTGER2 KRT8P26 ERCC4 LOC105376707 MMP8 ELOA ENSG00000261644 PPP5C IFT172 ERVK-6 CTNS RNASEH2C SPPL2A MIR222 IRAK2 piR-38005-050 LOC105378415 BLMH piR-41318-002 RN7SL277P ADCYAP1 ENSG00000254755 AIMP1P2 GSTM1 MIER2 CRH IL10 ACP5 CYP2S1 lnc-NDST2-5 THEM5 MAPK1 DXO PGF NFKBIL1 CHCHD2P3 ENSG00000258559 SAG UQCR10 RNF126 HNRNPA2B1 CLIC4 MIR214 HIC1 TSC1 IAPP KLK7 ALOX5AP POSTN NKX2-3 ANKH HLA-X lnc-PTGER4-8 BTF3L4P3 CYP27B1 FTO piR-47589 OCLN PPP3R1 ENSG00000248544 ENSG00000272489 NAGLU KRT4 SPI1 ENSG00000286119 IGF2-AS PADI4 RBM14 PLAT RNU4-28P SF3A1 PCAT2 MDM2 SDF4 MIR6503 HSALNG0073924 ENSG00000283782 ATG2A TNFSF11 TAP2 ENSG00000280010 ENSG00000234262 GALR3 CXCL2 GNAI1 EGFR IKBKB piR-44610-008 ENSG00000270640 IZUMO1 hsa-miR-5095-436 TYK2 MYL3 lnc-OLIG3-4 CARD10 lnc-DDX58-3 EEF1D MYLK LOC102724404 LOC105376213 SOD1 PAPOLG MIR130A ENSG00000259202 CXCR3 ZFP36L1 CYSLTR1 GPBAR1 SNX32 MIRLET7B CTLA4 UBAC2 FZD5 AQP3 lnc-CEP76-2 KRT6C PURA ENSG00000262903 RNU6-543P LYVE1 CREB1 ERBB2 lnc-FNBP1-2 UBE2Q1-AS1 RELCH LOC100996583 TLR7 PLCD1 RNASE7 FLG2 STAB1 RPL21P125 LGALS3 LINC02202 MXRA7 MYT1L PDLIM4 SAPCD1 lnc-TMEM17-10 SLC25A38P1 CLEC7A KCNA3 ENSG00000224478 LPIN2 ABCF1 P3H2 IL17F ANKHD1 KIR2DP1 ENSG00000286368 CFL1 IL4 SELPLG CD28 RBP2 RF00017-4000 RPS6KA4 ENSG00000283286 NAT2 PRG2 CNTN1 MASP2 GPATCH3 lnc-MST1-1 CRACD RPS12P3 GBP1 PSMG1 TENM3 MINPP1 ENSG00000249738 CCL3L1 piR-48950-118 EYA3 TNP2 ENSG00000229990 HLA-DPB1 STAT5B KIR2DS2 SLAIN2 lnc-RPL21-6 ENSG00000234062 OAS2 QTRT1 C1RL-AS1 CCNT1 TMEM50B CD74 lnc-ZGLP1-1 PHACTR2 TBL1XR1 KRT16 SP140 MGST2 MG828730-062 TNFSF8 lnc-TMEM50B-3 MAPK8 SLC66A1 DNAJC8 TOM1L1 SRP54 CPEB4 GPR35 CHRNB3 COMP STAR HLA-DQA2 CKMT1A RTEL1-TNFRSF6B RPL3 THBS1 KLB ADORA3 ABL1 SNAPC5 MIR143 LINC02213 PRDM10 TXNRD1 MIR4423 DNAJC27 GZMB RF00017-5401 FCGR3B VIP LACC1 LOC110366354 PDE7A STIP1 piR-48877 FUT11 ELMO1 HLA-G PPP1CB SART1 HSP90AB1 CCT3 NONHSAG031883.2-001 IKZF4 RAD50 piR-50346 HSPE1 MAPK13 RSBN1 MBTPS2 S100A2 KHDC4 C2 NCL EIF4EBP1 GCKR NTF3 SYT8 DPP9 NCOA1 TRPC6 LINC00243 GSPT1 lnc-PSORS1C2-1 LINC01758 LINC00598 DEFB4B GUSB GSDMB EXTL3 lnc-TMEM173-2 LIG1 HRH1 MIR320B1 IL25 HCG18 PLA2G7 GDF11 CCL1 MCM2 CD247 FUCA1 MIR106A KRT18 SOCS1 MIR155 IL17RA SYF2 SPTBN2 BHLHE40 lnc-SUMF1-12 CEP192 LOC100288123 PTH TNFRSF14 RF00017-5196 lnc-NBN-9 piR-52079-016 CCDC184 RB1 FAM76A NONHSAG034319.2 ENSG00000235434 B3GALT6 CETP SLC9A4 PTPN6 ADAR JMJD1C-AS1 PLXNB2 SLC35D1 IFNLR1 TNIP1 TENT5B TM9SF2 SAA1 PF4V1 SRC KNG1 SBK1 CASR ITGAX MIR30E RNU6-70P CDK2 TFAP2A CRMP1 IRF2 SLAMF7 CDC37 PDE4B DDX10 ENSG00000251136 NONHSAG046094.2 TMED10 piR-56902 FGF10 IL1RN KRT7 ODC1 ENSG00000285551 YIF1A LOC107985926 RPSAP49 piR-37170-040 MSMO1 LOC107986649 NRAD1 ZFYVE16 lnc-LARS2-1 CDK11B piR-43099-059 JAZF1-AS1 lnc-REV3L-3 LUC7L3 piR-43939-002 THRA RF00003-002 lnc-ANXA6-3 MAPK11 OAZ3 PHTF1 CA3 ENSG00000285040 ENSG00000184441 MIR22 CD209 PRO2268 RIC8B GJC2 KRT19 TNNI1 SBDS ENO1 LCE3A PCBD1 TNFRSF18 SGSH CXCL10 GALT TPTE SP3 STK32B C20orf203 LOC106128906 lnc-CCHCR1-2 PLG TYR L13712-018 IL10RA IL36RN LOC102725180 STK19 PTTG2 LURAP1L-AS1 lnc-SCGB2B2-133 SLC39A8 MKI67 ENSG00000272630 TSLP ENSG00000255899 ICAM3 IL4R lnc-ZC3H12C-5 CCL5 BTNL2 TGIF1 TP53 C1QTNF12 CCDC69 MAP1LC3A piR-52079-091 NUDT13 MNT ATP6V1G2 ENSG00000255038 PPP6C ENSG00000225421 C1orf53 LOC105378414 OTUB1 piR-55783 ENTPD1 lnc-AHDC1-1 ENSG00000212228 JUP STK11 CHP1 ENSG00000199332 LY6G5B MX1 HORMAD2-AS1 LOXL4 CCR12P SETD1A PLA2G4D LOX ENSG00000254810 SNORD117 MIR138-1 MIR184 LOC105378614 RELB NLRP1 KIR2DS1 KIF3A MN297217 lnc-TMED10-2 ACP1 MYL9 ENSG00000258820 CCN1 ENSG00000274038 LURAP1L SIGLEC5 CASP9 MAP3K7 GRIN3B PDCD1 LINC00184 SNORA31 MIR876 ENSG00000270335 ENSG00000270820 CARS1 ENSG00000261025 CDKN1A BRD2 IL32 SLC2A4RG ADAMTSL5 CAT RPS6KA1 ENSG00000284633 SLC7A1 lnc-TMED10-4 SH2D4B PRKCQ RF00017-3598 RF00017-1621 LAMC2 NONHSAG045920.2 CTRL ADAM17 CHRNA3 LYZ RARRES1 H2AC12 piR-53177-010 RF00017-4713 ZC3H4 OLFML3 LIPJ CPS1 PIAS4 MN309174-057 piR-37824 LRG1 CFLAR USP8P1 GRP DOK5 RNU7-57P USP12 ENSG00000232499 LOC100419513 STAMBP LSM2 FAM205A ANKRD30A MIR6511A1 MIR150 lnc-PLEKHG6-4 IL17B FCGR1A CD47 RPP25L PRKACA ANKRD33B SPRY4 BA000025-001 RORC AB933314 IFIH1 KCNH7 TUBB4B piR-53431-298 XRCC5 AHSG ENSG00000271547 POU5F1 FABP5P12 SELENON ENSG00000263766 KLC2 HDAC1 SMARCAD1 STX1B BMI1 RN7SKP113 PROX2 lnc-HLA-C-2 SLURP1 CLDN1 ENSG00000259276 ICOS NFKB1 piR-36475-004 SIRT1 MANBA TNFRSF6B ZNRD2 ABCC1 SHLD2 KIR3DL3 H3-3B PRM2 SPRR2G IL1B HAX1 NEK9 ACTA1 OVOL1 USP50 S100A7 DDR1-DT CXCR6 piR-47607 LINC01250 ENSG00000288436 CD200R1 HPSE ADNP UBE2V1 ATP8B3 BTD PDCD1LG2 LCAT IL12B ST6GAL1 FCER1G POMC ZMPSTE24 piR-48820-008 IL9 LOC107880064 DDX39B RPL31P43 RNU6-320P RAB10 LOC112268088 RPS25 MIR3617 PRXL2B ST3GAL5 EIF1AD MRPL23 RNF114 MIR381 SFMBT1 RSPO3 HG983680 NFKBIA ANO3 lnc-NBN-5 IGFBP3 lnc-PSMA6-6 GOLGA8R KLF13 DEK ENSG00000205537 PSMA6 CD101 ALB CPT1A PFKFB4 CEACAM1 DCN DNTT NOD2 ENSG00000249624 ENSG00000237669 WASF5P IL21 ENSG00000247121 HTR2A FUT7 FOXF2 EPAS1 MYL6B MLH1 RPL7P32 S100A12 TREM1 ENSG00000255966 CD300LD DEXI piR-55829-003 CKMT2 PHB IFNAR2 HLA-C SPRED2 ENSG00000224228 NRBF2 P2RY11 CX3CR1 ENSG00000260773 ANXA7 ITGAE PCNX3 JDP2 RF00017-4942 GAPDH ERRFI1 MICA-AS1 TLR2 MIR6727 COMMD9 FUT2 lnc-ITGB1BP1-3 GPX1 RSL1D1 P2RX7 CRLF2 SERPINB3 ENSG00000271855 GNA12 piR-52079-043 DHX58 LIMK1 CASP4 ENSG00000215302 POLR2E B4GALT5 ENSG00000202533 TTLL5 IL18R1 MMP19 VNN2 RNU1-7P BCL10 piR-59241 SERPINA3 SLC45A1 GAS6 ENSG00000260302 HLA-DRB3 SNORA73B NAT1 REG3A FABP2 BACH2 KRT79 MMP10 lnc-PPP2R3C-4 TRG NALT1 lnc-PMEL-4 piR-58538-001 EIPR1 ACTG1 IVL POMGNT2 piR-32810-001 WNT7B HAVCR2 HNRNPU CYFIP2 PLAUR lnc-FOXC1-7 ATXN2L FLT4 ENSG00000275103 NME1 S100A7P2 ENSG00000279625 SLC4A4 MAP3K11 ESR2 OLIG2 ZDHHC20P2 MIR483 ADAMTSL1 RPS16 TMEM215 ZBTB46 RF00017-4590 BABAM2 EPO MEP1B PRR9 RPL14 JRKL PGLYRP3 HNRNPA1 PBX2 AKT3 ENSG00000237429 CRB1 LOC107984384 KLK3 IL1R1 LTBP3 POLA2 piR-38051-254 RF00017-4627 EVPL KIR2DL5A ITIH4 PROCR lnc-UBAC2-4 PHF11 PASK IFI27L2 TCOF1 PCNPP1 GJA5 USP8 lnc-ZCCHC24-8 SLC17A5 HGF PIPOX ENSG00000230521 TYMP lnc-ASIC2-2 SYNGR1 PYCARD MIR29A ARID1A ITGAL ARL8B PDE4A CAPZB SATB1 ARFGAP1 THEMIS TMEM176B KRT18P39 FLVCR2 ZBTB40 CH25H ENSG00000272980 ENSG00000229172 CYP1B1 ALPL SMAD6 STEAP2-AS1 COMT CACNA1G piR-50437-360 KCP SPHK2 ENSG00000199490 MIR4686 HMGN2P18 lnc-ACTL7B-11 TRADD VEGFB MIR6514 piR-38512-004 ACKR3 RNPEPL1 KLRB1 TDGF1P3 piR-50444-308 B3GNT2 LTA4H SAFB FNBP1 PNP LINC01932 CD1C CHRNA7 NONHSAG001750.2 ACTR2 DUSP8 HMGB1 HOPX ENSG00000260063 LOC112267902 MED6P1 lnc-UBLCP1-9 LBR lnc-KCNJ1-3 IGFL1 OR10AD1 CCL13 WLS OAT HLA-U CDC25A MLXIP DPH1 MIR5089 RF00017-4966 KARS1 PTGER3 DBNL ENSG00000263050 DONSON RPTOR ADAM30 ERBB4 TSPAN32 lnc-DNAJB4-1 WAKMAR2 MIR3936 HSALNG0133311 piR-57133-201 lnc-FAP-3 FIBP HECA IL12RB1 ENSG00000248648 lnc-IZUMO1-2 lnc-SEH1L-5 CHST8 DIO3 RF00017-3626 lnc-CYTL1-3 RF00019-027 PPIAP9 MIR31 IL26 NBN DNAJB6P4 NPM1P39 COX5A MYL4 AIFM1 GSTA1 MIR146A TGFB2 PLAU TRAF3IP2 RAC1 LAMA2 ENSG00000224431 ENSG00000226032 PTEN KLK6 ANXA1 C1R ENSG00000255086 CD300C MALT1 lnc-FAM109A-1 DELEC1 KANSL2 RAB42 PNKP SETD2 POLR1H KIR2DL2 SENP1 S100P TICAM1 MMP13 HNRNPA1P16 SERPINB5 GPR107 MIR33A TIA1 MN309432 SLC6A4 SPTLC1 SMARCC2 NLRC4 PADI3 HCP5 POLI PKP1 C17orf67 UCN PTRHD1 SYNE2 SLC22A4 ETS1-AS1 ENSG00000225931 NRM lnc-FOSL2-2 CD4 ITLN1 DSP LINC01147 lnc-CLEC16A-4 IRF1 TPD52L2 FABP7 DPYSL2 PTK2B AGAP5 EXOC5 ENSG00000219159 KCTD15 IRF2BP2 CD1D PLAAT4 MBL2 NGF IFI27L1 MSN SH2D1A NONHSAG044958.2 USP20 RF00017-4589 ALOX5 NRP1 CDH13 NLRP3 GJA1 PKD1 LOC106780803 LINC00583 FASN PRDX6 KIR2DS3 AMBP lnc-POU5F1-9 AKR1B10 IL21-AS1 RARS1 ENSG00000204929 SMIM3 piR-52294-068 MIR548AZ TGM2 FCGR2B RXRA CLDN4 DEFB1 H4C8 CSN3 lnc-ATP6V1G2-DDX39B-3 SCARNA10 PKD2 HLA-H PRL LIN54 TIMP3 ARHGAP30 piR-32810-138 RF00026-1103 ACTL7B ENSG00000271581 MIR4742 NR1H3 MPPE1 RPL23AP12 FUBP3 MAML2 ATOH1 TCHH CLCN6 SQSTM1 COG6 CCL4L2 CCR9 NFATC1 CD55 ENSG00000276949 ENSG00000277324 PROK2 SCYL1 SNRNP70 MTHFR LNPEP SMAD3 SEPTIN1 PHKA2 ATF3 CARD11 ENSG00000255726 UCHL1 HMOX2 ENSG00000259407 DDX27 ASB8 PLK1 PLCG1 ENSG00000243886 piR-58297-114 MIR9902-2 DAP3 ZC3H7A PUSL1 SYT2 CARD18 ATP8B2 LINC01220 piR-57133-098 KRT13 PRKAR2A TAPBPL lnc-TNFRSF1A-1 MIR125B2 DPP4 MIR9899 TRAJ23 ZNF148 PAFAH1B1 MIR19A IKZF1 piR-31937-161 lnc-IL6ST-2 HIF3A CNN2P9 H2AC18 ENSG00000212553 ENSG00000227540 FBLIM1 KCNA2 IFNA2 PLTP PLA2G2A TBC1D5 BRMS1 LCE1B MIR6507 HRH4 ENSG00000230928 SPRR2D LGALS1 UBQLN4 ALK TNFRSF14-AS1 EZH2 ENSG00000230684 RAP1GAP2 DOCK8 GAN ENSG00000255389 VAMP1 HLA-E HLA-S FABP5P2 ENSG00000238280 SLC25A5 RAB37 GON4L FAS ENSG00000266728 LILRB4 PON1 ARNT MRGBP ENSG00000254754 HBEGF BDNF PI4KA ENSG00000258317 LINC01273 OSMR PNMT RPS5P3 SEMA3A GNPDA1 FOXC1 LSM14A USP49 CYSLTR2 RPL12 IQCH MIR223 FGG RBM4 CSF1 lnc-MUC22-1 TNFSF10 THBS3 TRNAU1AP NABP2 SMARCE1P5 COPA CKB NUTM2D MUC1 KIF11 SLC26A3 HLA-W LOC105371887 PTHLH WDR43 MYO5B TRMT112 TNFRSF11B MTHFSD CADM2 CD36 UBLCP1 TENT4B POLM MYH9 MIR10A CLK2 ZPBP2 TTYH3 ENSG00000254926 piR-48852 MIR215 TNFSF15 RF00017-1735 lnc-NFKBIA-4 NCF2 BDKRB2 SEC31B S100A5 XRCC6 lnc-RIPK2-3 HUS1B ENSG00000272109 NONHSAG045731.2 CTSW NOP2 NNAT ENSG00000255320 ADAMTS9 MRPL28 lnc-TNFAIP3-10 STMN3 PIGV TRAF3 CXCR2 PCP4L1 COMMD7 RPS4X IFNG-AS1 ARHGAP45 CCHCR1 lnc-GTF2H4-5 RPS3 HQ292134 lnc-CAB39L-9 STAT4 CICP4 TNNI3 MIR181B1 CD40 RNF145 NONHSAG046439.2 lnc-ZMAT5-2 CTSV MIR6510 lnc-FOXC1-2 LOC285626 GPATCH2L ENSG00000276527 PDXK ENSG00000283360 ENSG00000287967 CASP8 APOB RAB11FIP2 PIGR CFH TAP1 HCG17 NRIP1 TCHHL1 CS ETS2 ENSG00000205622 IGFBP7 NRP2 FCRL3 PRDX1 FGB ITGA1 MIR647 MIR203A ERMP1 LAG3 FLNC TSGA10 lnc-CCT4-2 ERAP1 PLCH2 RF00017-349 ADGRL2 EMG1 CAVIN1 C4A NCEH1 PPARG GRIN2C ORMDL3 ENSG00000248601 piR-31162-048 ST2 ALOX15 CHGA CACNA1B HSPA4 PAN2 SERPINB7 CYP4F8 NEAT1 RF00618-001 NUPR1 EMC3 ENSG00000207300 DCLRE1B ZWILCH CCL7 PRKCD CALCA piR-43105-342 DSC2 LPXN RBMS2 CXCL11 PSMG2 TRIM65 SERPINA1 DTX1 ANTXR2 COL4A3 IL31RA LOC105373724 C11orf58 LINC02693 PPIG piR-39098-183 NPPA-AS1 MYL6 TAPBP RPSA RUNX1 FADS2 IL1RL1 KIR3DL2 RF00998-085 HLA-DQB1 MAMSTR MTTP CENPO CXCL8 MAPK9 RPS21P1 ZNF366 EGR1 CCL17 RIPK2 CLDN7 CHRNB4 CD58 GLP1R IL36B LITAF GRHL2 IFNL3 lnc-SDF4-1 LRRC43 SELE BPIFC lnc-ARHGAP20-52 IL17RE ABCB1 KLRC2 CNR1 GPR12 MIR9903 piR-43105-140 piR-41306-103 piR-55654-049 HECTD4 CTF1 ENSG00000287597 PSORS7 RF00066-120 TGM5 NR3C1 SCUBE1 BORCS5 MTND5P15 CCL27 DCD POLA1 IL17A RPL21P49 PNLIP ENSG00000261216 LEPR TRL-CAG1-6 MTOR NONHSAG041785.2 MCM3 MIR124-1 ENSG00000236304 RPS2 PPBPP1 NRBP1 LOC107985465 INS-IGF2 WT1 CARD6 lnc-RUNX3-3 STEAP4 SLC22A1 IL13RA2 ZNF816-ZNF321P MAPK14 SERPINB2 TNFRSF4 PITHD1 AOC3 FABP3 GJA4 DNAJC9-AS1 CCNL2 KSR1 lnc-SRP68-1 RF00017-6604 ENSG00000274737 MYPN MIIP ENSG00000285552 CDK6 EIF2S2P3 ANXA6 CDADC1 LRIG3 lnc-ARHGAP20-11 CSK TFPT DPM3 EIF2S3 piR-55650-032 KLLN CTSS SMPD1 TH lnc-USP20-5 ENSG00000285647 lnc-IGF2-4 ZC3H12A LCK LOC105377139 CNTF ACKR4 AFF4 MAP3K8 QPCTL RAF1 SMIM33 STC1 lnc-GSDMC-13 RPAP3 PRPF6 TRD lnc-TMEM258-1 KLRK1 GSN lnc-SLC35D1-9 ENSG00000281883 EGR2 LTB4R2 NCR3 ENSG00000224988 ENSG00000229162 CPSF3 RSRP1 RPL21P33 CD248 CCR2 SULT1A1 CDC37P1 YY1AP1 PLA2G4A DNLZ AKR7A2 KRT76 CEACAM4 SMAD7 FAM99B MICB-DT BOLA2 VEGFC lnc-DDR1-4 LOC101929574 SDC4 MPO ZBTB16 PDGFB ENSG00000249743 ELN KLK2 VIM ENSG00000271936 LINC01430 ADRA1B LTB TLR8 S100A14 MIR6515 ITGAV FGFR2 HLA-DMB HCG26 BRD7P4 ITPKA PIP5K1A CSF3 PCNA ACRBP AMY2A REV3L PRKAR1A ENSG00000242162 HLA-A ATG5 RNU6-299P ADAMTS4 TNNI2 ZGLP1 MIR518B MIR99B CD7 EDNRB CYC1 CD27 SLIT3 PEX13 piR-59316-009 CD207 BAGE2 YDJC CMC1 piR-49770 DYDC2 HSPA6 CHUK RAB6B CHAT NLRP7 NR1H2 piR-51449 S1PR4 lnc-ITGB1BP1-2 CD226 CPT1B KYNU STAT3 ENSG00000227938 CCL20 SPHK1 TRIM26 AC003959 IL22 MYH10 RPS26 IL34 MAD1L1 TRPT1 ENSG00000263307 ENSG00000245156 ZSWIM8 IFNGR2 MCCD1 piR-40110-155 ITPR1 NPAS4 CAP1 ARG1 RF00017-2427 LOC105447645 HLA-K ASCC2 DUSP1 OR10P1 ENSG00000248734 SPRED1 CYTH2 SLPI OLAH ADAM10 TRIM27 GALC ADO LUZP4 STING1 H19 TBKBP1 piR-35674-441 TSPAN33 TGFBR1 CCR5AS TGM3 RGCC piR-45012-401 lnc-KLLN-4 MCHR1 VTN BAD EBF1 ENSG00000255135 CD300LG ENSG00000285413 ACO1 SPINK7 SEMA7A IL15 ATM MN309221 ATP5F1A HSALNG0045893 GTF2H4 ENSG00000235620 HSPA1A LIG4 PFKFB3 MIR9900 EREG LINC02354 IL17RB SFRP4 MCAM AGTR1 IPMK MC1R RNF168 CAMP MTERF4 GRHL3 AHSA2P LINC02863 USP2 NONHSAG008489.2 MYL1 CISD2 GBA TNFSF18 MIR4520-1 SCARNA11 RF00017-7990 SFN RF00017-5679 CLMN ESD TNS1 lnc-CNTF-2 IL1RAP IL6R-AS1 HSALNG0088095 MTMR10 PRDX5 PYGO2 CSNK1D CCL4L1 PPP1R10 CASP7 KIR3DL1 TJP1 TRIB1 MIR623 PRM3 PTX3 ICOSLG DSG1 SLC26A11 CCK piR-32287-039 FAM118A NDUFB6 LYPLA2 DOK6 BA000025-002 U2AF1 SMAD4 CD3D ENSG00000231344 CSMD1 CTSD MIR6506 RRP1B NHEJ1 PFKM STAT6 MIR1208 GNL1 DNAJC18 GART GID8 IL3 RF00017-5697 RF00017-4377 TTC7A ENSG00000167807 S100A6 ERCC3 TP53BP1 PTCH1 ENSG00000229836 S100A1 TFRC LINC02800 VARS2 PTPA MIR6502 SPRR1A SCEL LELP1 RF00017-4972 SNX20 MIR197 ILF3 PNPLA8 RF00017-3134 ENSG00000229299 ENSG00000269930 KEAP1 MST1 MED16 SERPINF2 lnc-GOT1-1 JAZF1 IGFL2 ZBTB39 TYRO3 S100A8 lnc-WASHC5-9 ENSG00000267199 lnc-CREB5-4 RF00017-107 RNF5 HYDIN piR-43107-300 MMEL1 ENSG00000268810 KLHL25 TRAPPC10 ENSG00000226197 PRXL2A ENSG00000251244 MGMT CPQ ENSG00000272791 MYLPF AIF1 SOAT1 RN7SL391P HCN3 GAB2 piR-43325-002 LIX1 LAMA5 F8 ACHE TCAIM TNFRSF12A SKIV2L lnc-PDGFB-3 ATF4 MRPL57P8 OR5B21 IL12A ARNTL LCE3B MCL1 RF00017-299 piR-51327 CEP43 CCL2 PUS10 NPM1P17 FBXL19 TXNIP PRNP OSGIN2 ADCY10 lnc-AKR7A2-3 MIR187 ADA S100A11 SPATA7 LAMC1 CCND3 IL27 CLTC DOT1L NEB KLK15 PSORS10 EIF5AL1 IL6 TLR3 CAMK2G ERVW-1 KCNK4 HSALNG0032571 HMGB2 ANXA5 ENSG00000257449 PTBP1 CCL24 BATF ANGPT1 SNODB1279 CPT2 PTGDR2 PSME1 GMEB2 EEF1A1 PUDPP2 LIMK2 TOR1B EPHA1 RF00017-3288 RPSAP64 TSGA10IP HNRNPC lnc-GCA-5 PSTPIP2 RF00994-809 LTB4R SUGT1P3 ISG15 piR-38580-002 UBE2D1 SF1 CREB5 HSP90AA1 CASP3 piR-48325-111 FRG2C ANGPT2 UBASH3A TSBP1 ZNF831 UBE2G2 CCRL2 lnc-IL12B-2 PSORS4 BAG6 CXCR5 SLC7A2 KRT2 MIR3936HG TH2LCRR ENSG00000286629 FLNC-AS1 HSD3B7 ITGA4 RIN1 piR-61945-308 LOC110806262 VDR ACE ENSG00000286186 RF00017-092 IRF4 PRKCE LOC101059986 INTS11 DEFA3 ITGAM RNU1-134P TUBE1 STX3 CD34 ENSG00000254566 ENSG00000288583 IFNL2 TGM1 PTPRK TPSAB1 piR-45035-151 PDPN MIR9902-1 MST1R PHACTR4 ARPC2 ACYP1 ENSG00000268584 FASLG PPT2 GLIS1 MIR126 TAX1BP1 SNHG3 INAVA HLA-F RXRG RPL35P9 ENSG00000269919 KIR2DS4 RF00017-5412 SGPP2 MIR125B1 CD163 DUSP19 KDR MIR330 DNAJC14 KRT14 FOSL2 DNM3 ABCG2 IL1RAPL2 EEF2 EPHA3 RPL15P4 MUC19 R3HCC1L P4HA2 piR-56451-016 MIR9898 piR-52079-106 MEN1 GPR65 ENSG00000219410 ENSG00000199349 DFFA HSALNG0021688 TMEM158 RARA piR-47234 PAXX CARM1 LINC00302 lnc-TMED10-5 ENSG00000258199 C11orf21 PECAM1 LATS2 MYD88 TNC PTPN1 DOCK9 ITGB1BP1 RF01045-135 BRAP CCR8 PPP1R12B LOC112268240 MIR4425 CD27-AS1 GPATCH1 DNMT3L DAB2 MIR145 DUSP8P5 SERPINH1 STARD6 PPARA CNR2 RACK1 CD244 lnc-TTC33-6 COX6CP1 KRT27 PMEL RORA HSALNG0046732 LSP1 ALOX15B CD59 IL2RA PNRC2 CD24 FAP PLK2 piR-53698 CCR1 CLDN5 PTPRN2 ZNF512 PSAP SMURF1 LYRM9 PRIM1 CYP1A2 PAIP1P1 NUDT9 TNFSF4 TG ENSG00000228778 LYN TGFB3 MAPKAPK5 POMP LINC02723 HGD ZFP91 RBP4 EAPP NME2 RF00483 ADAM33 IFI6 S100A4 LIME1 BTC FRMD8 MAP2K5 lnc-SUMF1-19 NUP188 LGMN MSH5 SLC36A1 GPR183 PPAN-P2RY11 LEPQTL1 RPS20 LAMB1 KCNH8 lnc-IZUMO1-1 MYRF IGF2 LILRB1 BPTF CTSG NPEPPS TXK CD80 B3GNT4 RARRES2 SNX27 NXPE4 ICAM4 MICOS10 FPGS ADPRH ENSG00000254855 DHFRP2 ID4 FGFR3P1 CDH22 NUBP1 CDK5RAP1 MIR486-1 P2RY6 ENSG00000224195 IL12RB2 MCOLN1 RASGRP1 TRIM21 PSG2 NLRC5 GAL3ST2 lnc-ICAM3-1 LOC107882126 KIR2DL3 GZMA FABP1 IL9R RNASE3 ENSG00000286022 PSORS1C1 PIK3CG RF00017-5121 CYTL1 ACKR1 ENSG00000272599 LPA MHENCR FGF7 CFAP126 IL37 IL17REL KLK13 ANKFN1 NAT9 MB NQO1 CBL C20orf181 SP1 piR-43408-221 SEC24C APOBEC1 DENND1B MIR200A SPTLC2 LCE3D NDST2 SKOR1 CD79A PSORS11 IL1R2 LCEP4 PIK3CA NLRP2 FLT3 TNPO3 SERPINB4 IRGM IFNK TSPO CKLF ENSG00000271821 PLEKHN1 MBNL1 LOC102725238 IL2 FOXA1 ERBB3 USP21 VNN1 lnc-TM9SF2-4 piR-56480-015 piR-43104-029 RNU6-147P ATAD3C PLCL1 SDC1 MG828475 C5orf66 SYK ENSG00000259005 FGA SERPINB1 FREM3 AP5B1 RN7SKP226 C11orf87 LOC101927911 CDH1 DOK3 GRIN1 MEIKIN TBR1 FOS IFNG CALML3 ENSG00000257869 HSPA5 RETN FNDC5 JUNB CCL8 TNFSF13B SYS1 CYP27A1 IGES ENSG00000262580 HCG4B TMCO4 ASIC2 ZNF300 LINC02098 MIR135B GNAQ BAZ2A XPA RNA5SP184 ENSG00000287850 MIR142 ENSG00000144785 LOC642361 ENSG00000261338 ENSG00000234261 FGF2 CLU piR-52916-031 LINC02694 IRF5 CXCL12 TMEM198B LPAR5 TFAP2C RF00017-3711 IL36A HNRNPM SRSF1 ING4 PCAT4 COL2A1 C4orf17 SPRR2A FCGR2C EEF1E1 MSX1 FNDC3A LOC101928120 CEP76 CORO1A LINC01989 MICB ENSG00000269514 ELOVL6 MRE11 COG5 CST6 CD63 EPHX1 MYL7 NCR1 CD52 TOP2A CX3CL1 DLX3 UBE2E1 ADAM1A lnc-PQLC2-5 PRTN3 NR5A2 ENSG00000283648 ASB6 FEN1 IGF1R PPIF KLRC4-KLRK1 RIPK1 GJB2 KLK5 PRKD1 KIR3DS1 ENSG00000283504 lnc-FCGR3A-2 NONHSAG042082.2 EFEMP2 ICAM1 CALCRL EFNB2 RECK CXCL9 CAV1 DGAT2 lnc-SUMF1-18 BCL2L1 CEBPG CD19 TNFRSF1B PSORS8 RAPGEF3 LGALS9 MAPK3 NAV1 lnc-RTTN-6 RBM22 ZDHHC23 IGHA1 DBF4B AQP12B CCL28 RF00017-5406 ETF1P1 RNU6-283P PRR5L SLC9A1 DNAJC28 CD300LB PLEKHG6 THBS2 CTSL CA2 lnc-MAP3K7-3 FOXF1 LRRC7 piR-41195-025 VWA7 TPO IL6ST TEX41 NCAPD2 ETS1 EDC4 MRPS27 ENSG00000279145 UBAP2L IL22RA1 POLR1D TEC CXCL1 MIR6499 MIR100 ENSG00000275915 PC TEX48 PAK5 GPN1 ENSG00000271553 EIF3C MIR1301 MIR6501 HNF4A CD81 RCE1 TNFRSF10B SH2B3 ENSG00000273112 CDK5R1 CTDSP1 TRIM39 CHD4 S100A7L2 lnc-KCTD15-5 TMEM116 ENSG00000206734 CRNN IKBKE piR-55948-040 HIF1A RELA GRN IRF1-AS1 TRERNA1 RF00017-5011 piR-59425-015 MIR133B TRIM32 PKMYT1 RPS19 DUT PRKACG KHSRP EDEM1 GLYAT NOS3 DDC TNFRSF11A TGFA FLJ31356 MMP17 ANPEP MIR194-1 MYL5 PRLR ANGPTL6 ATXN2-AS LINC01475 ENSG00000261367 MIR6511A3 RPS23P10 PADI2 MN298114-130 DST NFATC2 lnc-SLC37A1-2 LOC400867 lnc-CTNND2-10 FLAD1 RAB5C ENSG00000231557 CD48 lnc-GTF2H4-6 RNA5SP192 lnc-GPX3-2 MSH2 lnc-APOBR-1 FAM27E5 ST14 ENSG00000205414 RIT1 CYLD HIF1AN ENSG00000287771 GDI2 LINC01208 FGD5 XBP1 WTAP CEBPA FDX2 piR-38580-144 piR-49732-033 NFKBIB JMJD1C USF1 ALOX12B ANKRD33B-AS1 lnc-PWP2-2 PRRC2A AAGAB OLFML2B S100A10 ENSG00000253111 TDGF1 PA2G4 PRDX2 APOBEC3C RUSC2 ATOD3 BAZ1A TRIM10 CADM1 lnc-SUMF1-4 NOTCH4 INPP5D TMEM17 CD5 CD3G TSPAN9 NONHSAG031883.2-002 COL17A1 OPRM1 C1orf189 CLYBL CALM3 C6orf136 IL7R ENSG00000252920 IFI35 LYNX1 TLR4 XKR8 RF00017-2046 RAB12 CFHR2 MPDZ CCL21 ATF6 RPTN IL16 MIR6505 VCL LINC02708 TCF3 DNASE1L2 SERPINE1 HFE CCR10 ENSG00000238326 TXNDC11 IGFL3 POU2F3 HLA-L RARG ATAT1 MIR17 DENND1C VCAM1 PSMD1 piR-61101-149 STAT2 MLH3 PTAFR CD1E REL ENSG00000237493 RNF186 MIR203B EWSR1 ENSG00000287682 LOC105371082 ENSG00000286116 INKA1 ENSG00000259762 CPTP lnc-EMC8-1 RF00017-6555 DYDC1 RBMX SMG6 CRTC3 KRTDAP PSORS1C2 TGFBR2 TLR9 TTN IL33 RGS6 HLA-DMA lnc-STMN3-5 GPR137 INAFM1 AKAP13 FLG lnc-SNAI1-2 IFNA1 MIR194-2 LINC02085 NFX1 NONHSAG032712.2 RF00017-2555 HLA-DRB1 ENSG00000267543 RPL7P1 LOC105376976 CLEC16A LOC105369565 RPL41 ENSG00000285784 piR-32287-040 DRAP1 MIR146B DLD PAICS CDK5 LINC01845 KLK10 PADI6 NOG piR-32285-085 piR-41306-110 ATP5F1B MLANA HLA-B HMMR CD300LF piR-31937-039 TCF7L2 piR-48007 RNASET2 PKM piR-39701-054 JAK3 LINC01714 GBAP1 PSORS3 JAK1 FCER2 GCG SLC39A11 CXCL5 piR-33432-055 RF00017-5045 MIR4673 IL12A-AS1 RF00017-2723 LINGO4 ITGA9 SPRR3 PIP ACSL6 MFSD4B FLOT1 lnc-IRF1-7 SCNN1A ULK2 lnc-PRDX5-1 FABP4 CANX MN309174-660 GPANK1 PPP2R3C ENSG00000272279 ZMIZ1 CCND2 DAP SLC25A28 piR-31432-064 DEFB104A DDX58 CFB AKT1 RNU6-850P CXCL6 RNF125 TMEM258 TRIM39-RPP21 RNU4ATAC4P SART3 ACTA2-AS1 ZC3H10 CAPN10 APOBEC3G ENSG00000230533 CCR5 CISD1 FABP12 POFUT1 MLNR STAC lnc-SCGB2B2-4 VEGFD SEPTIN8 KRTAP5-5 MN298214 lnc-ETS2-5 ERV9-1 LILRA3 ENSG00000267317 KRT10 PHETA1 lnc-ANKRD33B-5 SLC9A8 FZD6 TXNRD3 POLR2C PON2 GPX3 RALGAPA1 YAP1 ENSG00000202268 ENSG00000257740 LOC102723798 MIR4640 KLK1 MALAT1 SIK3 PLB1 PRR12 LOC107984859 CD164L2 MIF RUNX3-AS1 ENSG00000271267 MAP2K1 GH1 C4BPB AIM2 PFN1 MIR6512 piR-34822-272 IRF8 LOC101928372 SNORD74 BCL3 KRT5 lnc-SBNO2-2 TIMP2 piR-50437-058 ENSG00000254461 TOPAZ1 NONHSAG011344.2 RPS9P4 SYVN1 LPCAT3 GLULP4 CCDC22 LTC4S RUSC1-AS1 FCRLA TTPAL MIR217 TLL1 TOMM20P3 OAZ1 AMT DUSP16 RF00017-3411 DEFB103B IL11 RASL11A F11R HSALNG0096771 KITLG ENSG00000267303 FLG-AS1 S100A7A LCN2 SUMO4 AKR1C3 ARID4B NONHSAG002957.2 TXN TMEM199 piR-48325-029 ACTA2 RN7SL608P IL20RB DDR1 IFN1@ PTGS1 SERPINC1 MIR6511A4 MAP4K4 MFNG CTSZ NR1I2 ENSG00000230404 KIF21B IGFBP4 DLAT lnc-ICAM3-2 LMNA SLC9B1 GMNC IGIP lnc-PERP-9 TBCD ENSG00000275693 NXPE1 ENSG00000275850 ENSG00000229288 EIF3FP3 APOA1 SEPHS2 TATDN1 PYGM KPRP KPNB1 lnc-PRR5L-1 CD69 LTA LARS2 HSALNG0025502 MYOZ1 IFNB1 PPP1R18 piR-39341-005 RF00017-6124 piR-33458 HSALNG0043662 PVT1 CDKN2A C18orf54 KLK11 CARMIL2 ENSG00000261560 CD300E RAG2 RELN CEBPB ANKRD55 CASP2 SUPT7L HSALNG0094635 ZNF341 CCL3 RF00017-1397 MN309183 MFSD9 ENSG00000259630 ATG16L1 ERN1 TRA LCE3C JUN CSF1R IRAK4 MYL2 DHODH PTH1R ADAMTS5 NOTCH3 RPS14 RBP1 ATIC CXCL16 HSALNG0049229 HSALNG0084946 lnc-C9orf78-2 ADAM15 NRG1 CAST GAB1 ENSG00000266527 TSBP1-AS1 DDX41 PRSS53 UBE2D2 MTMR3 ZGPAT OVOL1-AS1 RPL3P2 CISH LOC105375746 UBE2Q1 ENSG00000283579 ENSG00000251405 CLCF1 CYP2J2 SCG5 lnc-TNFSF18-3 HSPG2 TFF3 NDFIP1 ENSG00000236710 ENSG00000266202 LINC00581 FLNA DPP10 IL2RB lnc-TNFSF15-5 WHRN NOTCH1 BAX CD274 ECD NONHSAG019426.2 CP IGHE ZNF816 SSTR5 RPL37 CDKAL1 ENSG00000286543 RMI2 SUOX CDH5 ATF4P4 hsa-miR-5096-095 FKBP1A CYP24A1 RFTN2 BPIFB3 TNFRSF10D FOSL1 PRKCA GSTT1 SCARNA5 CD40LG RN7SKP9 SPRR2E ENSG00000197254 SRF HCG20 HIPK1 ADD2 ZNF300P1 ICAM5 C2orf74 CYP3A4 CLN3 SATB1-AS1 STX4 LRIG2 KLRD1 CLIC6 PYDC1 UBBP4 EMSY MIR4524A IL7 BHLHE40-AS1 TTC7B DPYSL3 KAT6B ENSG00000273055 JCHAIN SNRPGP8 S100A13 TP63 PEPD ANXA11 TOB1 VHL ZNF358 ZPBP EEF1G NUSAP1 WASF2 ABHD16A lnc-ANKMY1-3 HNRNPA1P41 C1orf68 FGF23 PRSS1 PTGIS piR-55361-507 lnc-MRPL23-2 PAH ERCC2 CEACAM3 PNKD VARS1 SPRR1B FLRT1 DCLRE1C RNU6-343P UBC PIGCP2 PRR3 ATRN CYP2R1 PSMB9 TINCR HAPLN1 ENSG00000260257 TNNT1 GPR15 S100A9 CSF2 lnc-MRPL38-1 IL11RA RPS6KB1 MMP9 DHRS2 LINC02791 ELOA-AS1 lnc-HLA-C-3 piR-59907-003 CRCT1 CTSC RNU6-586P RXRB ADIPOQ |

| **Table 5** The 389 overlapping genes between the psoriasis and hepatitis C. |
| --- |
| MMP2 CXCR4 IRAK1 MIR20A SOD2 SEMA4D FOXP3 IL24 CYP1A1 GSTP1 MIR122 HP IL17D JUND IL23A CASP1 IFNAR1 MIR221 CDKN2B PSMB8 MME SOCS3 FOXO1 ANXA2 FCGR2A EIF4E TRAF6 IL10 MDM2 TAP2 EGFR MIR130A LGALS3 CD28 MASP2 OAS2 PTH ITGAX IRF2 IL36RN TSLP ZNRD2 IL9 ALB TREM1 HLA-C IL1R1 KIR2DL5A RPTOR MIR146A HCP5 MBL2 AKR1B10 PRL SMAD3 LNPEP MIR19A PON1 MIR223 TNFRSF11B MRPL28 CXCR2 CD40 SERPINA1 KIR3DL2 CCL17 ABCB1 KLRC2 IL17A MAPK14 ANXA6 ZC3H12A TLR8 HLA-A CD226 STAT3 PTX3 S100A1 CPQ SOAT1 IL27 ISG15 CASP3 ITGAM PDPN PECAM1 CCR1 LPA FOS IFNG RETN TNFRSF1B CXCL1 NOS3 HLA-DRB1 ATG16L1 ERN1 IL7 HSPB1 IGF1 CYP2C19 IFNL1 ALDH2 C3 MIR99A IRF3 CRP FN1 CTSB TNF IL1A SPP1 S100B ESR1 SPATA2 TNFRSF8 CD86 STAT1 PTPRC CCR6 MAPK1 CXCR3 MAPK8 HLA-DQA2 IFNLR1 IL1RN MKI67 CCL5 PDCD1 IL32 CAT POU5F1 NFKB1 ABCC1 DDX39B IL21 PHB P2RX7 NME1 COMT CD1C SLC6A4 NLRP3 MTHFR TNFSF10 APOB LAG3 CAVIN1 PPARG PPIG RPSA CXCL8 CTSS SMAD7 CSF3 IL22 SLPI CCL4L1 KIR3DL1 IL12A ADA HSP90AA1 CD34 RARA MYD88 CD244 IL2RA CD24 TRIM21 FABP1 IL37 SERPINB4 TNFSF13B ICAM1 CD19 MAPK3 CA2 OPRM1 CCL21 IL16 SERPINE1 CCR5 MIF CD69 LTA IFNB1 CCL3 CD274 ADIPOQ NOS2 TRIB3 LEP CCL22 PTPN22 RUNX3 ELANE TNFRSF9 APOE SHBG BCL2 AGT IL6R PTPN2 AHR NFE2L2 IL23R IFI27 CRABP1 RNF7 TNFRSF1A MICA IL18 NOTCH2 IL15RA VEGFA MIR21 GSK3B HLA-DQA1 PPARD AP1S3 NFKBIL1 THBS1 MIR155 PTPN6 CXCL10 CDKN1A HPSE POMC CX3CR1 ITGAE SERPINB3 ITGAL HMGB1 IRF1 PLAAT4 FAS STAT4 TAP1 GLP1R CNR1 SLC22A1 CD27 IFNGR2 DUSP1 TRIM27 STAT6 IL6 VDR ACE FASLG MIR126 CD163 KRT14 ABCG2 IFI6 PIK3CG PIK3CA IRGM SDC1 CYP27A1 CLU CXCL12 CX3CL1 KIR3DS1 CAV1 TNFRSF10B CDK5R1 RELA XBP1 TLR4 MIR17 TLR9 HLA-DMA HLA-B TCF7L2 JAK1 DDX58 AKT1 PLB1 NR1I2 APOA1 CDKN2A JUN NOTCH1 GSTT1 CD40LG CYP3A4 S100A9 IGH PF4 CHI3L1 AURKA KLRC1 IL1RL2 BMP6 EGF WNT5A CTNNB1 FCGR3A CCND1 CCL4 TGFB1 TIMP1 MRC1 MAVS IL13 CREM CCR7 CD14 IL19 HMOX1 FLT1 SMAD2 IL20 GSTM1 PGF CYP27B1 TYK2 CTLA4 MIRLET7B TLR7 IL17F IL4 HLA-DPB1 GZMB FCGR3B HLA-G GCKR SOCS1 SAA1 KRT7 TP53 KIR2DS1 ADAM17 IFIH1 SIRT1 IL1B IL12B GAPDH TLR2 GPX1 ESR2 SLC17A5 PLAU PTEN ANXA1 CD1D TGM2 NR1H3 DPP4 LGALS1 HLA-E FOXC1 MUC1 ERAP1 CXCL11 HLA-DQB1 CCL27 MTOR AOC3 CCR2 PCNA ATG5 CCL20 ARG1 IL15 CCL2 ANGPT1 ANGPT2 MIR145 PPARA RBP4 IGF2 ACKR1 IL2 JUNB IRF5 CXCL9 TPO TGFA NOTCH4 STAT2 IFNA1 MIR146B GCG CYP24A1 TP63 CSF2 MMP9 |
