# Supplementary material for: Is there a relationship between psoriasis and hepatitis C? A meta-analysis and bioinformatics investigation
Source: Virol J. 2021 Jul 2;18:135. doi: 10.1186/s12985-021-01606-z (PMC8252322; doi:10.1186/s12985-021-01606-z)

**Additional File 3:** Functional annotation of overlapping genes between the psoriasis and hepatitis C.

| **Table 1** GO enrichment entry | | | | |
| --- | --- | --- | --- | --- |
| Category | Term | Count | Gene Ratio (%) | PValue |
| BP | GO:0006955~immune response | 95 | 24.6114 | 4.27E-69 |
| BP | GO:0006954~inflammatory response | 87 | 22.53886 | 1.38E-63 |
| BP | GO:0071222~cellular response to lipopolysaccharide | 39 | 10.10363 | 4.00E-35 |
| BP | GO:0032496~response to lipopolysaccharide | 42 | 10.88083 | 5.51E-32 |
| BP | GO:0043066~negative regulation of apoptotic process | 56 | 14.50777 | 1.27E-25 |
| BP | GO:0045944~positive regulation of transcription from RNA polymerase II promoter | 81 | 20.98446 | 1.56E-25 |
| BP | GO:0070098~chemokine-mediated signaling pathway | 26 | 6.735751 | 4.90E-24 |
| BP | GO:0042493~response to drug | 45 | 11.65803 | 8.61E-24 |
| BP | GO:0031663~lipopolysaccharide-mediated signaling pathway | 19 | 4.92228 | 2.86E-22 |
| BP | GO:0050729~positive regulation of inflammatory response | 24 | 6.217617 | 6.14E-21 |
| BP | GO:0007165~signal transduction | 80 | 20.72539 | 2.73E-20 |
| BP | GO:0045087~innate immune response | 47 | 12.17617 | 2.34E-19 |
| BP | GO:0042102~positive regulation of T cell proliferation | 21 | 5.440415 | 6.35E-19 |
| BP | GO:0010628~positive regulation of gene expression | 37 | 9.585492 | 7.12E-19 |
| BP | GO:0001666~response to hypoxia | 31 | 8.031088 | 7.13E-19 |
| CC | GO:0005615~extracellular space | 139 | 36.01036 | 2.29E-62 |
| CC | GO:0005576~extracellular region | 125 | 32.38342 | 1.05E-41 |
| CC | GO:0009986~cell surface | 66 | 17.09845 | 6.30E-32 |
| CC | GO:0009897~external side of plasma membrane | 45 | 11.65803 | 8.15E-32 |
| CC | GO:0005886~plasma membrane | 166 | 43.00518 | 9.03E-22 |
| CC | GO:0005887~integral component of plasma membrane | 75 | 19.43005 | 2.01E-14 |
| CC | GO:0070062~extracellular exosome | 108 | 27.97927 | 1.47E-11 |
| CC | GO:0045121~membrane raft | 23 | 5.958549 | 2.11E-10 |
| CC | GO:0031093~platelet alpha granule lumen | 13 | 3.367876 | 7.73E-10 |
| CC | GO:0071556~integral component of lumenal side of endoplasmic reticulum membrane | 10 | 2.590674 | 3.55E-09 |
| CC | GO:0005829~cytosol | 112 | 29.01554 | 1.21E-08 |
| CC | GO:0000790~nuclear chromatin | 20 | 5.181347 | 1.42E-08 |
| CC | GO:0048471~perinuclear region of cytoplasm | 37 | 9.585492 | 1.46E-08 |
| CC | GO:0012507~ER to Golgi transport vesicle membrane | 11 | 2.849741 | 7.42E-08 |
| CC | GO:0042613~MHC class II protein complex | 8 | 2.072539 | 1.70E-07 |
| MF | GO:0005125~cytokine activity | 54 | 13.98964 | 5.05E-46 |
| MF | GO:0005515~protein binding | 273 | 70.72539 | 5.47E-19 |
| MF | GO:0008009~chemokine activity | 18 | 4.663212 | 1.19E-16 |
| MF | GO:0042802~identical protein binding | 56 | 14.50777 | 1.41E-15 |
| MF | GO:0019899~enzyme binding | 36 | 9.326425 | 1.01E-14 |
| MF | GO:0005102~receptor binding | 34 | 8.80829 | 1.65E-12 |
| MF | GO:0008083~growth factor activity | 23 | 5.958549 | 6.77E-12 |
| MF | GO:0008134~transcription factor binding | 28 | 7.253886 | 1.29E-10 |
| MF | GO:0004872~receptor activity | 24 | 6.217617 | 3.68E-10 |
| MF | GO:0031730~CCR5 chemokine receptor binding | 7 | 1.813472 | 2.69E-09 |
| MF | GO:0019901~protein kinase binding | 30 | 7.772021 | 3.61E-09 |
| MF | GO:0002020~protease binding | 16 | 4.145078 | 4.53E-09 |
| MF | GO:0042803~protein homodimerization activity | 43 | 11.1399 | 8.06E-09 |
| MF | GO:0044212~transcription regulatory region DNA binding | 22 | 5.699482 | 8.09E-09 |
| MF | GO:0005031~tumor necrosis factor-activated receptor activity | 9 | 2.331606 | 2.46E-08 |

| **Table 2** KEGG enrichment entry | | | | |
| --- | --- | --- | --- | --- |
| Category | Term | Count | Gene Ratio (%) | PValue |
| KEGG | hsa04060:Cytokine-cytokine receptor interaction | 80 | 20.72539 | 1.47E-47 |
| KEGG | hsa05321:Inflammatory bowel disease (IBD) | 38 | 9.84456 | 1.94E-33 |
| KEGG | hsa05164:Influenza A | 55 | 14.2487 | 4.82E-31 |
| KEGG | hsa05140:Leishmaniasis | 37 | 9.585492 | 8.59E-30 |
| KEGG | hsa04620:Toll-like receptor signaling pathway | 42 | 10.88083 | 5.67E-28 |
| KEGG | hsa05142:Chagas disease (American trypanosomiasis) | 41 | 10.62176 | 3.41E-27 |
| KEGG | hsa05168:Herpes simplex infection | 52 | 13.4715 | 7.65E-27 |
| KEGG | hsa04630:Jak-STAT signaling pathway | 46 | 11.9171 | 6.53E-26 |
| KEGG | hsa05152:Tuberculosis | 50 | 12.95337 | 1.17E-25 |
| KEGG | hsa05162:Measles | 44 | 11.39896 | 1.35E-25 |
| KEGG | hsa05323:Rheumatoid arthritis | 36 | 9.326425 | 1.79E-24 |
| KEGG | hsa05330:Allograft rejection | 25 | 6.476684 | 9.81E-24 |
| KEGG | hsa05145:Toxoplasmosis | 38 | 9.84456 | 8.27E-23 |
| KEGG | hsa05332:Graft-versus-host disease | 22 | 5.699482 | 1.23E-20 |
| KEGG | hsa04940:Type I diabetes mellitus | 24 | 6.217617 | 2.16E-20 |
| KEGG | hsa05161:Hepatitis B | 40 | 10.36269 | 4.73E-20 |
| KEGG | hsa05133:Pertussis | 30 | 7.772021 | 4.94E-20 |
| KEGG | hsa05320:Autoimmune thyroid disease | 24 | 6.217617 | 1.09E-17 |
| KEGG | hsa05144:Malaria | 23 | 5.958549 | 3.85E-17 |
| KEGG | hsa04380:Osteoclast differentiation | 35 | 9.067358 | 4.48E-17 |
| KEGG | hsa04066:HIF-1 signaling pathway | 30 | 7.772021 | 1.19E-16 |
| KEGG | hsa05169:Epstein-Barr virus infection | 33 | 8.549223 | 2.82E-16 |
| KEGG | hsa04668:TNF signaling pathway | 31 | 8.031088 | 3.41E-16 |
| KEGG | hsa05160:Hepatitis C | 34 | 8.80829 | 6.77E-16 |
| KEGG | hsa05166:HTLV-I infection | 46 | 11.9171 | 2.43E-15 |

**Fig. 1** A: The Kyoto Encyclopedia of Genes and Genomes (KEGG) pathway map of Cytokine-cytokine receptor interaction. B: The KEGG pathway map of Toll-like receptor signaling pathway.


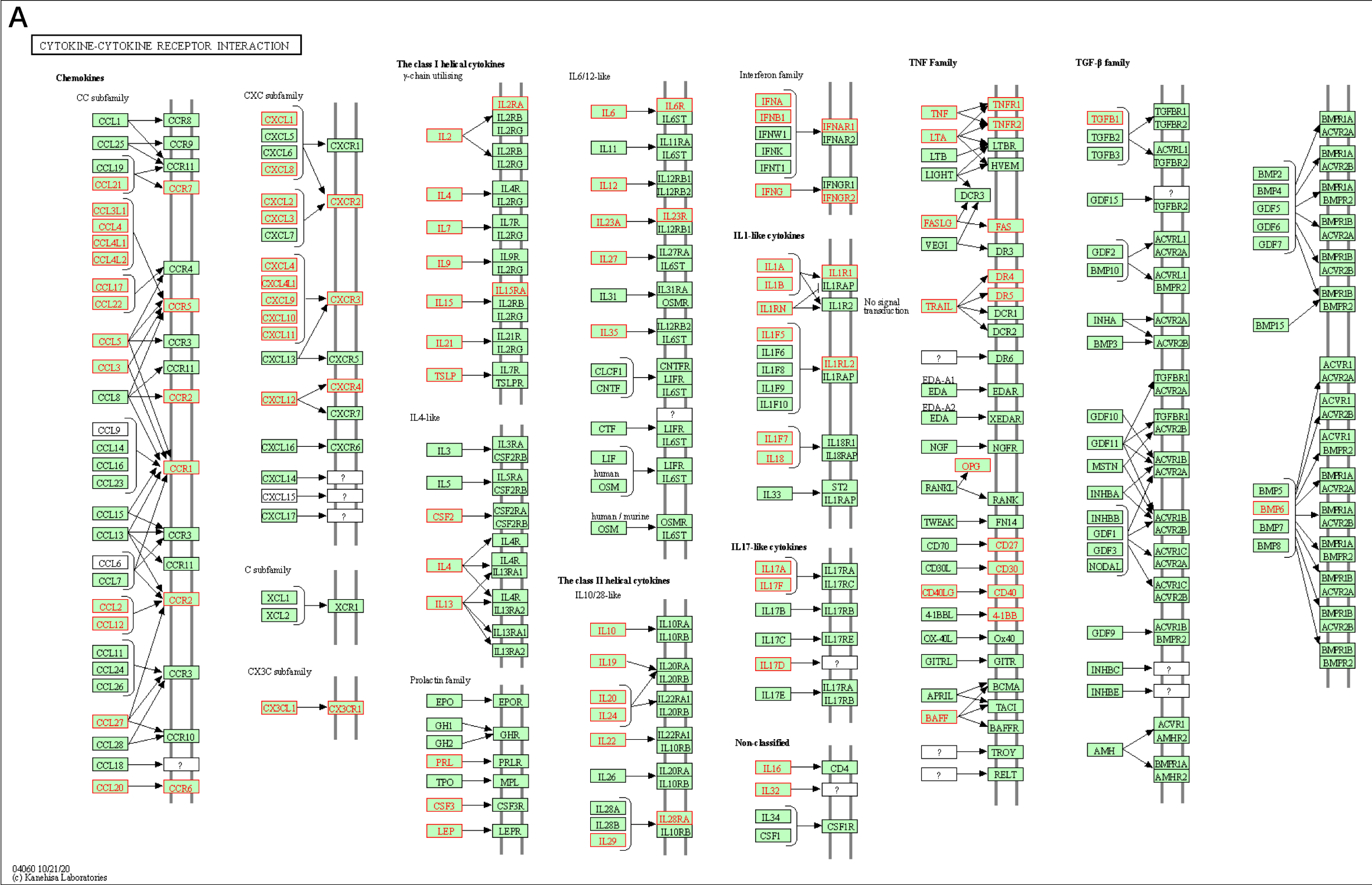


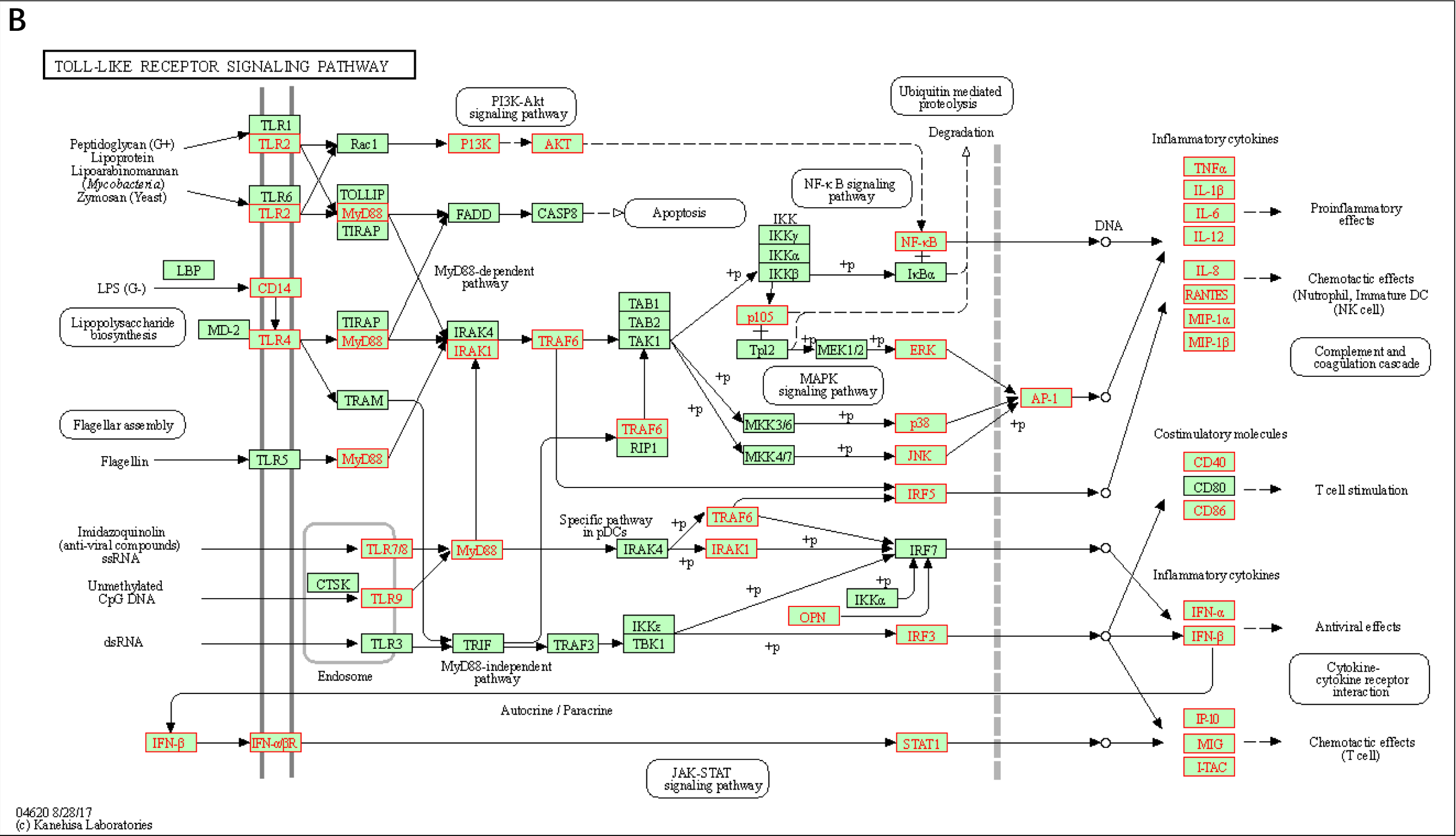

Supplement: Supplementary file 3 — Additional file 3. Functional annotation of overlapping genes between the psoriasis and hepatitis C. [file 12985_2021_1606_MOESM3_ESM.docx]
